# Transposable element sequence fragments incorporated into coding and noncoding transcripts modulate the transcriptome of human pluripotent stem cells

**DOI:** 10.1093/nar/gkab710

**Published:** 2021-08-14

**Authors:** Isaac A Babarinde, Gang Ma, Yuhao Li, Boping Deng, Zhiwei Luo, Hao Liu, Mazid Md Abdul, Carl Ward, Minchun Chen, Xiuling Fu, Liyang Shi, Martha Duttlinger, Jiangping He, Li Sun, Wenjuan Li, Qiang Zhuang, Guoqing Tong, Jon Frampton, Jean-Baptiste Cazier, Jiekai Chen, Ralf Jauch, Miguel A Esteban, Andrew P Hutchins

**Affiliations:** Shenzhen Key Laboratory of Gene Regulation and Systems Biology, School of Life Sciences, Southern University of Science and Technology, Shenzhen 518055, China; Department of Biology, School of Life Sciences, Southern University of Science and Technology, Shenzhen 518055, China; Shenzhen Key Laboratory of Gene Regulation and Systems Biology, School of Life Sciences, Southern University of Science and Technology, Shenzhen 518055, China; Department of Biology, School of Life Sciences, Southern University of Science and Technology, Shenzhen 518055, China; Shenzhen Key Laboratory of Gene Regulation and Systems Biology, School of Life Sciences, Southern University of Science and Technology, Shenzhen 518055, China; Department of Biology, School of Life Sciences, Southern University of Science and Technology, Shenzhen 518055, China; Department of Biology, School of Life Sciences, Southern University of Science and Technology, Shenzhen 518055, China; Institute of Cancer and Genomic Sciences, University of Birmingham, Birmingham B15 2TT, UK; Laboratory of Integrative Biology, Guangzhou Institutes of Biomedicine and Health, Chinese Academy of Sciences, Guangzhou 510530, China; Key Laboratory of Regenerative Biology of the Chinese Academy of Sciences and Guangdong Provincial Key Laboratory of Stem Cell and Regenerative Medicine, Guangzhou Institutes of Biomedicine and Health, Chinese Academy of Sciences, Guangzhou 510530, China; Laboratory of Integrative Biology, Guangzhou Institutes of Biomedicine and Health, Chinese Academy of Sciences, Guangzhou 510530, China; Key Laboratory of Regenerative Biology of the Chinese Academy of Sciences and Guangdong Provincial Key Laboratory of Stem Cell and Regenerative Medicine, Guangzhou Institutes of Biomedicine and Health, Chinese Academy of Sciences, Guangzhou 510530, China; Laboratory of Integrative Biology, Guangzhou Institutes of Biomedicine and Health, Chinese Academy of Sciences, Guangzhou 510530, China; Key Laboratory of Regenerative Biology of the Chinese Academy of Sciences and Guangdong Provincial Key Laboratory of Stem Cell and Regenerative Medicine, Guangzhou Institutes of Biomedicine and Health, Chinese Academy of Sciences, Guangzhou 510530, China; Laboratory of Integrative Biology, Guangzhou Institutes of Biomedicine and Health, Chinese Academy of Sciences, Guangzhou 510530, China; Key Laboratory of Regenerative Biology of the Chinese Academy of Sciences and Guangdong Provincial Key Laboratory of Stem Cell and Regenerative Medicine, Guangzhou Institutes of Biomedicine and Health, Chinese Academy of Sciences, Guangzhou 510530, China; Department of Biology, School of Life Sciences, Southern University of Science and Technology, Shenzhen 518055, China; Shenzhen Key Laboratory of Gene Regulation and Systems Biology, School of Life Sciences, Southern University of Science and Technology, Shenzhen 518055, China; Department of Biology, School of Life Sciences, Southern University of Science and Technology, Shenzhen 518055, China; Shenzhen Key Laboratory of Gene Regulation and Systems Biology, School of Life Sciences, Southern University of Science and Technology, Shenzhen 518055, China; Department of Biology, School of Life Sciences, Southern University of Science and Technology, Shenzhen 518055, China; Department of Biology, School of Life Sciences, Southern University of Science and Technology, Shenzhen 518055, China; Center for Cell Lineage and Atlas (CCLA), Bioland Laboratory (Guangzhou Regenerative Medicine and Health Guangdong Laboratory), Guangzhou 510005, China; Shenzhen Key Laboratory of Gene Regulation and Systems Biology, School of Life Sciences, Southern University of Science and Technology, Shenzhen 518055, China; Department of Biology, School of Life Sciences, Southern University of Science and Technology, Shenzhen 518055, China; Laboratory of Integrative Biology, Guangzhou Institutes of Biomedicine and Health, Chinese Academy of Sciences, Guangzhou 510530, China; Key Laboratory of Regenerative Biology of the Chinese Academy of Sciences and Guangdong Provincial Key Laboratory of Stem Cell and Regenerative Medicine, Guangzhou Institutes of Biomedicine and Health, Chinese Academy of Sciences, Guangzhou 510530, China; Department of Biology, School of Life Sciences, Southern University of Science and Technology, Shenzhen 518055, China; Center for Reproductive Medicine, Shuguang Hospital Affiliated to Shanghai University of Traditional Chinese Medicine, Shanghai 200120, China; Institute of Cancer and Genomic Sciences, University of Birmingham, Birmingham B15 2TT, UK; Institute of Cancer and Genomic Sciences, University of Birmingham, Birmingham B15 2TT, UK; Centre for Computational Biology, University of Birmingham, Birmingham, UK; Key Laboratory of Regenerative Biology of the Chinese Academy of Sciences and Guangdong Provincial Key Laboratory of Stem Cell and Regenerative Medicine, Guangzhou Institutes of Biomedicine and Health, Chinese Academy of Sciences, Guangzhou 510530, China; Center for Cell Lineage and Atlas (CCLA), Bioland Laboratory (Guangzhou Regenerative Medicine and Health Guangdong Laboratory), Guangzhou 510005, China; Joint School of Life Sciences, Guangzhou Medical University and Guangzhou Institutes of Biomedicine and Health, Chinese Academy of Sciences, Guangzhou, China; School of Biomedical Sciences, Li Ka Shing Faculty of Medicine, The University of Hong Kong, Hong Kong SAR, China; Laboratory of Integrative Biology, Guangzhou Institutes of Biomedicine and Health, Chinese Academy of Sciences, Guangzhou 510530, China; Key Laboratory of Regenerative Biology of the Chinese Academy of Sciences and Guangdong Provincial Key Laboratory of Stem Cell and Regenerative Medicine, Guangzhou Institutes of Biomedicine and Health, Chinese Academy of Sciences, Guangzhou 510530, China; Bioland Laboratory (Guangzhou Regenerative Medicine and Health Guangdong Laboratory), Guangzhou 510005, China; Shenzhen Key Laboratory of Gene Regulation and Systems Biology, School of Life Sciences, Southern University of Science and Technology, Shenzhen 518055, China; Department of Biology, School of Life Sciences, Southern University of Science and Technology, Shenzhen 518055, China

## Abstract

Transposable elements (TEs) occupy nearly 40% of mammalian genomes and, whilst most are fragmentary and no longer capable of transposition, they can nevertheless contribute to cell function. TEs within genes transcribed by RNA polymerase II can be copied as parts of primary transcripts; however, their full contribution to mature transcript sequences remains unresolved. Here, using long and short read (LR and SR) RNA sequencing data, we show that 26% of coding and 65% of noncoding transcripts in human pluripotent stem cells (hPSCs) contain TE-derived sequences. Different TE families are incorporated into RNAs in unique patterns, with consequences to transcript structure and function. The presence of TE sequences within a transcript is correlated with TE-type specific changes in its subcellular distribution, alterations in steady-state levels and half-life, and differential association with RNA Binding Proteins (RBPs). We identify hPSC-specific incorporation of endogenous retroviruses (ERVs) and LINE:L1 into protein-coding mRNAs, which generate TE sequence-derived peptides. Finally, single cell RNA-seq reveals that hPSCs express ERV-containing transcripts, whilst differentiating subpopulations lack ERVs and express SINE and LINE-containing transcripts. Overall, our comprehensive analysis demonstrates that the incorporation of TE sequences into the RNAs of hPSCs is more widespread and has a greater impact than previously appreciated.

## INTRODUCTION

Transposable elements (TEs) are a heterogeneous collection of DNA sequences that, when active, are capable of movement to different positions within a genome, often through a replicative mechanism. During evolution, TEs have increased their copy numbers through extensive transposition and duplication and now make up nearly 40% of mammalian genomes (1,2). However, the vast majority of TEs in the human genome are mutated, fragmentary, and incapable of transposition. Nonetheless, there is growing evidence that inactive TEs have functional roles in both normal cellular processes and disease. For example, they can participate in onco-exaptation events, in which regulatory motifs within TE sequences are recruited to drive oncogene expression (3). The presence of TE sequences within transcripts can also influence alternative splicing (4), and TE expression is positively correlated with developmental competency and evolutionary innovation (5–10). In somatic cells, TEs are mainly thought to be silenced by DNA methylation and the histone mark H3K9me3 (11,12). However, in the mammalian embryo, DNA is demethylated, and human pluripotent stem cells (hPSCs) have reduced levels of repressed chromatin, creating a more permissive environment for transcription, including of TE sequences (13,14). Consequently, more TE-containing RNAs are detected during embryogenesis and in hPSCs, compared to somatic cells (8), and are expressed in a stage-specific manner during embryogenesis (15,16).

The RNA sequences of long-noncoding RNAs (lncRNAs) are rich in TE fragments (17–20), and whilst lncRNAs have roles in normal biological processes (9,21,22), and disease etiology (23,24), the contributions of TE sequences within lncRNAs has received less attention. One model suggests that the TE fragments inside the lncRNAs act as independently folded domains, something akin to globular domains of proteins (25,26). Interestingly, the incorporation of TE sequences into the mRNAs of normal pluripotency transcripts has been observed in cancerous cells (3), suggesting that their presence may be causally connected to human disease. Consequently, it is important to understand how TEs contribute to the coding and noncoding transcriptome (27–29), particularly if hPSCs are to be used in cell replacement therapy (8,30). However, due to limitations in short read sequencing, which include difficulty in identifying structural variants and problems with assembling full-length mRNAs and lncRNAs, accurate transcript maps have been difficult to achieve (31,32). The relatively low expression levels of lncRNAs and the fact that TE sequences are repeated throughout the genome presents additional challenges (33).

To explore the contribution of TE sequences to the transcriptome in a normal non-diseased state, we took advantage of the large number of hPSC short read RNA-seq samples, which we supplemented with long read RNA-seq. Our analysis shows that TEs are incorporated into both coding and noncoding RNAs, and their presence is correlated with lower levels of steady-state transcript accumulation compared to TE-free transcripts. The presence of TE sequences within RNAs also led to effects on the distribution of coding and noncoding transcripts between the nucleus and cytoplasm, RNA half-life, and differential binding of RBPs to transcripts. Whilst TE sequence fragments could be found inside predicted ORFs (open reading frames), ribosomes were bound to the RNA and peptides were detected in only a few cases. The one exception was the relatively large number of endogenous retroviral protein-derived peptides. Finally, using single-cell RNA-seq we show that transcripts containing different TE-types are present in distinct subpopulations of hPSCs. The hPSC-state is dominated by HERVH and LTR7-containing transcripts, and upon differentiation, HERVH-containing transcripts decline, and SINE and LINE-containing transcripts become more common.

## MATERIALS AND METHODS

### Cell lines, RNA extraction, PCR and long read RNA-seq

The cell lines used in this study were hESCs (H1 and WIBR3 line) and iPSCs (c11/S0730 line; (34)). These cell lines were grown in mTeSR1 (Stemcell technologies: 85850) on pre-coated matrigel plates (Corning: 354277). The medium was replaced every 24 h. Cells were passaged by single-cell digestion with Accutase (SIGMA: A6964) every 5 days. Total RNA was extracted using Trizol (MRC: RN190). The concentration of the extracted RNA was measured using a Nanodrop. For long read RNA-seq, we first confirmed the cells were not differentiated by qRT-PCR (quantitative real time polymerase chain reaction) of marker genes such as *SOX2* and *NANOG*. Long read sequencing for the two cell lines was performed in duplicates. For PCR of selected transcripts, 1 ug of extracted RNA was reverse-transcribed using the PrimeScript RT Master Mix (Taraka: RR036A). Thereafter, cDNA samples were amplified by real-time PCR using TB Green™ Premix Ex Taq™ II (Taraka: RR820A) to saturation (40 cycles) with the primers listed in Supplementary Table S1. The final PCR products were separated on an Agarose gel.

### Source of the short read RNA-seq data

The short reads (SR) were obtained from the Sequence Read Archive (SRA) or from the European Nucleotide Archive (ENA). In total, we found 317 publicly available SR datasets of wild-type unperturbed hESC and iPSC samples, of which 150 could pass stringent quality control criteria (see results). The accession numbers, number of reads, read lengths, inset sizes and library sizes (in base pairs) for each sample is presented in Supplementary Table S2.

### Transcript assembly

Transcripts were first assembled independently using SR and LR sequencing data and then combined to form a consensus assembly. The SR were mapped to the human genome hg38 assembly using the SR aligner HISAT2 (35), and the aligned reads were merged using samtools (36). StringTie (37) was used to assemble the transcripts from the merged aligned reads, guided by the GENCODE v32 annotations. Transcripts with no inferred strand were discarded. For each LR sample, consensus sequences were first generated from subread data using *ccs* (38). Next, *lima* was used to generate full-length reads by primer removal and demultiplexing. Noise from full-length reads was removed using isoseq3. The denoised alignment files were converted to FASTA format using bamtools (39). Noise-free full-length reads were then aligned to the human genome hg38 assembly using the LR compatible aligner GMAP (40). The alignments from the four samples were merged using samtools. StringTie was then used to assemble the transcripts from the alignments. As with SR, any transcript that could not be assigned to a strand was discarded. We then merged the SR and LR transcripts. The RNA abundance of the transcripts were then computed from the SR alignments, quantified by StringTie. Example code is in the Supplementary Methods.

### Detection of TE sequence fragments inside transcripts

For each transcript, the FASTA sequence was extracted for the assembled transcript. An edited version of the Dfam (41) database of TE HMMs (hidden Markov models) was used, with all non-primate TE families removed. nhmmer was then used to search against the transcript assembly, with the settings ‘-e 1e-10, –dna’. As nhmmer can discover multiple TE types overlapping the same coordinates (for example, SINE and SVA family-members are often annotated to similar locations as they share parts of their sequences), overlapping TEs were removed to leave a single TE based on a progression of criteria: (i) If two domains entirely contained each other, then the TE with the lowest E-value was kept. (ii) If the percentage of overlap between two pairs of TEs was >60%, then the domain with the best E-value was retained and the other TE deleted. This was repeated iteratively across all pairs of overlapping domains until both conditions were satisfied. The final Supplementary Table of TE-containing transcripts is in Supplementary Table S3.

### Analysis of RBP data

Enhanced crosslinking and immunoprecipitation (eCLIP) data for the RBPs DDX6, ILF2, FUS, DCP1B and matching input data (42) were downloaded from the SRA. Adapters were removed by fastp. The reads were mapped to the human hg38 genome assembly by STAR (43). The peaks were then called by MACS2 using default parameters (44). For each group of transcripts, the percentage of transcripts with an eCLIP RBP peak was computed.

### RNA subcellular distribution

The RNA subcellular distribution data for H1 hESCs for the nucleus and cytosol were obtained from the ENCODE database (http://hgdownload.cse.ucsc.edu/goldenPath/hg19/encodeDCC/wgEncodeRikenCage). The reads were aligned to the human hg38 genome with HISAT2. The same pipeline for SR RNA-seq quantification using StringTie, described above, was then used for quantification. We then computed the relative concentration index (RCI) for each transcript using a previously reported formula (45). RCI was computed as follows:

$$\begin{eqnarray*} RCI = {log_2}\left(\frac{{TPM_{cytosol}}+0.001}{{TPM_{nucleus}}+0.001}\right) \end{eqnarray*}$$

### RNA half-life

Data was downloaded from the SRA (GSE156671). The data included RNA-seq data at 0, 1, 2, 4 and 8 h after treatment with actinomycin D. Adapters were removed by fastp. The reads were aligned to the human hg38 genome with HISAT2. The same pipeline for SR RNA-seq quantification using StringTie was used for quantification. For each transcript at each time point (*t*, h), the relative RNA abundance was computed as the log2 fraction of the TPM at time *t* versus the 0 h TPM.

### Evolutionary analysis of TE and TE-free sequences inside transcripts

The PhyloP (46) track for primates (hg38.phastCons17way.wigFix.gz) was downloaded from the UCSC genome browser and used as a score for evolutionary conservation. The PhyloP-primate track contains a score for each base pair of the genome which measures the rate of nucleotide substitution compared to neutral drift. A positive score represents a decrease in nucleotide substitution (i.e., conservation), and a negative score represents an increase in the accumulation of mutations (i.e. acceleration).

### DeepCAGE and polyadenylated data analyses

Human ESC and iPSC deepCAGE alignments that sequenced the 5′ ends of transcripts were retrieved from the FANTOM5 database (http://fantom.gsc.riken.jp/5/datafiles/reprocessed/hg38_latest/basic/human.timecourse.hCAGE/) (47). The three biological replicates each from hESC and iPSC samples were merged and indexed using samtools. The bam files were then converted to wiggle files using deeptools bamCoverage (48). Then the wiggle file and the transcript files were used to compute a matrix using deeptools computeMatrix. The matrices were plotted using deeptools plotHeatmap. For the read coverage, regions covering 100bp upstream and downstream of the transcription start sites (TSS) of hPSC transcripts were extracted. The estimation of the number of reads mapped to each region in the two merged alignments was done using deeptools multiBamSummary. To estimate the number of transcripts with deepCAGE support, the number of reads that mapped to 500 bp upstream and downstream of transcription start site (TSS) of each transcript was computed using deeptools multiBamSummary. Transcripts with at least 0.1 counts per million aligned reads (CPM) were considered as supported by deepCAGE data. Human polyadenylated data (polyA-seq) (49) was retrieved from the SRA. The data was aligned to the human hg38 genome with bwa (50). The aligned data was sorted and indexed by samtools. The data was then subjected to an analysis pipeline similar to that of the deepCAGE data, except that the focus was on the 3′ end of the transcript.

### Human pluripotent stem cell enrichment of the assembled transcripts

We downloaded representative RNA-seq samples from human somatic cell types and tissues from the SRA or the ENA databases. We used only samples with paired-end reads, and at least 10 million reads and with an alignment rate of at least 70%. In total, 174 samples from 63 different human tissue and cell types were used (see Supplementary Table S1 for the accession numbers, cell type tissue names, number of reads and read lengths). The sequence data was aligned to the human genome using HISAT2 (51). The expression levels (in TPM) were computed for each sample, using the same StringTie quantification that was used for SR hPSC RNA-seq data. The *Z*-score was then computed for the expression level of each hPSC transcript using the mean and the standard deviation computed from panel of somatic samples. The top 25% of transcripts with the highest *Z*-score were classified as ‘enriched’, with a *Z*-score of at least 0.66. Transcripts with a *Z*-score <–0.66 were classified as ‘depleted’, while those with –0.66 ≤ *Z*-score ≤0.66 were classified as ‘nonspecific’.

### Transcript coding potential measurement, and mass spectrometry data processing and analysis

The coding potential of the transcripts was assessed with FEELnc (52). The transcripts of all protein-coding and lincRNA biotypes from the GENCODE transcript assembly were used as the training data set for FEELnc. Using the training dataset, FEELnc decided on a coding potential threshold of 0.432 for protein-coding transcripts. The trained model was then applied to our hPSC-specific assembly to produce a coding or noncoding prediction. Note that FEELnc did not report a prediction for 13 transcripts and they were reported as ‘NA’.

To analyze the mass spectrometry (MS) data, we used the set of transcripts that contained a TE and had a predicted coding sequence (CDS), and then used criteria to exclude known proteins or fragments of known proteins. FEELnc predicts whether a transcript has a putative CDS, but does not predict the most likely ORF in the RNA sequence. Hence, we measured the longest ORF for each transcript. Many ORFs are incomplete and lack a STOP codon (52). Hence, if there was an ATG and the ORF extended to the end of the transcript, we would add an in-frame STOP codon and the ORF would be measured from the ATG to the end of the transcript. This protocol was also used for the ORFs/CDSs in the GENCODE annotations, which are not always complete and would sometimes lack a STOP codon. We then compared the ORFs determined by our strategy to the ORFs reported in GENCODE. In total 85% of our transcript ORFs matched perfectly to the annotated ORF in the GENCODE transcript when a matching transcript was available. Hence, we used the GENCODE ORF annotation, when available, and our ORF prediction for all other transcripts.

To detect novel peptides, we performed several filtering steps. First, we removed ORFs that matched perfectly to a GENCODE ORF. This process, however, was not always correct, as we noticed that the GENCODE annotation for the location of the CDS was not always accurate. Hence, to strictly exclude ORFs that matched to GENCODE, we also removed any CDS that resulted in a BLAST hit against the GENCODE peptide database with > 90% identity (*E*-value < 1e–20) for the full-length protein. Next, to restrict the search to only novel peptide sequences, we masked out any peptide sequence fragments in a predicted ORF with >90% (*E*-value < 1e–20) identity with any fragment of a protein from GENCODE. This would remove instances of ORFs that match part of a known protein but have novel peptide sequences inside. We further deleted any proteins with <20 unmasked amino acids, as short peptides are unlikely to be detected in the MS data, and any proteins that did not contain at least one K or R amino acid, as the search algorithm only considers a peptide match with at least one cleaved terminus. The raw MS data from the HipSci project was first converted to centroid data using msconvert, a part of the MSGF+ (53). MSGF+ was then used to search peptide spectra, with the same peptide modification parameters used in the HipSci project (54,55): Carbamidomethylation on cysteine as a fixed modification, and the variable modifications: oxidation on methionine, conversion of N-terminal glutamine to pyro-glutamine, deamidation of asparagine and glutamine, and acetylation at the N-terminus. Other parameters used by MSGF + include: setting the digest enzyme to Lys-C, precursor mass tolerance of 20 ppm, and isotope error range of ‘1, 2’. A peptide was considered a hit if the *E*-value reported by MSGF + was <0.001. Peptide hits for transcripts are in Supplementary Table S5. Example computer code is reproduced in the Supplementary Methods.

### Single cell RNA-seq and analysis

Single cell RNA-seq (sc-RNA-seq) was performed on the 10× chromium according to the manufacturer's instructions, using one sample from H1 hESCs and one sample from c11/S0730 iPSCs. We supplemented this data with sc-RNA-seq data from WTC cells from E-MTAB-6687 (56), and two UCLA1 hESC line samples from GSE140021 (57). Both studies also used the 10x Chromium single cell platform. As 10x-based sc-RNA-seq is biased to the 3′ ends of transcripts, we reduced the set of total transcripts to only their unique 3′ ends (within 200 nucleotides on either side of the 3′ end of the transcript). When transcripts shared an overlapping 3′ end, only one of the ends was kept. This reduced the number of transcripts to 88520 (87%) out of the total set of 101479 transcripts. The sc-RNA-seq reads were aligned to the hg38 genome with STARsolo (43), using the appropriate whitelist barcode file, and processed using scTE (58). The matrices were filtered and analyzed using SCANPY (59). To remove unreliable cells, those with less than 1500 genes or 3000 UMI counts were deleted. Similarly, cells with more than 8500 genes or 50 000 UMI counts were deleted as these may represent doublet cells in a single drop, rather than single cells. Only transcript 3′ends that could be detected in >100 cells were retained. The resulting data was normalized using SCRAN (60). UMAPs were generated based on the first 20 principal components, and Leiden clustering was performed to identify subpopulations of cells. Differential expression was called using the rank_genes_groups SCANPY function, with the settings: ‘method = ’t-test_overestim_var', n_genes = 10000’. Significantly different genes were kept if their FDR corrected *q*-value was <0.05 and they were >2-fold enriched in any cluster. Cell cycle was estimated using the gene sets from (61), processed using the SCANPY function score_genes_cell_cycle.

### Code availability and supplementary material

The full code tree for the analysis presented in this paper can be found at: https://github.com/oaxiom/hesc_lincrna. Example software code for key parts of the analysis can also be found in the Supplementary Methods. The code requires glbase3 (https://github.com/oaxiom/glbase3) to run (62).

## RESULTS

### Transcript assembly from short and long reads

To build an hPSC transcriptome, we started with 197 publicly available short read (SR) paired-end-only hPSC RNA-seq data samples. To quality control the data, we began by mapping the hPSC samples to the hg38 genome assembly using HISAT2 (35). As a low mapping rate is suggestive of problems in library preparation or sequencing (63), we removed samples with a mapping rate of less than 70%, leaving 171 RNA-seq samples (Supplementary Figure S1A). There are widespread errors in metadata annotations of publicly available transcriptome data (64). To ensure that the samples were undifferentiated hPSCs, we analyzed gene-level expression (65). hPSC samples were removed if they passed all three requirements: (i) must correlate (Pearson *R*^2^ > 0.6) with other hPSC samples, (ii) must express hPSC-marker genes or (iii) must have low levels of differentiation-specific genes (Supplementary Figure S1B–D). This left 150 samples that passed our quality control criteria (Supplementary Table S2, and Supplementary Figure S1D). We used the 150 qualified hPSC samples to assemble transcripts using a pipeline based on the SR aligner HISAT2 and the transcript assembler StringTie (35,37) (Supplementary Figure S1E). In total, we processed ∼5.5 billion paired-end reads and nearly 1 trillion nucleotides of sequence, with a median mapped fragment size of 225 bp of which 92% could be aligned to the hg38 genome assembly. StringTie (37) assembled an initial set of 279051 transcripts. Short transcripts of less than 200 nucleotides require specialized techniques for processing and sequencing using SR-based protocols (66). Hence, we excluded transcripts less than 200 nucleotides in length, leaving 272268 raw transcripts (Supplementary Figure S1F).

Transcript assembly from SR can be problematic (32,67), especially if the transcripts contain TE sequences (31). Indeed, the raw transcript assembly had a large number of single-exon fragments (72902 out of 272268; 27%), and although some of these fragments might be genuine single-exon transcripts, many are likely to be fragments of larger RNAs produced during sample preparation, or reflect failures by the transcript assembler (StringTie) to join the fragments due to gaps in the sequences. To improve the quality of the transcript assembly, we augmented our SR-based transcripts with SMRT long read (LR) sequencing generated using the PacBio platform. We sequenced RNA extracted from the hESC cell line H1 and the iPSC cell line S0730 (34) in duplicate. Transcripts were identified from long reads using the isoseq pipeline and were aligned to the genome using the long read capable aligner GMAP (40) (Supplementary Figure S1E). To be consistent with the SR-assembled transcripts, and as we did not take any special measures to include short transcripts in the LR experiments, we also removed transcripts <200 nucleotides in length. Overall, the LR assembly produced 53168 unique transcripts (Supplementary Figure S1F).

Both SR-based and LR-based transcriptomes have advantages and disadvantages: SR-based have high dynamic range, but transcript assemblies tend to be unreliable (32), while LR-based assemblies can detect extremely rare transcripts but are poor at quantifying RNA levels. Consequently, to arrive at a transcriptome representation, we set two requirements for transcript abundance: (i) RNA abundance ≥0.1 TPM (transcripts per million) in at least 50 of the SR samples. (ii) The average per-base coverage reported by StringTie must be >1 for all exons. Finally, we deleted single-exon transcripts that appeared to be intron fragments from splicing, if their exon edges showed a near-perfect match with an annotated intron (Supplementary Figure S1G). The final assembly contained 101492 transcripts, of which 13177 (13%) were single-exons. Using these criteria, 71% of the LR transcripts were retained, but only 17% of the SR transcripts were kept in the final assembly (Supplementary Figure S1F and H).

**Figure 1.**
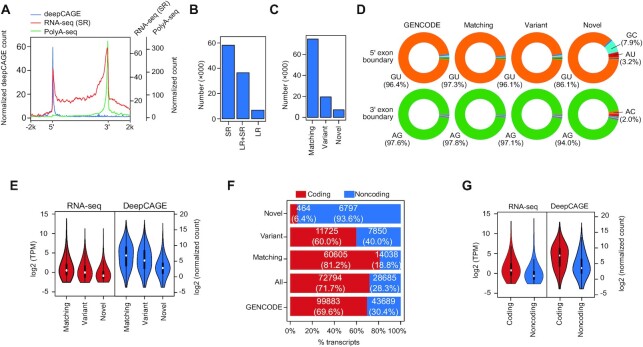
Combining short read RNA-seq and long read RNA-seq to assemble hPSC-specific transcriptome. (**A**) Read density of different experimental techniques across the length of the transcript. Pileups of hPSC data from deepCAGE, short read RNA-seq, and polyA-seq reads across the lengths of the transcripts from the 5′ ends to the 3′ ends and the flanking 2 kb regions. Each transcript is scaled to the same size and orientated to the same strand. DeepCAGE specifically sequences the 5′ ends of transcripts and can identify TSSs. DeepCAGE data is measured in normalized tag counts, taken from Ref. (47). PolyA-seq data is from the 3′ RNA-seq data set GSE138759 (49), and is measured in normalized counts. RNA-seq (SR) refers to pileups of the SR RNA-seq data only, across the transcripts. The SR sample accessions used in this study are described in Supplementary Table S1. (**B**) The number of transcripts (in thousands) that are supported by short read (SR)-only, long read (LR)-only, or both (SR + LR). (**C**) The number of transcripts (in thousands) that were defined as matching (all internal exons boundaries match exactly to a GENCODE transcript, exact 5′ and 3′ ends of the transcript are not enforced), variant (shares any exon or overlapping exon segment with a GENCODE transcript) or novel (does not share any exonic nucleotide with a GENCODE transcript). (**D**) Pie charts showing the proportion of nucleotide sequences at the 5′ or 3′ splice sites. The transcripts are divided into the matching, variant or novel classes and all GENCODE transcripts are shown for comparison. (**E**) Violin plots showing normalized RNA counts for matching, variant and novel transcripts, for RNA-seq (from short read data) and deepCAGE data. RNA-seq is presented in log2 transcripts per million (TPM). DeepCAGE is in log2 normalized tag counts, as deepCAGE data only sequences the 5′ ends, only transcripts with unique 5′ ends were used in the analysis of deepCAGE data. (**F**) Number and percentage of coding and noncoding transcripts by transcript class. Coding and noncoding here refers to the prediction by FEELnc. Novel transcripts have no overlapping exons with GENCODE, variants overlap by any single base pair against the GENCODE annotations, matching have exactly matching internal exon splicing sites. ‘All’ are all assembled hPSC transcripts. (**G**) RNA levels of coding and noncoding transcripts, for short read RNA-seq (left violins) or deepCAGE data (right violins). For deepCAGE, only transcripts with a unique 5′ end were used.

To validate our assembly pipeline, we performed PCR with primers spanning an intron (Supplementary Table S1). Out of 40 novel transcripts examined, 31, including eight out of nine long read transcripts were detected (Supplementary Figure S2A). Of the 5′ ends of our transcript assembly, 95% had iPSC or hESC deepCAGE support, whilst 88% of the transcripts had polyA-seq data support (47,49) (Figure 1A, and Supplementary Figure S2B, C). This suggests the 5′ends are reasonably complete, but the 3′ends are less accurate, although it should be noted the polyA-seq data used here was relatively shallow (∼8M mapped tags). Potentially the 3′ ends may be less accurate due to alternative polyadenylation events that the LR and SR do not fully capture. There may also be cell line-specific effects. Our transcript assembly used H1 hESCs and c11/S0730 iPSCs, but the deepCAGE data was from H1 and H9 hESCs, and an unidentified iPSC line (47), and the polyA-seq data was from H1 hESCs alone (49). Our final transcript assembly contained 101479 transcripts, of which 58201 had SR support, 6881 had LR support and 36410 had both SR and LR support (Figure 1B and Supplementary Table S3).

### Identification of novel transcripts and isoforms

We next compared our hPSC transcript assembly to the GENCODE transcriptome to identify known and novel transcripts and genes. We defined three categories: matching (all internal exon/intron boundaries match a transcript annotated by GENCODE with at least 75% overlap of total exon length); variant (with an exon overlapping any exon from a GENCODE transcript but not necessarily matching splice sites) and novel (with no exon overlapping any GENCODE exon) (Figure 1C). Whilst most transcripts matched the splicing pattern of a GENCODE transcript, 26836 out of 101492 transcripts were variant or novel (Figure 1C). Analysis of the exon boundaries showed that matching and variant transcripts had canonical GU/AG splice signals at 96–97% of exon boundaries, which closely matched the proportion in GENCODE (∼96%) (Figure 1D). Novel transcript exons had ∼86–94% canonical splice site sequences, and there was an increase in GC (7.9%) and AU (3.2%) nucleotides at the 5′ exon boundary (Figure 1D). For each assembled transcript, we defined completeness as the percent of exons or splice sites that precisely matched the genomic locations of the closest GENCODE exons and splice sites (Supplementary Figure S2D). Transcripts supported by both LR and SR tend to have higher GENCODE exon and splice site completeness (Supplementary Figure S2E), although there remains a sizeable number of transcripts supported only by SR, suggesting that our LR data set has not saturated the transcriptome (Supplementary Figure S2F). The matching transcripts tended to be more highly expressed than novel transcripts, as measured by both RNA-seq and deepCAGE data (Figure 1E).

### A census of coding and noncoding transcripts enriched in pluripotent stem cells

To define coding and noncoding transcripts we used FEELnc to computationally predict coding potential. FEELnc is a machine learning algorithm that uses sequence signatures in the RNA to assess coding potential, particularly k-mer frequencies in complete and incomplete ORFs inside transcripts (52). FEELnc determined an automatic threshold (0.432) to call a transcript coding or noncoding (Supplementary Figure S2G). Note that FEELnc did generate a prediction for 13 transcripts. Overall, 28699 out of 101479 (28%) were predicted to be noncoding, and the majority (6797 out of 7261, 94%) of novel transcripts were noncoding (Figure 1F). Conversely, the majority (60605 out of 74643; 81%) of the GENCODE-matching transcripts were predicted to be protein-coding. As observed in previous studies (51,68), the expression levels of lncRNAs were lower than protein-coding transcripts (Figure 1G, and Supplementary Figure S2H).

**Figure 2.**
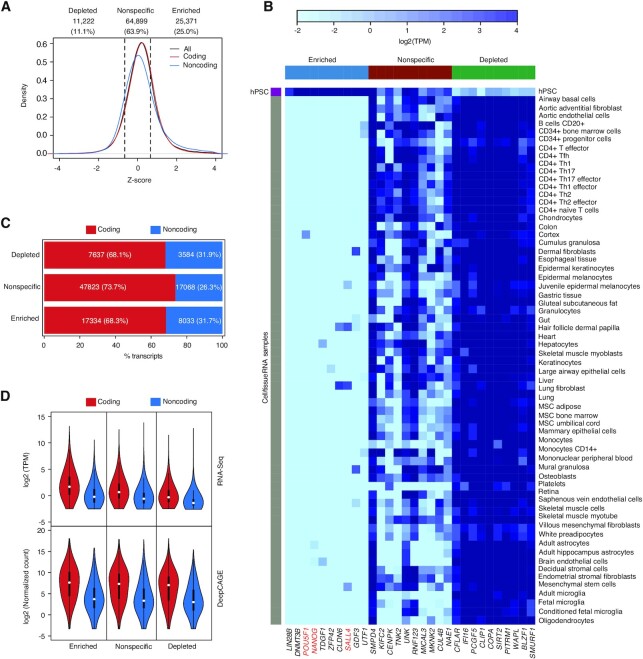
Determination of hPSC-enriched and depleted transcripts. (**A**) *Z*-score of all, coding, and noncoding transcripts against a panel of human somatic (non-embryonic) cell types and tissues. Details of the cell types and tissue samples used are in Supplementary Table S1. The dashed lines indicate the *Z*-score thresholds which represent the top and bottom quartiles that were used to define the hPSC-enriched, hPSC-depleted categories. All other transcripts are considered hPSC-nonspecific. (**B**) Heatmap showing expression of selected hPSC-enriched, -nonspecific and -depleted transcripts in hPSCs and a panel of somatic cell types and tissue samples. RNA abundance is presented as log_2_ TPM. Several known pluripotent marker genes are indicated in red in the hPSC-enriched categories, including the key pluripotency transcription factors *POU5F1* (OCT4), *NANOG* and *SALL4*. (**C**) Percent of the coding and noncoding transcripts in the indicated hPSC expression categories. (**D**) Violin plots showing the RNA-seq expression levels (top panel; log_2_(TPM) transcripts per million) or deepCAGE data (bottom panel; in normalized read counts) for the indicated expression classes for coding and noncoding transcripts.

Our sequencing depth is large, meaning we can assemble very rare transcripts, and hPSC cultures are not homogenous and typically contain small numbers of spontaneously differentiating cells, which means our transcript assembly may contain rare transcripts from differentiated cells. To identify transcripts that are specific to hPSCs versus those that are depleted (more likely to be expressed at higher levels in other cell types), we compared our transcript assembly to a panel of non-embryonic somatic RNA-seq datasets (detailed in Supplementary Table S1). Based on the *Z*-score, transcripts were divided into hPSC-enriched (top quartile), hPSC-depleted (bottom quartile), or hPSC-nonspecific (all other transcripts) (Figure 2A, and Supplementary Figure S2I). This approach could recover known hPSC-enriched transcripts such as *NANOG*, *POU5F1* and *SALL4* (Figure 2B). There was no bias in the proportion of coding and noncoding transcripts in the hPSC expression categories (Figure 2C). As expected, novel transcripts were more likely to be enriched in hPSCs as our transcripts were assembled from hPSC samples (Supplementary Figure S2I). Finally, noncoding transcripts had a lower level of expression compared to coding transcripts whether they were enriched or nonspecific to hPSCs (Figure 2D). Using LR and SR we have assembled a transcriptome for hPSCs, that describes known, variant, and novel transcripts, coding and noncoding, and hPSC-enriched and -depleted transcripts. Example genome views of the transcript classes are shown in Supplementary Figure S3. We will use this transcriptome to explore how TEs are associated with transcript properties.

### TEs are incorporated into the mRNAs of protein-coding genes and modulate expression levels and distribution between the cytoplasm and nucleus

**Figure 3.**
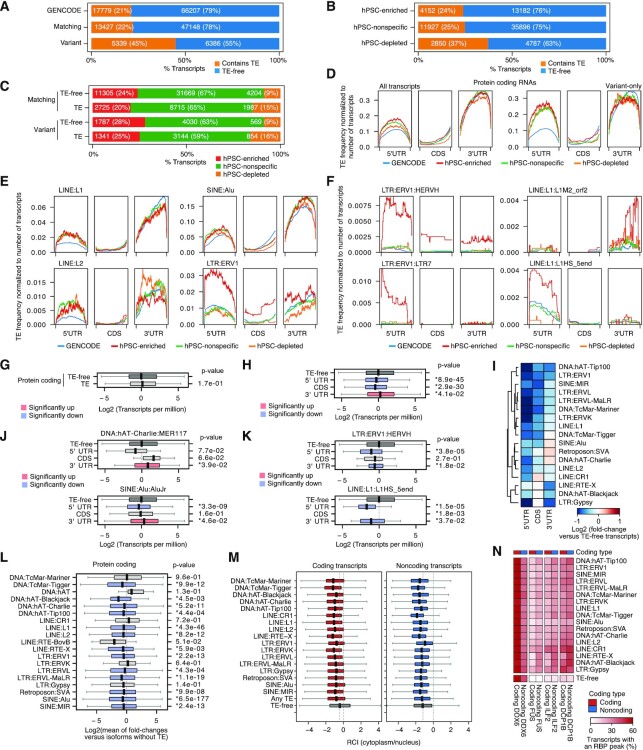
TE sequences in coding transcripts influences RNA steady state levels, subcellular distribution and RBP binding profile. (**A**) Bar plots showing the proportion of protein-coding transcripts that have at least 1 TE or are TE-free, separated into all GENCODE transcripts, or hPSC transcripts matching (a perfect internal exon match to a GENCODE transcript), or variant (any exonic overlap with a GENCODE transcript). (**B**) Bar plots showing the proportion of coding transcripts containing 1 or more TEs, or TE-free, that are enriched, nonspecific or depleted in hPSCs. (**C**) Bar plots showing the proportions of matching or variant transcripts containing a TE, or TE-free that are enriched, nonspecific or depleted in hPSCs. (**D**) Line plots of TE frequency for the indicated classes of hPSC expression or for all GENCODE transcripts. Transcripts were divided into the UTRs and CDS, scaled to a uniform length, and the TE frequency was normalized to the total number of transcripts. The left plots show all transcripts, and the right shows variant transcripts only. (**E**) TE density plots (as in panel D) for protein-coding transcripts containing LINE:L1, LINE:L2, SINE:Alu and LTR:ERV1 TEs. (**F**) TE density plots (as in panel D) for protein-coding transcripts containing the indicated LTR, LINE, and SINE sub-types. (**G**) Box plot for the RNA levels of protein-coding transcripts with 1 or more TEs or without a TE. p-value is from a two-sided Welch's t-test for TE-containing versus TE-free transcripts. The boxplots indicate the upper and lower quartiles, and the whiskers indicate the ranges of the data, for this and subsequent boxplots. (**H**) Transcripts were divided based on the presence of a TE sequence in the UTRs or CDS. Note that transcripts can occupy multiple categories. p-values are from a two-sided Welch's *t*-test for TE-free versus transcripts with TEs in their UTRs, or CDS. (**I**) Heatmap showing the mean fold-change of transcripts containing one or more of the indicated TE subtypes in their UTRs or CDS versus TE-free transcripts. (**J**) Box plot showing the mean expression of transcripts containing a HERVH or L1HS_5end in the UTRs or CDS compared to all TE-free transcripts. Note that there were no transcripts with a fragment of L1HS_5end inside the CDS. p-values are from a two-sided Welch's t-test for each TE type versus TE-free transcripts. (**K**) As in panel J, but for transcripts with one or more fragments of MER117 or AluJr. (**L**) Effect of TEs on RNA levels for different isoforms of the same gene. Isoforms of the same gene were merged, and the fold-change was calculated for the TE-containing versus the TE-free isoforms. The boxplots show the spread of fold-changes for all genes that have at least one TE-free isoform and at least one TE of the indicated type. p-values are from a two-sided one-sample *t*-test. (**M**) Subcellular RNA distribution of coding (left) and noncoding (right) transcripts, as measured by the RCI (Relative Concentration Index), as described in (45). Positive scores indicate the transcripts are more likely to be found in the cytoplasm, and negative scores the nucleus. The dashed grey line indicates the mean RCI for all TE-free transcripts. Transcripts were allocated to a category if they contained one or more indicated TE-type. Data is from GSE143496 (74). (**N**) RBP binding eCLIP-seq data in hESCs for four RBPs: DDX6, FUS, ILF2 and DCP1B. The heatmap shows the percentage of transcripts containing a RBP binding peak in coding or noncoding transcripts with or without any copy of the indicated TE anywhere in the transcript. Data is from GSE112782 (42).

TE-derived sequences are found in the untranslated regions (UTRs) of coding transcripts (9), and contribute to lncRNAs (17,18). Additionally, deepCAGE data, which sequences only the 5′ ends of transcripts, has revealed pluripotent-specific TSSs that start inside TEs and contain part of the TE sequence (8,69). We searched for TE sequences in our assembled transcriptome using nhmmer (70), which uses hidden Markov models (HMMs) to detect sequence patterns inside DNA/RNA sequences. We used the Dfam collection of HMMs as input for nhmmer. Dfam HMMs are annotated collections of TE consensus sequences, grouped into families and TE types (41). The combination of nhmmer and Dfam allows the accurate detection of both full-length and fragmentary TEs in RNA. We removed non-primate TE families from the Dfam database, and then searched the assembled transcript sequences with nhmmer and Dfam and identified 37493 out of 101479 (37%) transcripts that contained at least one TE-derived sequence (Supplementary Table S4). For coding transcripts, about 22% (13427 out of 60575) of the matching transcripts contained a TE fragment, which was similar to the proportion of GENCODE coding transcripts (21%) (Figure 3A). However, 45% (5339 out of 11725) of variant coding transcripts contained a TE fragment (Figure 3A). There was a small number of novel coding transcripts (464), of which 139 (34%) were predicted to contain TE-derived sequences. However, as the number of novel coding transcripts (464 out of 7734 novel transcripts in total, 6%) is close to the expected false positive rate (5%) to distinguish coding from noncoding RNAs, we did not explore these further, except for LR supported coding transcripts (Supplementary Table S3). Surprisingly, hPSC-enriched transcripts were less likely than hPSC-nonspecific or hPSC-depleted transcripts to contain TE sequences (Figure 3B). This effect was not unique to the variant transcripts, as the same pattern was observed for transcripts matching GENCODE (Figure 3C). This is unexpected, as TEs are thought to be more actively expressed due to the relaxed chromatin in hPSCs (71). Overall, TE sequence fragments are mainly found in variant transcripts, but our data suggest that enrichment of TEs in coding transcripts is not a specific feature of hPSCs.

We next explored if there were differences between TE-containing and TE-free transcripts. We looked at several transcript properties, including total transcript length, intron length, exon length and the number of exons (Supplementary Figure S4A). Overall, noncoding transcripts were shorter than coding transcripts, with shorter introns and fewer exons, but the exon lengths were similar. As expected, the presence of TE sequences in transcripts did not correlate with the average number of exons. However, transcripts containing TE sequences tended to be longer, due to increased intron and exon lengths, and this was true for both coding and noncoding transcripts (Supplementary Figure S4A). This suggests that TE sequences are not leading to an increase in the numbers of exons, but they are correlated with increased transcript length of both the primary transcript and mature RNA.

TEs have nonrandom distributions in the genome (72). This is caused by several processes, including bias in insertion site preference, and evolutionary selection against deleterious insertions. TEs can also contain promoters that drive the initiation of transcription to create a hybrid first exon (8). These and other TE properties will bias the positions of TE fragments inside transcripts. We analyzed the positions of TE sequences within the mRNAs to determine if there is a bias towards the UTRs or the CDSs. We used the CDS from either the GENCODE reference, or using the longest ORF, and then measured the frequency of TE sequences in the UTRs or ORF/CDSs of coding transcripts, which revealed that TEs were enriched in the UTRs, particularly in the 3′UTRs, and depleted in the CDS (Figure 3D).

We next asked whether the frequency of TE sequences inside mRNAs varied by TE family. LINE:L1, LINE:L2 and SINE:Alu elements were specifically enriched in both UTRs, and particularly in the 3′UTRs (Figure 3E, and Supplementary Figure S4B, C). LINEs and SINEs were depleted in the CDS, compared to the UTRs (Figure 3E), and most LINE:L1 TEs were biased to the UTRs (Figure 3E). For example, LINE:L1:L1M2_orf2 was strongly biased to the 3′UTR, and LINE:L1:L1HS_5end was specifically enriched in the 5′UTR (Figure 3F, and Supplementary Figure S4D). Intriguingly, the L1HS family of LINEs are active and capable of retrotransposition in human cells (73), and their presence in the 5′UTRs of genes suggests regulation of fragmentary L1HS in hPSCs. In a pattern similar to LINEs, several SINE family members were present near the 3′ ends of transcripts (Figure 3E, and Supplementary Figure S4B, C). Fragments of ERV-family TEs were mainly biased to the 3′UTRs, except for the HERVH/LTR7-family of TEs that was enriched in the 5′UTR (Figure 3F and Supplementary Figure S4B). Previous analysis of deepCAGE data showed that HERVH/LTR7 acts as an hPSC-specific TSS (8), hence LTR7 fragments are likely to be found in the 5′UTR, which is what we observed (Figure 3F and Supplementary Figure S4E). We also observed transcripts with HERVH fragments interspersed throughout, including in the ORF and 3′UTR (Figure 3F, and Supplementary Figure S4F). These results show that, overall, TE sequences tend to be biased to the 3′UTRs, but some families of TE were found in the 5′UTR and the CDS.

We next looked at how the presence of TE fragments affected steady-state RNA levels. There was no significant difference between the average RNA levels of TE-containing and TE-free transcripts (Figure 3G). To explore the possibility that TEs are more or less tolerated in different parts of the transcript, we tested whether the location of a TE fragment within a coding transcript correlated with its steady-state level. Transcripts with a TE in the 5′UTR or CDS had significantly lower mean levels than the mean of TE-free transcripts. However, those transcripts with a TE fragment inside the 3′UTR were present at significantly (though modestly) higher levels compared to TE-free transcripts and transcripts with TEs inside the 5′UTR or CDS (Figure 3H). This was surprising, as a previous study indicated that TEs inside the 3′UTRs correlated with lower levels of RNA (69). One possible explanation for the discrepancy is that different TE-types have different effects. Indeed, dividing transcripts by the TE-type and location of the TE in a transcript indicated that almost all TE-containing transcripts had lower levels of RNA (Figure 3I). The exceptions were the DNA:hAT-Charlie, SVA, and SINE:Alu families, for example, MER117 and AluJr-containing transcripts had higher levels than TE-free transcripts, but only if the TE was in the 3′UTR (Figure 3J). Conversely, transcripts with HERVHs or L1 LINEs had significantly lower RNA levels (compared to TE-free transcripts) no matter the location of the TE sequence inside the mRNAs (Figure 3I and K). To explore the impact of TEs on different isoforms of the same transcript, we measured the fold-change of isoforms containing a TE fragment, versus isoforms of the same transcript that were TE-free. Most TE-containing isoforms of a transcript were present at significantly lower levels (Figure 3L), suggesting that, overall, TEs are deleterious to coding transcript accumulation.

As we have shown that TE sequences inside coding transcripts are associated with changes in the steady-state levels, we next sought to explore the mechanisms behind how TEs affect mRNAs. One way to modulate RNA function is to alter its subcellular distribution. We reanalyzed hPSC subcellular RNA-seq data for the cytoplasmic and nuclear fractions (74), and calculated the RCI (Relative Concentration Index) that describes the ratio of cytoplasmic reads over nuclear reads to give an overall score for subcellular distribution. TE-free noncoding transcripts tended to be found in the nucleus, and the TE-containing noncoding transcripts remained there (Figure 3M). Conversely, TE-free coding transcripts had a higher RCI and were more likely to be in the cytoplasm, but transcripts containing any family of TE had a lower RCI and were more likely to be in the nucleus (Figure 3M). This differential subcellular distribution suggests that the nuclear export machinery can recognize the difference between coding and noncoding transcripts or that some transcripts do not persist for a long enough time to be transported to the cytoplasm. Mechanistically, transcripts containing TEs could be recognized by RBPs that discriminate between coding, noncoding and TE-containing transcripts. To explore this idea, we reanalyzed eCLIP-seq (Enhanced crosslinking and immunoprecipitation) data in hPSCs for four RBPs: DDX6, FUS, ILF2, and DCP1B (42). DCP1B is a factor implicated in RNA decay (75), FUS and ILF2 are involved in splicing (76,77), and DDX6 has been implicated in several processes, including splicing, RNA decay, translation efficiency, and cellular differentiation (42,78). These RBPs showed two patterns of binding to TE-free transcripts: DDX6 was biased towards binding to coding transcripts, compared to noncoding, whilst FUS, ILF2, and DCP1B were equally bound to coding or noncoding (Figure 3N). For transcripts with a TE sequence, DDX6 binding was not correlated with the presence of a TE, but FUS, ILF2 and DCP1B were all more likely to be bound to a TE-containing transcript (Figure 3N). FUS was particularly interesting as it was almost entirely absent from TE-free transcripts and could only be detected bound to TE-containing transcripts. These results agree with previous observations in cancer cells that RBPs are specifically recruited to TE-containing transcripts (79). Overall, TE sequences in coding transcripts were correlated with reduced RNA levels, were more likely to be found in the nucleus, and had increased binding by RBPs.

### The incorporation of TE fragments affects the proteome of pluripotent cells

**Figure 4.**
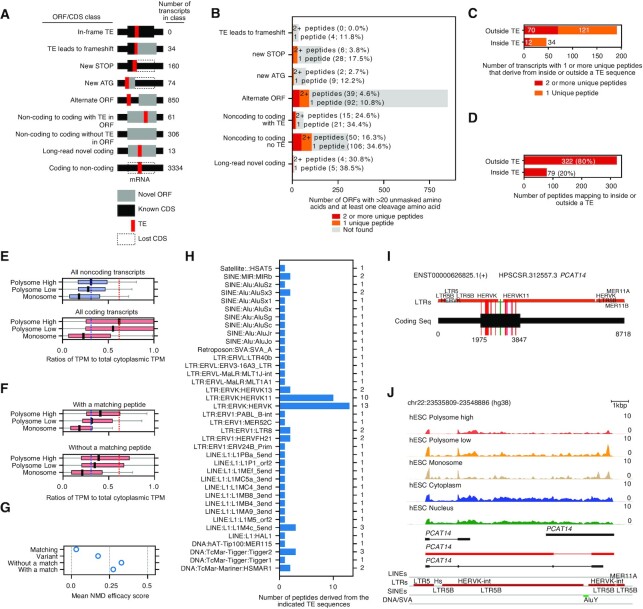
TEs disrupt coding sequences and in limited instances code for peptides. (**A**) Schematic of the number transcripts with the indicated effect of TE sequences on ORFs/CDSs. Thin black bars indicate the full length of the transcript, and thick black bars indicate the GENCODE annotated CDS or the predicted longest ORF. Red indicates the location of the TE sequence. Grey indicates a novel ORF due to a TE sequence, dotted lines indicate where the GENCODE annotated CDS would be if the TE was not present. Transcripts were assigned to a category in priority from top to bottom until they fulfilled the criteria for that category. (**B**) The number of novel ORFs/CDSs that have 2+ (red), 1+ (orange) or none (grey) MS peptides in hPSC data (HipSci MS dataset). The ORFs are divided into the same classes as in panel A. Any parts of the coding sequence that had a BLAST hit in the GENCODE protein dataset was masked, and only ORFs with at least 20 unmasked amino acids, and at least 1 lysine or arginine were kept. (**C**) The number of transcripts from any category in panel A or B that had a unique peptide derived from inside (overlapped with any TE sequence) or outside the TE sequence. (**D**) Number of peptides that map to any transcript, and overlap any part of a TE (inside TE) or only map to TE-free parts of the transcript (outside TE). (**E**) TrIP-seq data showing enrichment of noncoding (top) and coding (bottom) RNAs in the indicated polysome or monosome fractions. Boxplots show the ratios of TPMs for the polysome high (4+ ribosomes), low (2–4 ribosomes) or monosome fractions, versus cytoplasmic RNA. The dashed lines indicate the median of coding (red) or noncoding (blue) transcripts in the polysome high fraction. Data is from GSE100007 (83). (**F**) Box plots showing the distributions of the ratios of polysome/monosome against the cytoplasmic fraction for transcripts with an MS match (top) or without (bottom). The dashed lines indicate the median of all coding (red) or noncoding (blue) transcripts in the polysome high fraction (as in panel E). (**G**) Plot showing the mean efficacy of NMD for: all GENCODE-matching, predicted coding variants, and transcripts with a detectable peptide or without. NMD efficacy was measured using the decision tree NMDetective-B (86). (**H**) Numbers of unique peptides that map to the indicated TE subtype. A peptide was considered overlapping a TE if any amino acid codon overlapped a TE sequence. (**I**) Domain map for *PCAT14*, a variant transcript enriched in hPSCs. The black thin bar represents the full-length transcript and the thick black bar the location of the coding sequence. The location and type of LTR TEs is indicated on the horizontal line. The red/green vertical bars indicate the location of peptides that map inside a TE (red), or outside a TE (green). (**J**) Genome view (hg38 assembly) of the *PCAT14* locus, showing read pileup from polysome high, low, monosome fractions and total cytoplasmic and nuclear RNA. Three noncoding *PCAT14* isoforms are indicated in black, and the predicted coding and HERVK-containing isoform is marked in red. The final track shows LTRs and SINEs from the RepeatMask annotation track.

We next analyzed TE sequences inside the CDSs of coding transcripts, to explore if TEs can directly contribute to the proteome. We divided the transcripts into classes based upon the effect of the TE sequence on the CDSs or predicted ORF (Figure 4A). We did not detect any in-frame TE sequences inside CDSs and found only 34 transcripts with a frameshift in the CDS due to the presence of TE-derived sequences (Figure 4A). The largest class was the conversion of a canonical (GENCODE) coding transcript to a predicted noncoding transcript (3334 transcripts) due to the presence of 1 or more TE sequence fragments. There were some GENCODE annotated noncoding transcripts either with (61 transcripts), or without (306 transcripts) a TE that were predicted to be coding, and several TEs led to a premature STOP (160 transcripts) or to the introduction of a new ATG (74 transcripts) that would alter a pre-existing CDS. However, the most common effect a TE caused was to disrupt an existing CDS and lead to another ORF becoming the best-predicted CDS (850 transcripts) (Figure 4A). Overall, TE sequences inside coding transcripts were disruptive for coding potential.

Discrimination between coding and noncoding transcripts is not always clear-cut and is a challenging problem (52,80,81). For predicted coding transcripts, whilst they contain an ORF, they may not yield a peptide, as they may not be translated or may be targeted for nonsense-mediated decay (NMD). Additionally, some TE types, particularly the SINE/Alu and SVA TEs, have coding-like sequence signatures (e.g. long ORFs and specific frequencies of k-mers) but do not encode proteins (82). To estimate how many of the predicted ORFs are translated we searched hPSC mass spectrometry data for peptides produced by the predicted ORFs. We first stringently filtered the ORF peptide sequences to make sure we were detecting novel peptides. From the peptide sequences we deleted any regions that had a >90% BLAST hit against any GENCODE protein, removed sequences shorter than 20 amino acids and retained only those that had at least one Trypsin/Lys-C cleavage site. These criteria meant that we would only detect peptides derived from the TE-encoded portions of a protein, or entirely from TE sequences. We then used the HipSci hESC/iPSC proteomics LC-MS/MS data (53–55) to search for peptides. Overall, we detected at least one peptide match for 237 out of 1536 transcripts (15.4%) and two or more peptides from 82 transcripts (5.4%) (Figure 4B and Supplementary Table S5), indicating that at least some TE-containing transcripts produce peptides. The majority of peptides detected were encoded by sequences outside of the TE portion of the transcript, and only 20% had a codon that overlapped with a TE nucleotide (Figure 4C and D), which matches the percentage (19%) of TE nucleotides in the transcripts. These data indicate that only a minority of predicted TE-containing/modified CDSs produce detectable peptides.

To provide further evidence for translation, we reanalyzed hESC TrIP-seq (Transcript Isoforms in Polysome-sequencing) which sequences RNAs from monosome, low (2–4 ribosomes) and high (4+ ribosome) polysome fractions based on their elution from a sucrose gradient (83). As expected, coding transcripts were enriched in the polysome high and low fractions, whilst noncoding transcripts were depleted (Figure 4E). Intriguingly, transcripts with a detectable peptide were enriched in the polysome high fraction, albeit not as high as coding transcripts (Figure 4F). Curiously, transcripts without a detectable peptide were also enriched in the polysome high fraction, albeit at a lower level (Figure 4F). It was curious that the transcripts without a detectable MS peptide were enriched in the polysome fractions (at least, more than noncoding transcripts). One possibility is that these transcripts are recognized as abnormal and degraded by NMD (84). The full rules governing NMD are not completely understood (85), but a simple decision tree model, NMDetective-B (86), can explain ∼68% of the NMD variation. We applied NMDetective-B to our transcripts and measured the mean predicted probability of NMD for several transcript classes (Figure 4G). Transcripts matching GENCODE had a low mean probability of NMD (0.03), variant transcripts were higher (0.17). Transcripts containing a TE without an MS match were further increased (0.32), whilst those with an MS hit were slightly reduced (0.27). These results suggest that NMD can help explain why some of the transcripts can be detected in the polysome-bound RNA fraction, but do not produce detectable peptides.

We next explored the peptides that were originating from TE sequences. There was at least one example of a TE-derived peptide from all major families of TE, including SINE, LINE, LTR, retroposons and DNA transposons. Nine peptides were derived from SINE:Alu family TEs (Figure 4H). This was unexpected, as SINEs do not encode proteins and rely on LINE encoded proteins for retrotransposition. Using BLAST (against the human non-redundant protein set) to search the SINE-derived peptides did not produce any significant hits. There were also peptides from LINEs, although again BLAST did not report any significant hits, suggesting they are frameshifted fragments of LINEs, rather than in frame LINE proteins. The largest number of peptides were derived from HERVK LTRs originating mainly from four transcripts (Figure 4H, I and Supplementary Figure S5A), but as many as 10 transcripts may be contributing peptides (Supplementary Table S5). The peptides mapped to the viral proteins *gag*, *pol* and *env* but not *pro*, of a putative progenitor HERVK (87) (23 unique peptides in total) (Supplementary Figure S5B). These results are consistent with reports that HERVK RNAs and proteins are expressed in hPSCs (88). Interestingly, 14 unique HERVK peptides mapped to a single variant hPSC-enriched isoform of the *PCAT14* gene (prostate cancer-associated transcript 14) that has hitherto been considered noncoding. We observed 14 unique HERVK peptides derived from *PCAT14*, and the predicted ORFs in *PCAT14* code for a near-complete product of *gag* and fragments of *pol* (Figure 4I, Supplementary Figure S6A–C and Supplementary Table S5). The *PCAT14* transcript was found in the cytoplasmic fraction and bound by polysomes (Figure 4J), which supports translation. For other ERVs, we did not observe any peptides from HERVH, in agreement with a previous report that the HERVH ORFs are not functional (89). In summary, these data indicate that TE sequences are mainly disruptive for coding sequences, and even though they retain a coding-like signature and are predicted to be coding, only a small minority of TE-containing transcripts can produce detectable protein.

### TEs orchestrate the lncRNA complement of hPSCs, and are correlated with reduced RNA half-life

**Figure 5.**
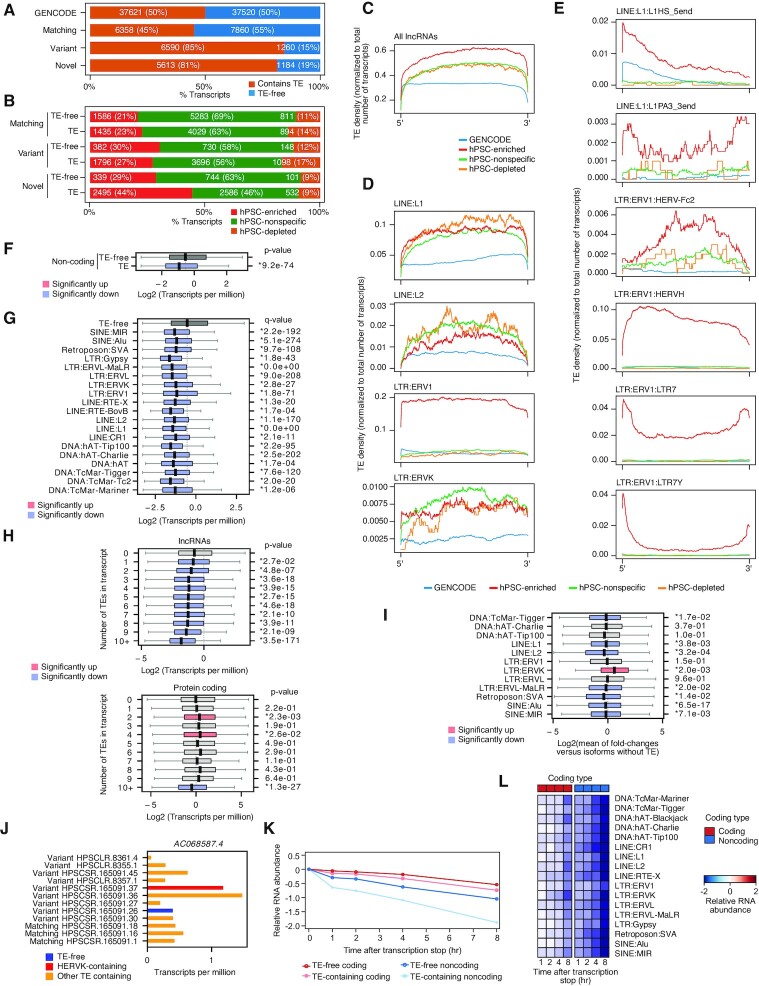
Widespread type-specific presence of TEs in hPSC lncRNAs. (**A**) Percentage of noncoding transcripts in the indicated class that contain a TE-derived sequence or are TE-free, for GENCODE transcripts, hPSC-matching, variant and novel. (**B**) Barplot showing the frequency of transcripts with or without a TE, divided into matching, variant, or novel, and their hPSC expression class, enriched, nonspecific, or depleted in hPSCs. (**C**) Line plots showing the TE frequency within noncoding transcripts scaled to a uniform length, and normalized to the number of transcripts for the indicated classes of hPSC expression or all GENCODE transcripts. (**D**) Line plots of the TE frequencies for LINEs, L1 and L2, and the LTRs, ERV1 and ERVK. (**E**) Line plots of the TE frequencies in noncoding transcripts for selected LINEs (L1HS_5end, L1PA3_3end), and LTRs (HERV-Fc2, HERVH, LTR7 and LTR7Y). (**F**) Box plot for the RNA levels of noncoding transcripts with 1 or more TEs, or TE-free. p-values are from a two-sided Welch's t-test for TE-containing transcripts versus all TE-free transcripts. Blue colored boxes represent significantly down, grey no significant change. (**G**) Box pots showing the RNA levels of transcripts containing the indicated TE types, versus all TE-free transcripts. q-value is from a two-sided Welch's t-test with Bonferroni-Hochberg multiple testing correction. (**H**) Box plots showing the RNA levels of noncoding or coding RNAs with 0, or 1 or more TE sequences. *p*-value is from a two-sided Welch's t-test, versus all TE-free transcripts. (**I**) Transcript isoforms of the same gene were merged by their gene name, and the fold-change was calculated for the TE-containing isoforms versus the TE-free isoforms. The boxplots show the spread of fold-changes for all genes that have at least one TE-free isoform and at least 1 TE of the indicated type. p-values are from a two-sided one-sample *t*-test. (**J**) TPM values for isoforms of the noncoding transcript *AC068587.4*. The HERVK-containing isoform is in red, other TE-containing isoforms are in orange and the TE-free isoform is in blue. (**K**) RNA-seq time course data after transcriptional arrest with actinomycin D, showing coding and noncoding transcripts. RNA abundance is relative to 0 hr (untreated). Transcripts were divided into coding and noncoding and TE-containing and TE-free. Data is from GSE156671. (**L**) RNA-seq data from a transcriptional arrest time course (as in panel K), showing transcripts containing the indicated TE types. Transcripts containing more than one type of TE would be allocated to multiple classes.

Previous reports show that TE sequences constitute a major part of lncRNAs (17,18). Consistently, we observed a large number of noncoding transcripts containing at least 1 TE sequence (18561 out of 28685, 65%), less than the 83% reported in a smaller set of lncRNAs (18). Of the noncoding transcripts matching GENCODE, 45% (6358 out of 14218) contained a TE, and the variant and novel transcripts were more likely to contain a TE (Figure 5A). Interestingly, and in contrast to coding transcripts, novel and variant noncoding transcripts containing a TE were more likely to be enriched in hPSCs (Figure 5B). As TEs inside coding transcripts showed family and position-specific bias in their location inside mRNAs, we next looked at the position and types of TE inside lncRNA sequences. TEs in lncRNAs were found anywhere from the 5′ to the 3′ end with a slight bias towards the 3′ end (Figure 5C). At the TE family level, LINEs were enriched in lncRNAs, such as LINE:L1:L1PA3_3end and LINE:L1:L1Hs_5end (Figure 5D, E and Supplementary Figure S7A). In addition to LINEs, the SINEs, DNA, SVA and Satellite-type repeats were also enriched (Supplementary Figure S7A-E). Overall, the LTRs had the most complex patterns (Figure 5D and E), and especially the ERV1 and ERVK families of TEs (Supplementary Figure S7F and G). LTR7, the LTR for HERVH, can function as a hPSC-specific TSS (8). However, unlike coding transcripts, LTR7 not was limited to just the 5′ end of the transcript and LTR7 and HERVH sequences were found throughout noncoding transcripts (Figure 5E). There were complex patterns of other LTR sequences; for example, HERVFH21 and HERVH48 were generally found in the middle of transcripts, and not at the 5′ or 3′ ends (Supplementary Figure S7G). Overall, these results indicate that hPSC noncoding transcripts are enriched for TEs, which are distributed throughout the lengths of noncoding transcripts and TEs have family-specific biases in their location within a transcript sequence.

We next looked at the relationship between TE sequences and lncRNA steady-state levels. In contrast to coding transcripts, lncRNAs containing TEs had a significantly lower level compared to lncRNAs that were TE-free (Figure 5F). This effect was not TE-type specific, and all TE types had significantly lower RNA levels, compared to TE-free transcripts (Figure 5G). There was also a dose-dependent effect, as noncoding RNAs with increasing numbers of TE fragments had decreased transcript levels, an effect not seen in coding transcripts (Figure 5H). TE-containing transcripts have lower overall RNA levels compared to all TE-free transcripts; however, this effect may not apply to different TE families in transcript isoforms of the same gene. To explore this, we grouped isoforms of the same gene and measured the fold change of the TE-containing isoforms versus the TE-free isoform. Most TE-types were downregulated, but some HERVK-family containing transcript isoforms were upregulated (Figure 5I). For example, in the noncoding transcript *AC068587.4*, the HERVK containing isoform had the second highest expression (Figure 5J). This was the exception though, and overall, TE fragments inside transcripts were correlated with lower RNA levels in a dose-dependent manner.

To explore the mechanism controlling the decreased levels of noncoding RNAs, we reanalyzed an RNA-seq timecourse from cells treated with actinomycin D to stop transcription (GSE156671). We noticed that TE-containing coding transcripts had only slightly reduced the RNA half-life compared to TE-free coding transcripts (Figure 5K). Noncoding TE-containing transcripts however had a considerably reduced RNA half-life compared to TE-free noncoding or coding transcripts (Figure 5K). This effect was present in all transcripts containing any TE-type sequence, with the notable exception of transcripts containing LTR:ERV1 fragments, which showed no substantive difference between coding and noncoding half-lives (Figure 5L). Potentially this change in RNA half-life is driven by increased RBP binding to TE-containing noncoding transcripts (Figure 3N). These results indicate that, in direct contrast to coding transcripts, the presence of TE sequences inside noncoding RNAs correlated with lower steady-state levels, an effect at least partly attributable decreased RNA half-life.

### TE sequences inside noncoding RNAs are generally not conserved compared to the flanking sequences

**Figure 6.**
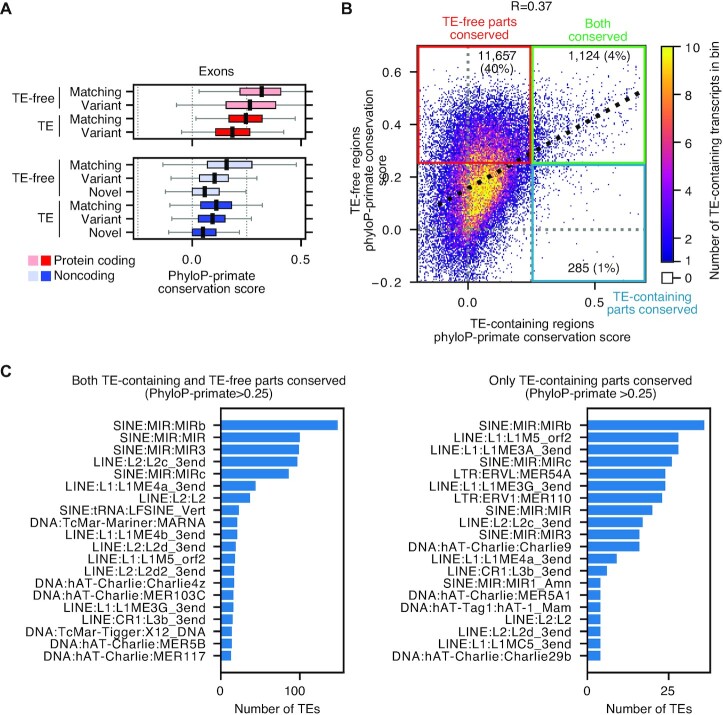
Conservation of TE regions inside lncRNAs. (**A**) Boxplots showing the mean and spread of primate conservation scores for exons of coding or noncoding transcripts, either containing a TE sequence or TE-free. The transcripts are further subdivided by whether they have a perfect internal exon match to a GENCODE transcript (matching), overlap any exon in a GENCODE transcript (variant) or do not overlap any exon of a GENCODE transcript (novel). (**B**) 2D histogram showing the phyloP-primate conservation scores for averages of the TE-containing versus the TE-free parts of the transcript. The x-axis shows the phyloP-primate conservation score for the TE-containing parts of the transcript, and the y-axis shows the conservation score for the TE-free parts of the transcript. An arbitrary cut-off of 0.25 was considered as moderately conserved. The colored quadrants indicate noncoding transcripts that have: primate conservation (phyloP-primate > 0.25) in both the TE-containing and TE-free parts of the transcript sequence (green box), conservation only in the TE-free parts of the sequence (red box) or conservation only within the TE-containing parts (blue box). The number and percentage of total noncoding transcripts (37,492 transcripts in total) is indicated in each quadrant. (**C**) The number of TE domains in transcripts with evidence of evolutionary conservation (PhyloP-primate > 0.25). The number of TE subtypes that are conserved were counted. The left bar chart shows those transcripts where both the TE-containing and TE-free parts of the transcript show evolutionary conservation. The right bar chart shows those transcripts where only the TE-containing parts of the transcript are conserved.

TE sequences have complex patterns of evolutionary conservation, and they can show signs of evolving under purifying selection, which implies function (90). To explore the evolutionary conservation of TEs in expressed transcripts we took advantage of the base pair resolution conservation scores calculated by phyloP, which estimates the rate of nucleotide substitution compared to random nucleotide changes (46). Positive scores indicate conservation, whilst negative scores imply accelerated mutation. We used the primate conservation track from the UCSC genome browser, as many of the TEs we are analyzing are primate-specific. Overall, noncoding transcripts were poorly conserved compared to coding transcripts, in agreement with previous observations (91). Additionally, TE-containing transcripts had further reduced average conservation scores compared to TE-free transcripts, for both coding and noncoding transcripts (Figure 6A).

Noncoding transcripts are poorly conserved, but we wondered if this was caused by differences in conservation of TEs versus the TE-free parts of the noncoding transcript. Potentially, noncoding transcripts may accumulate new TE DNA insertions that disrupt the overall conservation of the transcript, but not its function. Conversely, inactive TE sequences may accumulate mutations faster than the functional non-TE parts of the transcript, or vice versa if the TEs are acting as functional domains in noncoding transcripts (25). We measured the base pair conservation of the TE-containing parts of transcripts and the TE-free parts of transcripts. Plotting the two scores against each other resulted in a modest correlation (*R* = 0.37), and most (15619 out of 28685, 54%) lncRNAs showed no primate evolutionary conservation for either the TE-containing or TE-free parts of the lncRNA (Figure 6B). A subset (11657 out of 28685, 40%) showed conservation only in the TE-free parts of the lncRNA. Very few lncRNAs (285 out of 28685, 1%) were conserved only in the TE sequences. Finally, a small fraction of lncRNAs had moderate conservation for both the TE-containing and TE-free sequences (1124 out of 28685, 4%). Amongst the types of TE sequences that were conserved, LTRs were rare, however, several SINE and LINE types were conserved (Figure 6C). Particularly prominent were the SINE:MIRs, an ancient mammalian-specific TE family with surprisingly high levels of evolutionary conservation (92), that have been shown to function as transcriptional enhancers (93). Our data shows that they are also conserved sequences in noncoding RNAs (Figure 6C). Overall, most lncRNAs are poorly conserved, but about a third of lncRNAs are conserved in the TE-free parts of their sequences. Conversely, TE-containing parts of lncRNA sequences are poorly conserved, except for MIR elements and some LINEs.

### Single cell RNA-seq expression of lincRNAs, heterogeneity in TE splicing

We took advantage of our hPSC-specific transcript assembly to look at the distribution of TE-containing transcripts in single cells. Analysis of TE expression in other systems has revealed an association between biological phenomena and TE RNAs, for example, a class of MERVLs in mouse cells are associated with totipotent properties (6). Analysis of TEs in hPSCs has not been performed, and most sc-RNA-seq analysis is gene-based, rather than transcript-based and does not take into account the TE content of transcripts. Single cell RNA-seq (sc-RNA-seq) techniques can measure the expression of genes in individual cells. However, identifying transcripts can be ambiguous as the reads produced by the most common sc-RNA-seq techniques are heavily biased to the 3′ ends. Consequently, we reduced our transcript assembly to those with unique non-overlapping strand-specific 3′ ends and collapsed overlapping transcript 3′ ends to a single transcript. This resulted in a total set of 88520 transcripts (87% of the total superset of 101479 transcripts). We generated two sc-RNA-seq datasets, one from WIBR3 hESCs and another from S0730/c11 iPSCs (34), supplemented with five samples of WTC line iPSCs from E-MTAB-6687 (56), and two UCLA1 line hESC samples from GSE140021 (57). We aligned the reads to the hg38 genome assembly and annotated the reads to our 3′ end transcript database. On average 50–70% of the reads could be aligned to our 3′ end transcriptome (in comparison, for the same samples, 60–70% of reads align to full-length GENCODE transcripts). The majority of the 3′ ends show strand-specific read pileups (Supplementary Figure S8A) indicating that our transcript assembly has accurate 3′ ends. This approach should include alternatively polyadenylated transcripts, as we take a similar strategy to Shulman and Elkon (94), but use our custom transcript assembly rather than the GENCODE assembly. After filtering and normalization, we retrieved 30001 cells, and 35400 transcript ends. In bulk RNA-seq samples, noncoding RNAs are expressed at lower levels than coding transcripts (51,68) (Figure 1G). However, there are suggestions that this may be an artifact, as potentially coding and noncoding transcripts could have similar RNA levels, but the noncoding transcripts could be expressed in a smaller number of cells. Our data agrees with this suggestion, as the mean expression level was similar for both coding and noncoding transcripts (∼3.5 UMI tags), but coding transcripts were detected on average in 6% of cells whilst noncoding transcripts were detected in only 2.2% of cells (Supplementary Figure S8B and C). This suggests noncoding transcripts are expressed at similar levels to coding transcripts, but in fewer cells, hence bulk RNA-seq would underestimate lncRNA expression levels. This analysis comes with some caveats though, as we assume that coding and noncoding transcripts are equally detectable and quantifiable in single cells, and the drop-out rate is equal between coding and noncoding transcripts.

**Figure 7.**
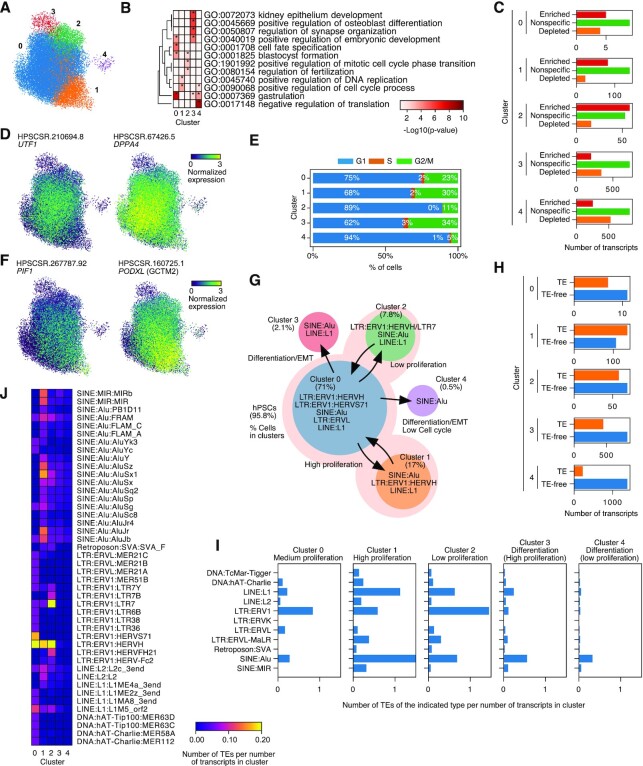
Single cell expression of hPSC shows subpopulations of cells expressing distinct types of TE-containing transcripts. (**A**) UMAP (uniform manifold and projection) plot showing the clustering of the hPSC sc-RNA-seqdata using the Leiden algorithm (resolution = 1.0). (**B**) Gene ontology (GO) analysis of the genes significantly associated with the indicated clusters. (**C**) Numbers of transcripts specific to each cluster that are hPSC-enriched, nonspecific or depleted. (**D**) UMAP plots colored by the normalized expression level of two pluripotency genes, *UTF1* and *DPPA4*. (**E**) Estimates of cell cycle phase of the indicated UMAP clusters based on the normalized expression of cell cycle-related transcripts. (**F**) UMAP plot colored by expression of the G2/M-associated genes *PIF1*, and *PODXL* (also known as GCTM2). (**G**) Schematic indicating the relationship between the hPSC cell sub-populations. The % of cells in each cluster is indicated, and the major TE types expressed are indicated. The label on each arrow is the suggested biological process for each subpopulation. Subpopulations of cells with high levels of pluripotency transcripts are shaded in salmon (Clusters 0, 1 and 2). (**H**) The number of transcripts specific to each cluster that are TE-containing or TE-free. (**I**) Bar chart showing the number of TE-types per the number of transcripts specific to the indicatedcluster s. (**J**) Heatmap indicating the number of TE subtypes contained in transcripts specific to the indicated cluster. The number of each TE subtype was counted and normalized to the total number of transcripts specific to each cluster.

Before looking at the TE complement of single hPSCs, we first identified the subpopulations of cells in hPSC cultures. Projection of the cells into a UMAP (Uniform Manifold and Approximation Projection) plot showed no clear separation between hESCs and iPSC samples, and no bias in the biological replicate samples (Supplementary Figure S8D and E), indicating the sample quality is good. We detected five major clusters of cells (Figure 7A). To identify the characteristics of each cluster we performed GO (gene ontology) analysis for differentially regulated transcripts specific to each cluster. GO analysis of transcripts specific to each cluster suggested clusters 0 and 2 represented pluripotent cells, as represented by the enriched terms ‘blastocyst formation’, and gastrulation’ (Figure 7B). Clusters 0, 1 and 2 also had higher numbers of hPSC-enriched transcripts (Figure 7C), and specific expression of the pluripotency marker genes, *UTF1*, *DPPA4*, *SOX2*, *LIN28A*, and *NODAL* (Figure 7D and Supplementary Figure S8F).

GO analysis suggested that the transcripts specific to cluster 1 were enriched for cell cycle-related genes, as represented by the GO terms ‘positive regulation of DNA replication’ and ‘positive regulation of cell cycle’ (Figure 7B). The cell cycle stage for each cell can be estimated based on the expression of cell cycle-stage-specific genes (61). This analysis suggested a spectrum of cell cycle activity: Cluster 1 had the highest estimated level of G2/M cells (30%), cluster 0 was intermediate, with 23% and cluster 2 had only 11% of G2/M cells (Figure 7E). This is exemplified by the specific expression of marker genes correlated with high proliferation: *TOP2A*, *MALAT1*, *PIF1* (Figure 7F and Supplementary Figure S8G). This matches a previous study that identified a subpopulation of rapidly proliferating hPSCs (56), and a second study that identified high proliferating cells based on the proliferation markers: *EPCAM* and *PODXL* (GCTM2) (95) (Figure 7F and Supplementary Figure S8G, H).

Our data contained two novel clusters of cells containing only a few cells, cluster 3 (2.1%) and cluster 4 (0.5%). GO analysis of genes specific to cluster 3 suggested that these cells were spontaneously differentiating, as indicated by the overrepresentation of terms related to kidney epithelium, osteoblast differentiation, and synapse formation (Figure 7B). Clusters 3 and 4 also had decreased numbers of hPSC-enriched transcripts and had higher numbers of hPSC-depleted transcripts and pluripotent marker genes had reduced expression, also suggesting differentiation (Figure 7C and D). There was no clear bias towards a specific differentiation lineage, based on GO analysis and transcripts specific to clusters 3 and 4, but there was a strong shift in epithelial and mesenchymal genes. The epithelial genes *CDH1* and *EPCAM* were downregulated and the mesenchymal genes *CDH2* and *VIM* were up-regulated (Supplementary Figure S8H and I). This is reminiscent of the epithelial-mesenchymal transition (EMT) in the early stage of hepatocyte differentiation (96). The main difference between clusters 3 and 4 was in cell cycle activity, cluster 3 had high predicted numbers of G2/M cells (34%), whilst cluster 4 cells were predicted to have low G2/M activity (5%) (Figure 7E). These data show that hPSC cultures are heterogenous, and contain five major subpopulations of cells (Figure 7G): Cluster 0: The bulk population of pluripotent hPSCs. Cluster 1: rapidly proliferating hPSCs. Custer 2: slowly proliferating hPSCs. Cluster 3: Spontaneously differentiating cells with high proliferation. Cluster 4: Spontaneously differentiating cells with low proliferation.

In addition to gene expression heterogeneity in single cells, there is evidence that TEs are heterogeneously expressed and mark subpopulations of cells (58). However, the TE complement in sc-RNA-seq has only been analyzed by merging all genomic TE copies to produce a single expression score for each TE (58), or by using short reads to guide transcript assembly to then measure TE enrichment in specific RNAs (97). As we use unique 3′ ends we can associate the 3′ end with the corresponding full-length transcript from our hPSC-specific assembly, and so measure the TE content of the expressed transcripts in each cell. The number of transcripts with TEs was higher in clusters 0, 1 and 2, and was reduced in clusters 3 and 4 (Figure 7H). We measured the frequency of TE types inside the transcripts specific to each cluster and observed a unique TE-type ‘fingerprint’ for each cluster (Figure 7I). Clusters 0, 1 and 2 were enriched for LTR:ERV1-containing transcripts, which was mainly due to the presence of HERVH and LTR7-containing transcripts (Figure 7J and Supplementary Figure S9A and B). Differentiating cells (cluster 3, 4) had lower levels of TE-containing transcripts, LTR:ERV1-containing transcripts were nearly absent, and only showed some enrichment of SINE:Alu-containing transcripts (Figure 7I and Supplementary Figure S9C and D). HERVH and LTR7-containing transcripts were nearly absent from cells in clusters 3 and 4 (Figure 7I). Overall, HERVH, LTR7 and LINE:L1-containing transcripts were restricted to the main population of hPSCs in clusters 0, 1 and 2. hPSCs undergoing differentiation did not express HERVH, LTR7, or LINE:L1-containing transcripts, and only had SINE:Alu-containing transcripts. This single cell data indicates that subpopulations of cells in an hPSC culture have distinct sets of transcripts containing different sets of TEs.

## DISCUSSION

TEs constitute a major proportion of the DNA sequence of the human genome (1). The vast majority of TEs are fragmentary and incapable of transposition due to mutations, but TEs persist in the genome and have been implicated in a wide range of activities (9). The non-functional TEs can be expressed as parts of RNAs, including parts of existing transcripts, or can form novel transcripts. However, the analysis of TEs is challenging due to their repetitive nature, and assembling full length transcripts that preserve TE genomic and transcriptome context is challenging. Consequently, the full contribution of TE sequences to the transcriptome has not been thoroughly analyzed. Here, using a combination of short and long read RNA-seq data, we show that TE sequences are an integral part of the hPSC-transcriptome, and are correlated with changes in RNA levels, half-life, subcellular distribution and RBP binding profiles.

The binding of RBPs to TEs is a potential mechanism to regulate TE-containing transcripts. In our reanalysis of the RBP data from Di Stefano (42), the different modes of RBP binding to transcripts was striking. DDX6 was bound to RNAs independent of any TE sequences in the transcript, however DCP1B, ILF2 and FUS were preferentially recruited to TE-containing transcripts. This matches other RBPs in different cellular contexts, for example, STAU1 can bind to SINE-containing transcripts (98), and MATR3/PTBP1 can bind to transcripts containing LINEs (99). A recent large-scale analysis of RBP-bound RNAs revealed the widespread biding of RBPs to TEs, particularly on SINE and LINEs (100). However, the functional consequences of RBPs binding to TE sequences in RNAs has only been explored in a few instances. As TEs harbor binding sites for RBPs, and the human genome may contain as many as ∼2900 RBPs (101), of which the majority have no known function or RNA binding profile, there is a lot to explore. Accurate cell type-specific transcript assemblies will be an important contribution to understand the profile of RBP binding to TEs.

The presence of TE sequences within coding transcripts reduced their ability to function as mRNAs compared to the coding potential of the same transcript that lacked TE sequences. Specifically, TEs introduced frameshift mutations, premature STOP codons, and altered the coding sequences to produce new peptides and disrupt CDS signatures (k-mer and longest ORFs). One class of TE-containing coding transcripts that produced detectable peptides were those that included sequences derived from fragments of or intact HERVK viral proteins. We detected peptides from a near-complete viral *env* protein. Interestingly, HERVK *env* proteins were specifically detected in the neurons of amyotrophic lateral sclerosis (ALS) patients, and transgenic mice overexpressing HERVK *env* suffered from neurodegeneration caused by toxicity to the *env* protein (102). It is intriguing that hPSCs appear to have no ill effects from the presence of HERVK *env* peptides. The expression of TEs has also been observed in several cancers (103), where TEs promote and form part of pluripotency transcripts that can act as oncogenes in cancer cells. For example, a SINE:AluJb acts as a promoter and the first exon of the pluripotency gene *LIN28B* and LTR:MLT1J performs a similar function for *SALL4* (3,104,105). These TEs convert the pluripotent genes into oncogenes, and the deletion of the TE from the genome eliminated their expression (3). Intriguingly, in our hPSC-specific transcript assembly, we did not observe SINE:AluJb in any *LIN28B* transcript or LTR:MLT1J in a *SALL4* transcript (Supplementary Figure S10A–C). This suggests that these are cancer-specific transcripts, and implies that there is a normal set of TEs in transcripts (8), and a distinct set that is associated with disease. Indeed, hPSC-specific HERVs were not associated with the expression of pluripotency-related transcripts in human cancers (106), showing that the expression of hPSC-specific TEs is not a feature of cancer.

There were substantial differences between the TE-free and TE-containing coding and noncoding RNAs, particularly, steady-state levels, RNA half-life, RBP binding, and subcellular distribution of RNAs. Coding transcripts with TE sequences in the 5′UTR and CDS tended to have lower RNA levels, whilst TEs seem to be tolerated in the 3′UTRs, and correlated with higher RNA levels. For noncoding transcripts, the presence of TEs was correlated with lower RNA levels, and the higher the number of TEs in the transcript, the lower the level of RNA. These effects have been hinted at before (69), but we show here that the effects are TE-type specific, and whilst most TE types are correlated with reduced RNA, some TEs are associated with increased RNA levels, particularly ERVs in noncoding transcripts and SINEs in the 3′UTRs of coding transcripts. Our finding that different TE families had distinct positional preferences inside coding and noncoding transcripts provides further evidence that the effects of TEs on RNAs are complex and TE-type specific.

Overall, our analysis demonstrates that TE sequences are incorporated into the RNAs of hPSCs and have a greater impact than previously appreciated. Utilizing ultra-deep short read sequence data and guided by long read RNA-seq we assembled transcribed TEs in their transcriptomic context and explored how TEs can impact steady-state RNA levels, half-life, subcellular distribution and RBP binding patterns. Our data suggests that TEs have important roles in regulating RNA metabolism, and that TEs are a major component of the normal transcriptome of hPSCs.

## DATA AVAILABILITY

The long read RNA-seq was deposited in the Sequence Read Archive (SRA) with the accession number: PRJNA631047, and the sc-RNA-seq data with the accession number: PRJNA631808.

## Supplementary Material

gkab710_Supplemental_Files

## References

[1] Hutchins A.P., Pei D Transposable elements at the center of the crossroads between embryogenesis, embryonic stem cells, reprogramming, and long non-coding RNAs. Sci. Bull. 2015; 60:1722–1733. 10.1007/s11434-015-0905-x PMC4624819 26543668

[2] Jurka J., Kapitonov V.V., Kohany O., Jurka M.V. Repetitive sequences in complex genomes: structure and evolution. Annu. Rev. Genomics Hum. Genet. 2007; 8:241–259. 17506661 10.1146/annurev.genom.8.080706.092416

[3] Jang H.S., Shah N.M., Du A.Y., Dailey Z.Z., Pehrsson E.C., Godoy P.M., Zhang D., Li D., Xing X., Kim S. et al. Transposable elements drive widespread expression of oncogenes in human cancers. Nat. Genet. 2019; 51:611–617. 30926969 10.1038/s41588-019-0373-3 PMC6443099

[4] Clayton E.A., Rishishwar L., Huang T.C., Gulati S., Ban D., McDonald J.F., Jordan I.K. An atlas of transposable element-derived alternative splicing in cancer. Philos. Trans. R. Soc. Lond. B Biol. Sci. 2020; 375:20190342. 32075558 10.1098/rstb.2019.0342 PMC7061986

[5] Wang J., Xie G., Singh M., Ghanbarian A.T., Rasko T., Szvetnik A., Cai H., Besser D., Prigione A., Fuchs N.V. et al. Primate-specific endogenous retrovirus-driven transcription defines naive-like stem cells. Nature. 2014; 516:405–409. 25317556 10.1038/nature13804

[6] Macfarlan T.S., Gifford W.D., Driscoll S., Lettieri K., Rowe H.M., Bonanomi D., Firth A., Singer O., Trono D., Pfaff S.L. Embryonic stem cell potency fluctuates with endogenous retrovirus activity. Nature. 2012; 487:57–63. 22722858 10.1038/nature11244 PMC3395470

[7] Theunissen T.W., Friedli M., He Y., Planet E., O’Neil R.C., Markoulaki S., Pontis J., Wang H., Iouranova A., Imbeault M. et al. Molecular criteria for defining the naive human pluripotent state. Cell Stem Cell. 2016; 19:502–515. 27424783 10.1016/j.stem.2016.06.011 PMC5065525

[8] Fort A., Hashimoto K., Yamada D., Salimullah M., Keya C.A., Saxena A., Bonetti A., Voineagu I., Bertin N., Kratz A. et al. Deep transcriptome profiling of mammalian stem cells supports a regulatory role for retrotransposons in pluripotency maintenance. Nat. Genet. 2014; 46:558–566. 24777452 10.1038/ng.2965

[9] Bourque G., Burns K.H., Gehring M., Gorbunova V., Seluanov A., Hammell M., Imbeault M., Izsvak Z., Levin H.L., Macfarlan T.S. et al. Ten things you should know about transposable elements. Genome Biol. 2018; 19:199. 30454069 10.1186/s13059-018-1577-z PMC6240941

[10] Kunarso G., Chia N.Y., Jeyakani J., Hwang C., Lu X., Chan Y.S., Ng H.H., Bourque G. Transposable elements have rewired the core regulatory network of human embryonic stem cells. Nat. Genet. 2010; 42:631–634. 20526341 10.1038/ng.600

[11] Feng S., Jacobsen S.E., Reik W. Epigenetic reprogramming in plant and animal development. Science. 2010; 330:622–627. 21030646 10.1126/science.1190614 PMC2989926

[12] Jonsson M.E., Ludvik Brattas P., Gustafsson C., Petri R., Yudovich D., Pircs K., Verschuere S., Madsen S., Hansson J., Larsson J. et al. Activation of neuronal genes via LINE-1 elements upon global DNA demethylation in human neural progenitors. Nat. Commun. 2019; 10:3182. 31320637 10.1038/s41467-019-11150-8 PMC6639357

[13] Bulut-Karslioglu A., Macrae T.A., Oses-Prieto J.A., Covarrubias S., Percharde M., Ku G., Diaz A., McManus M.T., Burlingame A.L., Ramalho-Santos M. The transcriptionally permissive chromatin state of embryonic stem cells is acutely tuned to translational output. Cell Stem Cell. 2018; 22:369–383. 29499153 10.1016/j.stem.2018.02.004 PMC5836508

[14] Sun L., Fu X., Ma G., Hutchins A.P. Chromatin and epigenetic rearrangements in embryonic stem cell fate transitions. Front. Cell Dev. Biol. 2021; 9:637309. 33681220 10.3389/fcell.2021.637309 PMC7930395

[15] Goke J., Lu X., Chan Y.S., Ng H.H., Ly L.H., Sachs F., Szczerbinska I. Dynamic transcription of distinct classes of endogenous retroviral elements marks specific populations of early human embryonic cells. Cell Stem Cell. 2015; 16:135–141. 25658370 10.1016/j.stem.2015.01.005

[16] Grow E.J., Flynn R.A., Chavez S.L., Bayless N.L., Wossidlo M., Wesche D.J., Martin L., Ware C.B., Blish C.A., Chang H.Y. et al. Intrinsic retroviral reactivation in human preimplantation embryos and pluripotent cells. Nature. 2015; 522:221–225. 25896322 10.1038/nature14308 PMC4503379

[17] Kapusta A., Kronenberg Z., Lynch V.J., Zhuo X., Ramsay L., Bourque G., Yandell M., Feschotte C. Transposable elements are major contributors to the origin, diversification, and regulation of vertebrate long noncoding RNAs. PLos Genet. 2013; 9:e1003470. 23637635 10.1371/journal.pgen.1003470 PMC3636048

[18] Kelley D., Rinn J. Transposable elements reveal a stem cell-specific class of long noncoding RNAs. Genome Biol. 2012; 13:R107. 23181609 10.1186/gb-2012-13-11-r107 PMC3580499

[19] Lev-Maor G., Ram O., Kim E., Sela N., Goren A., Levanon E.Y., Ast G. Intronic Alus influence alternative splicing. PLos Genet. 2008; 4:e1000204. 18818740 10.1371/journal.pgen.1000204 PMC2533698

[20] Naville M., Warren I.A., Haftek-Terreau Z., Chalopin D., Brunet F., Levin P., Galiana D., Volff J.N. Not so bad after all: retroviruses and long terminal repeat retrotransposons as a source of new genes in vertebrates. Clin. Microbiol. Infect. 2016; 22:312–323. 26899828 10.1016/j.cmi.2016.02.001

[21] Goff L.A., Rinn J.L. Linking RNA biology to lncRNAs. Genome Res. 2015; 25:1456–1465. 26430155 10.1101/gr.191122.115 PMC4579330

[22] Lu J.Y., Shao W., Chang L., Yin Y., Li T., Zhang H., Hong Y., Percharde M., Guo L., Wu Z. et al. Genomic repeats categorize genes with distinct functions for orchestrated regulation. Cell Rep. 2020; 30:3296–3311. 32160538 10.1016/j.celrep.2020.02.048 PMC7195444

[23] Wapinski O., Chang H.Y. Long noncoding RNAs and human disease. Trends Cell Biol. 2011; 21:354–361. 21550244 10.1016/j.tcb.2011.04.001

[24] Carlevaro-Fita J., Lanzos A., Feuerbach L., Hong C., Mas-Ponte D., Pedersen J.S., Drivers P. Functional Interpretation, G. Functional Interpretation, G. Johnson R., Consortium P. Cancer LncRNA Census reveals evidence for deep functional conservation of long noncoding RNAs in tumorigenesis. Commun Biol. 2020; 3:56. 32024996 10.1038/s42003-019-0741-7 PMC7002399

[25] Johnson R., Guigo R. The RIDL hypothesis: transposable elements as functional domains of long noncoding RNAs. RNA. 2014; 20:959–976. 24850885 10.1261/rna.044560.114 PMC4114693

[26] Chishima T., Iwakiri J., Hamada M. Identification of transposable elements contributing to tissue-specific expression of long non-coding RNAs. Genes (Basel). 2018; 9:23. 10.3390/genes9010023 PMC5793176 29315213

[27] Morillon A., Gautheret D Bridging the gap between reference and real transcriptomes. Genome Biol. 2019; 20:112. 31159855 10.1186/s13059-019-1710-7 PMC6545731

[28] You B.H., Yoon S.H., Nam J.W. High-confidence coding and noncoding transcriptome maps. Genome Res. 2017; 27:1050–1062. 28396519 10.1101/gr.214288.116 PMC5453319

[29] Ma L., Cao J., Liu L., Du Q., Li Z., Zou D., Bajic V.B., Zhang Z. LncBook: a curated knowledgebase of human long non-coding RNAs. Nucleic Acids Res. 2019; 47:D128–D134. 30329098 10.1093/nar/gky960 PMC6323930

[30] Schumann G.G., Fuchs N.V., Tristan-Ramos P., Sebe A., Ivics Z., Heras S.R. The impact of transposable element activity on therapeutically relevant human stem cells. Mob DNA. 2019; 10:9. 30899334 10.1186/s13100-019-0151-x PMC6408843

[31] Babarinde I.A., Li Y., Hutchins A.P. Computational methods for mapping, assembly and quantification for coding and non-coding transcripts. Comput Struct Biotechnol J. 2019; 17:628–637. 31193391 10.1016/j.csbj.2019.04.012 PMC6526290

[32] Steijger T., Abril J.F., Engstrom P.G., Kokocinski F., Consortium R., Hubbard T.J., Guigo R., Harrow J., Bertone P. Assessment of transcript reconstruction methods for RNA-seq. Nat. Methods. 2013; 10:1177–1184. 24185837 10.1038/nmeth.2714 PMC3851240

[33] Lagarde J., Uszczynska-Ratajczak B., Carbonell S., Perez-Lluch S., Abad A., Davis C., Gingeras T.R., Frankish A., Harrow J., Guigo R. et al. High-throughput annotation of full-length long noncoding RNAs with capture long-read sequencing. Nat. Genet. 2017; 49:1731–1740. 29106417 10.1038/ng.3988 PMC5709232

[34] Zhou T., Benda C., Duzinger S., Huang Y., Li X., Li Y., Guo X., Cao G., Chen S., Hao L. et al. Generation of induced pluripotent stem cells from urine. J. Am. Soc. Nephrol. 2011; 22:1221–1228. 21636641 10.1681/ASN.2011010106 PMC3137570

[35] Kim D., Paggi J.M., Park C., Bennett C., Salzberg S.L. Graph-based genome alignment and genotyping with HISAT2 and HISAT-genotype. Nat. Biotechnol. 2019; 37:907–915. 31375807 10.1038/s41587-019-0201-4 PMC7605509

[36] Li H., Handsaker B., Wysoker A., Fennell T., Ruan J., Homer N., Marth G., Abecasis G., Durbin R. Genome Project Data Processing, S The sequence alignment/map format and SAMtools. Bioinformatics. 2009; 25:2078–2079. 19505943 10.1093/bioinformatics/btp352 PMC2723002

[37] Pertea M., Pertea G.M., Antonescu C.M., Chang T.C., Mendell J.T., Salzberg S.L. StringTie enables improved reconstruction of a transcriptome from RNA-seq reads. Nat. Biotechnol. 2015; 33:290–295. 25690850 10.1038/nbt.3122 PMC4643835

[38] Gordon S.P., Tseng E., Salamov A., Zhang J., Meng X., Zhao Z., Kang D., Underwood J., Grigoriev I.V., Figueroa M. et al. Widespread polycistronic transcripts in fungi revealed by single-molecule mRNA sequencing. PLoS One. 2015; 10:e0132628. 26177194 10.1371/journal.pone.0132628 PMC4503453

[39] Barnett D.W., Garrison E.K., Quinlan A.R., Stromberg M.P., Marth G.T. BamTools: a C++ API and toolkit for analyzing and managing BAM files. Bioinformatics. 2011; 27:1691–1692. 21493652 10.1093/bioinformatics/btr174 PMC3106182

[40] Wu T.D., Watanabe C.K. GMAP: a genomic mapping and alignment program for mRNA and EST sequences. Bioinformatics. 2005; 21:1859–1875. 15728110 10.1093/bioinformatics/bti310

[41] Hubley R., Finn R.D., Clements J., Eddy S.R., Jones T.A., Bao W., Smit A.F., Wheeler T.J. The Dfam database of repetitive DNA families. Nucleic. Acids. Res. 2016; 44:D81–D89. 26612867 10.1093/nar/gkv1272 PMC4702899

[42] Di Stefano B., Luo E.C., Haggerty C., Aigner S., Charlton J., Brumbaugh J., Ji F., Rabano Jimenez I., Clowers K.J., Huebner A.J. et al. The RNA helicase DDX6 controls cellular plasticity by modulating P-body homeostasis. Cell Stem Cell. 2019; 25:622–638. 31588046 10.1016/j.stem.2019.08.018 PMC7247364

[43] Dobin A., Davis C.A., Schlesinger F., Drenkow J., Zaleski C., Jha S., Batut P., Chaisson M., Gingeras T.R. STAR: ultrafast universal RNA-seq aligner. Bioinformatics. 2013; 29:15–21. 23104886 10.1093/bioinformatics/bts635 PMC3530905

[44] Zhang Y., Liu T., Meyer C.A., Eeckhoute J., Johnson D.S., Bernstein B.E., Nusbaum C., Myers R.M., Brown M., Li W. et al. Model-based analysis of ChIP-Seq (MACS). Genome Biol. 2008; 9:R137. 18798982 10.1186/gb-2008-9-9-r137 PMC2592715

[45] Mas-Ponte D., Carlevaro-Fita J., Palumbo E., Hermoso Pulido T., Guigo R., Johnson R. LncATLAS database for subcellular localization of long noncoding RNAs. RNA. 2017; 23:1080–1087. 28386015 10.1261/rna.060814.117 PMC5473142

[46] Pollard K.S., Hubisz M.J., Rosenbloom K.R., Siepel A. Detection of nonneutral substitution rates on mammalian phylogenies. Genome Res. 2010; 20:110–121. 19858363 10.1101/gr.097857.109 PMC2798823

[47] Fantom Consortium and the Riken PMI and CLST Forrest A.R., Kawaji H., Rehli M., Baillie J.K., de Hoon M.J., Haberle V., Lassmann T., Kulakovskiy I.V., Lizio M. et al. A promoter-level mammalian expression atlas. Nature. 2014; 507:462–470. 24670764 10.1038/nature13182 PMC4529748

[48] Ramirez F., Dundar F., Diehl S., Gruning B.A., Manke T. deepTools: a flexible platform for exploring deep-sequencing data. Nucleic. Acids. Res. 2014; 42:W187–W191. 24799436 10.1093/nar/gku365 PMC4086134

[49] Cheng L.C., Zheng D., Baljinnyam E., Sun F., Ogami K., Yeung P.L., Hoque M., Lu C.W., Manley J.L., Tian B. Widespread transcript shortening through alternative polyadenylation in secretory cell differentiation. Nat. Commun. 2020; 11:3182. 32576858 10.1038/s41467-020-16959-2 PMC7311474

[50] Li H., Durbin R. Fast and accurate short read alignment with Burrows-Wheeler transform. Bioinformatics. 2009; 25:1754–1760. 19451168 10.1093/bioinformatics/btp324 PMC2705234

[51] Derrien T., Johnson R., Bussotti G., Tanzer A., Djebali S., Tilgner H., Guernec G., Martin D., Merkel A., Knowles D.G. et al. The GENCODE v7 catalog of human long noncoding RNAs: analysis of their gene structure, evolution, and expression. Genome Res. 2012; 22:1775–1789. 22955988 10.1101/gr.132159.111 PMC3431493

[52] Wucher V., Legeai F., Hedan B., Rizk G., Lagoutte L., Leeb T., Jagannathan V., Cadieu E., David A., Lohi H. et al. FEELnc: a tool for long non-coding RNA annotation and its application to the dog transcriptome. Nucleic. Acids. Res. 2017; 45:e57. 28053114 10.1093/nar/gkw1306 PMC5416892

[53] Kim S., Pevzner P.A. MS-GF+ makes progress towards a universal database search tool for proteomics. Nat. Commun. 2014; 5:5277. 25358478 10.1038/ncomms6277 PMC5036525

[54] Kilpinen H., Goncalves A., Leha A., Afzal V., Alasoo K., Ashford S., Bala S., Bensaddek D., Casale F.P., Culley O.J. et al. Common genetic variation drives molecular heterogeneity in human iPSCs. Nature. 2017; 546:370–375. 28489815 10.1038/nature22403 PMC5524171

[55] Mirauta B.A., Seaton D.D., Bensaddek D., Brenes A., Bonder M.J., Kilpinen H., HipSci C., Agu C.A., Alderton A., Danecek P. et al. Population-scale proteome variation in human induced pluripotent stem cells. Elife. 2020; 9:e57390. 32773033 10.7554/eLife.57390 PMC7447446

[56] Nguyen Q.H., Lukowski S.W., Chiu H.S., Senabouth A., Bruxner T.J.C., Christ A.N., Palpant N.J., Powell J.E. Single-cell RNA-seq of human induced pluripotent stem cells reveals cellular heterogeneity and cell state transitions between subpopulations. Genome Res. 2018; 28:1053–1066. 29752298 10.1101/gr.223925.117 PMC6028138

[57] Chen D., Sun N., Hou L., Kim R., Faith J., Aslanyan M., Tao Y., Zheng Y., Fu J., Liu W. et al. Human primordial germ cells are specified from lineage-primed progenitors. Cell Rep. 2019; 29:4568–4582. 31875561 10.1016/j.celrep.2019.11.083 PMC6939677

[58] He J., Babarinde I.A., Sun L., Xu S., Chen R., Shi J., Wei Y., Li Y., Ma G., Zhuang Q. et al. Identifying transposable element expression dynamics and heterogeneity during development at the single-cell level with a processing pipeline scTE. Nat. Commun. 2021; 12:1456. 33674594 10.1038/s41467-021-21808-x PMC7935913

[59] Wolf F.A., Angerer P., Theis F.J. SCANPY: large-scale single-cell gene expression data analysis. Genome Biol. 2018; 19:15. 29409532 10.1186/s13059-017-1382-0 PMC5802054

[60] Lun A.T., McCarthy D.J., Marioni J.C. A step-by-step workflow for low-level analysis of single-cell RNA-seq data with Bioconductor. F1000Res. 2016; 5:2122. 27909575 10.12688/f1000research.9501.1 PMC5112579

[61] Macosko E.Z., Basu A., Satija R., Nemesh J., Shekhar K., Goldman M., Tirosh I., Bialas A.R., Kamitaki N., Martersteck E.M. et al. Highly parallel genome-wide expression profiling of individual cells using nanoliter droplets. Cell. 2015; 161:1202–1214. 26000488 10.1016/j.cell.2015.05.002 PMC4481139

[62] Hutchins A.P., Jauch R., Dyla M., Miranda-Saavedra D glbase: a framework for combining, analyzing and displaying heterogeneous genomic and high-throughput sequencing data. Cell Regener. 2014; 3:1. 10.1186/2045-9769-3-1 PMC4230833 25408880

[63] Dobin A., Gingeras T.R. Mapping RNA-seq reads with STAR. Curr. Protoc. Bioinformatics. 2015; 51:11.14.11–11.14.19. 26334920 10.1002/0471250953.bi1114s51 PMC4631051

[64] Toker L., Feng M., Pavlidis P. Whose sample is it anyway? Widespread misannotation of samples in transcriptomics studies. F1000Res. 2016; 5:2103. 27746907 10.12688/f1000research.9471.1 PMC5034794

[65] Hutchins A.P., Yang Z., Li Y., He F., Fu X., Wang X., Li D., Liu K., He J., Wang Y. et al. Models of global gene expression define major domains of cell type and tissue identity. Nucleic Acids Res. 2017; 45:2354–2367. 28426095 10.1093/nar/gkx054 PMC5389706

[66] Ozsolak F., Milos P.M. RNA sequencing: advances, challenges and opportunities. Nat. Rev. Genet. 2011; 12:87–98. 21191423 10.1038/nrg2934 PMC3031867

[67] Wang X., You X., Langer J.D., Hou J., Rupprecht F., Vlatkovic I., Quedenau C., Tushev G., Epstein I., Schaefke B. et al. Full-length transcriptome reconstruction reveals a large diversity of RNA and protein isoforms in rat hippocampus. Nat. Commun. 2019; 10:5009. 31676752 10.1038/s41467-019-13037-0 PMC6825209

[68] Necsulea A., Soumillon M., Warnefors M., Liechti A., Daish T., Zeller U., Baker J.C., Grutzner F., Kaessmann H. The evolution of lncRNA repertoires and expression patterns in tetrapods. Nature. 2014; 505:635–640. 24463510 10.1038/nature12943

[69] Faulkner G.J., Kimura Y., Daub C.O., Wani S., Plessy C., Irvine K.M., Schroder K., Cloonan N., Steptoe A.L., Lassmann T. et al. The regulated retrotransposon transcriptome of mammalian cells. Nat. Genet. 2009; 41:563–571. 19377475 10.1038/ng.368

[70] Wheeler T.J., Eddy S.R. nhmmer: DNA homology search with profile HMMs. Bioinformatics. 2013; 29:2487–2489. 23842809 10.1093/bioinformatics/btt403 PMC3777106

[71] Schlesinger S., Meshorer E. Open chromatin, epigenetic plasticity, and nuclear organization in pluripotency. Dev. Cell. 2019; 48:135–150. 30695696 10.1016/j.devcel.2019.01.003

[72] Medstrand P., van de Lagemaat L.N., Mager D.L. Retroelement distributions in the human genome: variations associated with age and proximity to genes. Genome Res. 2002; 12:1483–1495. 12368240 10.1101/gr.388902 PMC187529

[73] Beck C.R., Collier P., Macfarlane C., Malig M., Kidd J.M., Eichler E.E., Badge R.M., Moran J.V. LINE-1 retrotransposition activity in human genomes. Cell. 2010; 141:1159–1170. 20602998 10.1016/j.cell.2010.05.021 PMC3013285

[74] Guo C.J., Ma X.K., Xing Y.H., Zheng C.C., Xu Y.F., Shan L., Zhang J., Wang S., Wang Y., Carmichael G.G. et al. Distinct processing of lncRNAs contributes to non-conserved functions in stem cells. Cell. 2020; 181:621–636. 32259487 10.1016/j.cell.2020.03.006

[75] Cougot N., Babajko S., Seraphin B. Cytoplasmic foci are sites of mRNA decay in human cells. J. Cell Biol. 2004; 165:31–40. 15067023 10.1083/jcb.200309008 PMC2172085

[76] Marchesini M., Ogoti Y., Fiorini E., Aktas Samur A., Nezi L., D’Anca M., Storti P., Samur M.K., Ganan-Gomez I., Fulciniti M.T. et al. ILF2 is a regulator of RNA splicing and DNA damage response in 1q21-amplified multiple myeloma. Cancer Cell. 2017; 32:88–100. 28669490 10.1016/j.ccell.2017.05.011 PMC5593798

[77] Humphrey J., Birsa N., Milioto C., McLaughlin M., Ule A.M., Robaldo D., Eberle A.B., Krauchi R., Bentham M., Brown A.L. et al. FUS ALS-causative mutations impair FUS autoregulation and splicing factor networks through intron retention. Nucleic Acids Res. 2020; 48:6889–6905. 32479602 10.1093/nar/gkaa410 PMC7337901

[78] Wang Y., Arribas-Layton M., Chen Y., Lykke-Andersen J., Sen G.L. DDX6 orchestrates mammalian progenitor function through the mRNA degradation and translation pathways. Mol. Cell. 2015; 60:118–130. 26412305 10.1016/j.molcel.2015.08.014 PMC4592480

[79] Kelley D.R., Hendrickson D.G., Tenen D., Rinn J.L. Transposable elements modulate human RNA abundance and splicing via specific RNA-protein interactions. Genome Biol. 2014; 15:537. 25572935 10.1186/s13059-014-0537-5 PMC4272801

[80] Camargo A.P., Sourkov V., Pereira G.A.G., Carazzolle M.F. RNAsamba: neural network-based assessment of the protein-coding potential of RNA sequences. NAR Genom. Bioinform. 2020; 2:lqz024. 33575571 10.1093/nargab/lqz024 PMC7671399

[81] Abascal F., Juan D., Jungreis I., Kellis M., Martinez L., Rigau M., Rodriguez J.M., Vazquez J., Tress M.L. Loose ends: almost one in five human genes still have unresolved coding status. Nucleic Acids Res. 2018; 46:7070–7084. 29982784 10.1093/nar/gky587 PMC6101605

[82] Jungreis I., Tress M.L., Mudge J., Sisu C., Hunt T., Johnson R., Uszczynska-Ratajczak B., Lagarde J., Wright J., Muir P. et al. Nearly all new protein-coding predictions in the CHESS database are not protein-coding. 2018; bioRxiv doi:02 July 2018, preprint: not peer reviewed 10.1101/360602.

[83] Blair J.D., Hockemeyer D., Doudna J.A., Bateup H.S., Floor S.N. Widespread Translational Remodeling during Human Neuronal Differentiation. Cell Rep. 2017; 21:2005–2016. 29141229 10.1016/j.celrep.2017.10.095 PMC5759054

[84] Yi Z., Sanjeev M., Singh G. The branched nature of the nonsense-mediated mRNA decay pathway. Trends Genet. 2021; 37:143–159. 33008628 10.1016/j.tig.2020.08.010 PMC7854845

[85] Supek F., Lehner B., Lindeboom R.G.H. To NMD or Not To NMD: nonsense-mediated mRNA decay in cancer and other genetic diseases. Trends Genet. 2021; 37:657–668. 33277042 10.1016/j.tig.2020.11.002

[86] Lindeboom R.G.H., Vermeulen M., Lehner B., Supek F. The impact of nonsense-mediated mRNA decay on genetic disease, gene editing and cancer immunotherapy. Nat. Genet. 2019; 51:1645–1651. 31659324 10.1038/s41588-019-0517-5 PMC6858879

[87] Dewannieux M., Harper F., Richaud A., Letzelter C., Ribet D., Pierron G., Heidmann T. Identification of an infectious progenitor for the multiple-copy HERV-K human endogenous retroelements. Genome Res. 2006; 16:1548–1556. 17077319 10.1101/gr.5565706 PMC1665638

[88] Fuchs N.V., Loewer S., Daley G.Q., Izsvak Z., Lower J., Lower R. Human endogenous retrovirus K (HML-2) RNA and protein expression is a marker for human embryonic and induced pluripotent stem cells. Retrovirology. 2013; 10:115. 24156636 10.1186/1742-4690-10-115 PMC3819666

[89] Santoni F.A., Guerra J., Luban J. HERV-H RNA is abundant in human embryonic stem cells and a precise marker for pluripotency. Retrovirology. 2012; 9:111. 23253934 10.1186/1742-4690-9-111 PMC3558390

[90] Chuong E.B., Elde N.C., Feschotte C. Regulatory activities of transposable elements: from conflicts to benefits. Nat. Rev. Genet. 2017; 18:71–86. 27867194 10.1038/nrg.2016.139 PMC5498291

[91] Ponjavic J., Ponting C.P., Lunter G. Functionality or transcriptional noise? Evidence for selection within long noncoding RNAs. Genome Res. 2007; 17:556–565. 17387145 10.1101/gr.6036807 PMC1855172

[92] Silva J.C., Shabalina S.A., Harris D.G., Spouge J.L., Kondrashovi A.S. Conserved fragments of transposable elements in intergenic regions: evidence for widespread recruitment of MIR- and L2-derived sequences within the mouse and human genomes. Genet. Res. 2003; 82:1–18. 14621267 10.1017/s0016672303006268

[93] Jjingo D., Conley A.B., Wang J., Marino-Ramirez L., Lunyak V.V., Jordan I.K. Mammalian-wide interspersed repeat (MIR)-derived enhancers and the regulation of human gene expression. Mob DNA. 2014; 5:14. 25018785 10.1186/1759-8753-5-14 PMC4090950

[94] Shulman E.D., Elkon R. Cell-type-specific analysis of alternative polyadenylation using single-cell transcriptomics data. Nucleic Acids Res. 2019; 47:10027–10039. 31501864 10.1093/nar/gkz781 PMC6821429

[95] Lau K.X., Mason E.A., Kie J., De Souza D.P., Kloehn J., Tull D., McConville M.J., Keniry A., Beck T., Blewitt M.E. et al. Unique properties of a subset of human pluripotent stem cells with high capacity for self-renewal. Nat. Commun. 2020; 11:2420. 32415101 10.1038/s41467-020-16214-8 PMC7229198

[96] Li Q., Hutchins A.P., Chen Y., Li S., Shan Y., Liao B., Zheng D., Shi X., Li Y., Chan W.Y. et al. A sequential EMT-MET mechanism drives the differentiation of human embryonic stem cells towards hepatocytes. Nat. Commun. 2017; 8:15166. 28466868 10.1038/ncomms15166 PMC5418622

[97] Shao W., Wang T. Transcript assembly improves expression quantification of transposable elements in single-cell RNA-seq data. Genome Res. 2021; 31:88–100. 33355230 10.1101/gr.265173.120 PMC7849386

[98] Gong C., Maquat L.E. lncRNAs transactivate STAU1-mediated mRNA decay by duplexing with 3′ UTRs via Alu elements. Nature. 2011; 470:284–288. 21307942 10.1038/nature09701 PMC3073508

[99] Attig J., Agostini F., Gooding C., Chakrabarti A.M., Singh A., Haberman N., Zagalak J.A., Emmett W., Smith C.W.J., Luscombe N.M. et al. Heteromeric RNP assembly at LINEs controls lineage-specific RNA processing. Cell. 2018; 174:1067–1081. 30078707 10.1016/j.cell.2018.07.001 PMC6108849

[100] Van Nostrand E.L., Freese P., Pratt G.A., Wang X., Wei X., Xiao R., Blue S.M., Chen J.Y., Cody N.A.L., Dominguez D. et al. A large-scale binding and functional map of human RNA-binding proteins. Nature. 2020; 583:711–719. 32728246 10.1038/s41586-020-2077-3 PMC7410833

[101] Liao J.Y., Yang B., Zhang Y.C., Wang X.J., Ye Y., Peng J.W., Yang Z.Z., He J.H., Zhang Y., Hu K. et al. EuRBPDB: a comprehensive resource for annotation, functional and oncological investigation of eukaryotic RNA binding proteins (RBPs). Nucleic Acids Res. 2020; 48:D307–D313. 31598693 10.1093/nar/gkz823 PMC6943034

[102] Li W., Lee M.H., Henderson L., Tyagi R., Bachani M., Steiner J., Campanac E., Hoffman D.A., von Geldern G., Johnson K. et al. Human endogenous retrovirus-K contributes to motor neuron disease. Sci. Transl. Med. 2015; 7:307ra153. 10.1126/scitranslmed.aac8201 PMC6344353 26424568

[103] Burns K.H. Transposable elements in cancer. Nat. Rev. Cancer. 2017; 17:415–424. 28642606 10.1038/nrc.2017.35

[104] Yang J., Gao C., Chai L., Ma Y. A novel SALL4/OCT4 transcriptional feedback network for pluripotency of embryonic stem cells. PLoS One. 2010; 5:e10766. 20505821 10.1371/journal.pone.0010766 PMC2874005

[105] Yu J., Vodyanik M.A., Smuga-Otto K., Antosiewicz-Bourget J., Frane J.L., Tian S., Nie J., Jonsdottir G.A., Ruotti V., Stewart R. et al. Induced pluripotent stem cell lines derived from human somatic cells. Science. 2007; 318:1917–1920. 18029452 10.1126/science.1151526

[106] Zapatka M., Borozan I., Brewer D.S., Iskar M., Grundhoff A., Alawi M., Desai N., Sultmann H., Moch H., Pathogens P. et al. The landscape of viral associations in human cancers. Nat. Genet. 2020; 52:320–330. 32025001 10.1038/s41588-019-0558-9 PMC8076016

