# Supplementary material for: Transposable element sequence fragments incorporated into coding and noncoding transcripts modulate the transcriptome of human pluripotent stem cells
Source: Nucleic Acids Res. 2021 Aug 14;49(16):9132–53. doi: 10.1093/nar/gkab710 (PMC8450112; doi:10.1093/nar/gkab710)
Supplement: gkab710_Supplemental_Files [file gkab710_supplemental_files.zip › Hutchins_Manuscript_Supplement.pdf]

Transposable element sequence fragments incorporated into coding and noncoding transcripts modulate the transcriptome of human pluripotent stem cells

Isaac A. Babarinde[1,2], Gang Ma[1,2], Yuhao Li[1,2], Boping Deng[2,3], Zhiwei Luo[4,5], Hao Liu[4,5], Mazid Md. Abdul[4,5], Carl Ward[4,5], Minchun Chen[2], Xiuling Fu[1,2], Liyang Shi[1,2], Martha Duttlinger[2], Jiangping He[6], Li Sun[1,2], Wenjuan Li[4,5], Qiang Zhuang[2], Guoqing Tong[7], Jon Frampton[3], Jean-Baptiste Cazier[3,8], Jiekai Chen[5,6,9], Ralf Jauch[10], Miguel A. Esteban[4,5,11], Andrew P. Hutchins[1,2#]

Supplementary Information

Supplementary Table S1 – Primers used for transcript validation. The table containing primer sequences forward and reverse for the PCR experiments in Supplementary Figure 2A.

Supplementary Table S2 – Metadata for the publicly available data used in this study. The table contains four sub-tables describing the publicly available data, or data generated, in this study: Sub-table 1, All short read (SR_samples). Sub-table 2, All Long read (LR_samples). Sub-table 3, Somatic samples, the samples used in Figure 2B, and Sub-table 4, Other samples, including the eCLIP-seq, RNA half-life data, TrIP-seq ribosome fractions data and Subcellular localization data. Each sub-table contains the Short Read Archive (SRA) or European Nucleotide Archive (ENA) accession number for the data, the total number of reads in each sample, and the read length (in base pairs). The SR_samples sub-table also includes insert size estimates. The LR_table also includes columns for the cell type, and the minimum, maximum, mean and median lengths of the long reads.

Supplementary Table S3 – Metadata for the short and long read transcripts assembled in this study. The table contains metadata for each of the 101,479 transcripts assembled in this study. Metadata includes 21 columns: (1) The transcript ID, unique for each transcript in this study. (2) the hg38 genomic location and strand. (3) number of exons, (4) whether the transcript matches is variant or novel compared to GENCODE. (5, 6) the gene or transcript name if it is a matching or variant transcript, (7, 8) the Ensembl gene and transcript ID if it is matching or variant. (9) The expression bias in hPSCs (enriched,

nonspecific or depleted). (10) The Z-score (Figure 2A) for each transcript was used to determine enriched, nonbiased or depleted. (11) the coding or noncoding prediction from FEELnc. (12) Whether the transcript is supported by short reads (SR), Long reads (LR) or both (SR+LR). (13) Per base coverage from StringTie, i.e. the average number of reads for each nucleotide. (14) Transcripts per million (TPM). (15) Percentage match to GENCODE exon structure (not including the 5' and 3' ends). (16) DeepCAGE count. The number of deepCAGE tags within 200 base pairs of the 5' end of the transcript. (17) Transcript length, exons and introns. (18) Transcript length only using the exons. (19) Total length of the introns. (20) Percentage of the unspliced pre-RNA that is exonic. (21) percentage of the unspliced pre-RNA that is intronic.

Supplementary Table S4 – Summary of the 37,493 transcripts that contain one or more TE sequence fragments. The table contains 8 columns: (1) The transcript ID, unique for each transcript in this study. (2) Ensembl Gene ID for matching and variant transcripts. (3) Ensembl transcript ID for matching and variant transcripts. (4) Gene symbol for matching and variant transcripts. (5) coding or noncoding prediction from FEELnc. (6) hPSC expression status: enriched, depleted, or nonspecific (Figure 2A). (7) Transcripts per million (TPM) (8) TE structure. This is a compound field, that contains multiple possible entries, one for each TE found. Each TE entry has 4 keys: E (e-value reported by nhmmer), 'span', the start and stop in nucleotides where the TE appears in the spliced transcript. 'strand' the strand the TE sequence was discovered (This is always relative to the RNA strand, which is set to '+'. A TE in the negative strand would be a TE sequence that is reversed). 'TEtype': the TE type name from the Dfam HMM database.

Supplementary Table S5 – Peptides matches against the variant coding sequences. Summary of the 668 peptides found in Figure 4B. The table contains 12 columns. (1) The transcript ID, unique for each transcript in this study. (2) Ensembl transcript ID for matching and variant transcripts. (3) Gene symbol for matching and variant transcripts. (4) Transcript class, as described in Figure 4A. (5) q-value (False discovery Rate) reported by MS-GF+ from the target decoy approach. (6) the amino acid sequence

reported by MS-GF+, includes amino acid mass modifications. (7) The amino acid sequence, without mass modifications. (8) Does the amino acid sequence result from a TE sequence? If yes, the TE type the amino acid sequence originates from. (9, 10) The position of the amino acid sequence in the ORF. (11, 12) The position in the RNA nucleotides that the amino acid sequence originates from.

Supplementary Table S6 – Differentially expressed genes in the sc-RNA-seq clusters. This table has 5 sub tables, one for each cluster of cells in Figure 7A. Each sub-table contains 6 fields: (1) The transcript ID, unique for each transcript in this study. (2) The Log2(Fold-change) reported by scanpy. rank_genes_groups. (3) The q-value (Bonferroni-Hochberg corrected p-values). (4) Ensembl Gene ID for matching and variant transcripts. (5) Ensembl transcript ID for matching and variant transcripts. (6) Gene symbol for matching and variant transcripts.

Supplementary Methods

Code availability

The full code tree for the analysis presented in this paper can be found at: https://github.com/oaxiom/hesc_lincrna. The code is available under an MIT license. The code repository hosts data files that researchers may be interested in:

GTF file for the assembly	https://github.com/oaxiom/hesc_lincrna/blob/master/transcript_assembly/gtf/current_gtf.gtf.gz
Table of transcripts and TEs	https://github.com/oaxiom/hesc_lincrna/blob/master/te_discovery/te_transcripts/transcript_table_merged.mapped.tsv.gz
Transcript feature table	https://github.com/oaxiom/hesc_lincrna/blob/master/transcript_assembly/feature_table/assembly_hPSC_detailed.tsv.gz
Peptide mass spec results	https://github.com/oaxiom/hesc_lincrna/blob/master/massspec/results_gene.tsv.gz

Code for short and long read alignment and transcript assembly

#Preparing reference for HISAT2

```
$ extract_splice_sites.py Homo_sapiens.GRCh38.91.gtf >Homo_sapiens.GRCh38.91.ss
$ extract_exons.py Homo_sapiens.GRCh38.91.gtf >Homo_sapiens.GRCh38.91.exon
$ hisat2-build --ss Homo_sapiens.GRCh38.91.ss --exon Homo_sapiens.GRCh38.91.exon
Homo_sapiens.GRCh38.dna.primary_assembly.fa hisat2_hg38
```

HISAT2 alignment for each sample

```
$ hisat2 -p 6 --dta -x hisat2_hg38 -1 sample_pair1.fq.gz -2 sample_pair2.fq.gz -S
result.sam
$ samtools sort -@ 6 -o result.bam result.sam
```

#Transcript assembly with SR

```
$ samtools merge -@ 32 -nurlf –h result.bam -b good150bam_list stem_merge150good.bam
$ samtools sort -@ 32 -o stem_merge150good_sorted.bam stem_merge150good.bam
$ stringtie stem_merge150good_sorted.bam -G gencode.v32.annotation.gtf -o
short_read_150_samples.gtf -p 32 -c 1 -v &> short_read_150_samples.out
```

Isoseq pipeline for LR samples

```
$ ccs --numThreads 8 --noPolish --minPasses 1 H1_m54299_181207_142926.subreads.bam
H1_m54299_181207_142926.subreads.ccs_np.bam

$ lima H1_m54299_181207_142926.subreads.ccs_np.bam m54296_181207_094757.adapters.3p.fasta
H1_m54299_181207_142926.subreads.fl.bam --guess --isoseq --no-pbi --num-threads 32

$ isoseq3 refine H1_m54299_181207_142926.subreads.ccs_np.bam
m54296_181207_094757.adapters.5p.fasta H1_m54299_181207_142926.subreads.flnc.bam

$ bamtools convert -format fasta –in H1_m54299_181207_142926_reqA.subreads.flnc.bam -out
H1_m54299_181207_142926_reqA.subreads.flnc.fasta
```

#Alignment for LR samples, using GMAP

```
$ gmap_build -k 8 -d ens_hg38_vs91_gmap Homo_sapiens.GRCh38.dna.primary_assembly.fa
```

```
$ gmap -D ens_hg38_vs91_gmap -t 6 -d ens_hg38_vs91_gmap -f samse --max-intronlength-middle 2000000 --localsplicedist 4000000 --totallength 4000000 H1_m54299_181207_142926_reqA.subreads.flnc.fasta > H1_m54299_181207_142926_reqA.subreads.flnc.bam
$samtools merge -@ 32 -nurlf -h merged26.sam -b LR_bam_list LR_merged.bam
$samtools sort -@ 32 -o LR_merged_sorted.bam LR_merged.bam
```

#Transcript assembly for LR samples

```
$ stringtie LR_merged_sorted.bam -G gencode.v32.annotation.gtf –o long_read_4_samples.gtf -p 32 -c 1 -v &> long_read_4_samples.out
```

#Comparing SR and LR gtf files, done after unstranded transcripts were discarded

```
$ python2 compare_gtf.py –i short_read_150_samples_stranded.gtf -r long_read_4_samples_stranded.gtf -o SR_LR_matched.all -m SR_LR_matched.paired
```

#Merge SR and LR gtf files, using the result of compare_gtf.py

```
$ python2 update_sr_lr.py –s short_read_150_samples_stranded.gtf –l long_read_4_samples_stranded.gtf –m SR_LR_matched.paired –o SR_LR_merged.gtf
```

#Transcript quantifications for merged bam and individual SR samples

```
$ stringtie stem_merge150good_sorted.bam -G SR_LR_updated_200bp.gtf -o SR_LR_updated_200bp_count.gtf -e -B
$ stringtie SRR597894.bam -G SR_LR_200bp.gtf -o SR_LR_SRR597894.gtf -e -B
```

#Extracting expression matrix. The input is a directory of all samples from Stringtie quantification

```
$ python2 expression_count_all.py -i count_dir -c TPM -o SR_LR_updated_200bp_TPM_count.tsv
```

Code for scoring transcript coding potential

FEELnc coding potential

```
$ FEELnc_codpot.pl -i assembly_hPSC_final.gtf -a gencode.v32.pc_transcripts.fa -l
gencode.v32.lncRNA_transcripts.fa -g Homo_sapiens.GRCh38.dna.primary_assembly.fa  --
outname=assembly_hPSC_final -outdir=FEELnc_use
```

Code for the detection of TE sequence fragments inside transcripts

nhmmer command used to search against the assembled transcripts:

```
$ nhmmer -E 1e-10 --cpu 32 --dna --noali --tblout tblout.$out.tsv Dfam_edited.hmm $inp
>/dev/null
```

Table of TE-containing transcripts is in **Supplementary Table S3**, and at https://github.com/oaxiom/hesc_lincrna/blob/master/te_discovery/te_transcripts/transcript_table_merged.mapped.tsv.gz.

The full analysis pipeline for this section can be found online at: https://github.com/oaxiom/hesc_lincrna/tree/master/te_discovery/hmmer_dfam/data

Code for the preprocessing of mass spectrometry data and example spectrum search

The HipSci mzml data was converted to centroid (peakPicker) files using the MSGF+ (1) command:

```
$ msconvert $in --filter "peakPicking cwt msLevel=1-" --mzML --32 --outfile $out.cwt.mzML
```

Using the final set of putative protein sequences (masked_peptides.fa), and the HipSci data we then performed a search of the LC-MS/MS spectra using MSGF+ (1) with the command:

```
$ MSGFPlus.jar –s $in.cwt.mzML –thread 30 –d masked_peptides.fa –e 3 –inst 1 –t 20ppm –ti
1,2 –mod mods.txt –ntt 2 –tda 1 –o out.mzid
```

To reduce the search space and reduce false positive hits, the modifications (mods.txt) considered included a maximum of 3 modifications, and carbamidomethylation on Cys as a fixed modification, and the variable modifications: oxidation of Met, conversion of N-terminal Gln to pyro-Glu, deamidation of Asp and Glu, and acetylation at the N-terminus. The mods.txt file used was:

```
NumMods=3
# Static (fixed) modifications:
C2H3N1O1,C,fix,any,Carbamidomethyl      # Fixed Carbamidomethyl C
# Variable Modifications
O1,M,opt,any,Oxidation                  # Oxidation M
H-3N-1,Q,opt,N-term,Gln->pyro-Glu       # Pyro-glu from Q
H-1N-1O1,NQ,opt,any,Deamidated
C2H2O,*,opt,Prot-N-term,Acetyl          # Acetylation Protein N-term
```

A peptide was considered a hit if the target decoy q-value reported by MSGF+ was <0.05. Peptide hits for transcripts are in **Supplementary Table S5**. The full analysis pipeline for this section of the Supplementary Methods can be found online at: https://github.com/oaxiom/hesc_lincrna/tree/master/massspec

Code for single cell RNA-seq and analysis

The sc-RNA-seq reads were aligned to the hg38 genome using STARsolo (2), with the settings and using the appropriate whitelist barcode file (version 1 for Ref. (3), and version 2 for Ref. (4) and the sc-RNA-seq data generated in this project):

```
$ STAR --runRNGseed 42 --runThreadN 12 --readFilesCommand zcat  --outFilterMultimapNmax 100
--winAnchorMultimapNmax 100 --outSAMmultNmax 1 --outSAMtype BAM SortedByCoordinate --
twopassMode Basic --soloType Droplet --soloFeatures Gene --soloBarcodeReadLength 0 --
soloCBwhitelist versionX.txt --outSAMattributes NH HI AS nM CR CY UR UY --genomeDir
gtf_index/SAindex --outFileNamePrefix ss.$out --readFilesIn $p1 $p2
```

The BAM was then processed using te_counter (https://github.com/oaxiom/te_counter), which is a lightweight (although slower) implementation of the scTE algorithm that can accept custom indices (5):

Make an index, only needs to be done once:

```
$ te_genome -m custom -g 3ends_only.idx --gtf ends.gtf
```

Count the reads from the BAM file, demultiplexing the barcodes and UMIs. $white is the whitelist of barcodes from the 10x platform and must be correct for the version of 10x used.

```
$ te_count -m custom -g 3ends_only.idx -i $inp -o $out.tsv --sc --se --strand -w $white
```

The full analysis pipeline for this section of the Supplementary Methods can be found online at: https://github.com/oaxiom/hesc_lincrna/tree/master/singlecell

Supplementary References

1. Kim, S. and Pevzner, P.A. (2014) MS-GF+ makes progress towards a universal database search tool for proteomics. *Nat Commun*, **5**, 5277.

2. Dobin, A., Davis, C.A., Schlesinger, F., Drenkow, J., Zaleski, C., Jha, S., Batut, P., Chaisson, M. and Gingeras, T.R. (2013) STAR: ultrafast universal RNA-seq aligner. *Bioinformatics*, **29**, 15-21.

3. Nguyen, Q.H., Lukowski, S.W., Chiu, H.S., Senabouth, A., Bruxner, T.J.C., Christ, A.N., Palpant, N.J. and Powell, J.E. (2018) Single-cell RNA-seq of human induced pluripotent stem cells reveals cellular heterogeneity and cell state transitions between subpopulations. *Genome Res*, **28**, 1053-1066.

4. Chen, D., Sun, N., Hou, L., Kim, R., Faith, J., Aslanyan, M., Tao, Y., Zheng, Y., Fu, J., Liu, W., Kellis, M. and Clark, A. (2019) Human Primordial Germ Cells Are Specified from Lineage-Primed Progenitors. *Cell Rep*, **29**, 4568-4582 e4565.

5. He, J., Babarinde, I.A., Sun, L., Xu, S., Chen, R., Shi, J., Wei, Y., Li, Y., Ma, G., Zhuang, Q., Hutchins, A.P. and Chen, J. (2021) Identifying transposable element expression dynamics and heterogeneity during development at the single-cell level with a processing pipeline scTE. *Nat Commun*, **12**, 1456.

6. Fantom Consortium and the Riken PMI and CLST, Forrest, A.R., Kawaji, H., Rehli, M., Baillie, J.K., de Hoon, M.J., Haberle, V., Lassmann, T., Kulakovskiy, I.V., Lizio, M., Itoh, M., Andersson, R., Mungall, C.J., Meehan, T.F., Schmeier, S., Bertin, N., Jorgensen, M., Dimont, E., Arner, E., Schmidl, C., Schaefer, U., Medvedeva, Y.A., Plessy, C., Vitezic, M., Severin, J., Semple, C., Ishizu, Y., Young, R.S., Francescatto, M., Alam, I., Albanese, D., Altschuler, G.M., Arakawa, T.,

Archer, J.A., Arner, P., Babina, M., Rennie, S., Balwierz, P.J., Beckhouse, A.G., Pradhan-Bhatt, S., Blake, J.A., Blumenthal, A., Bodega, B., Bonetti, A., Briggs, J., Brombacher, F., Burroughs, A.M., Califano, A., Cannistraci, C.V., Carbajo, D., Chen, Y., Chierici, M., Ciani, Y., Clevers, H.C., Dalla, E., Davis, C.A., Detmar, M., Diehl, A.D., Dohi, T., Drablos, F., Edge, A.S., Edinger, M., Ekwall, K., Endoh, M., Enomoto, H., Fagiolini, M., Fairbairn, L., Fang, H., Farach-Carson, M.C., Faulkner, G.J., Favorov, A.V., Fisher, M.E., Frith, M.C., Fujita, R., Fukuda, S., Furlanello, C., Furino, M., Furusawa, J., Geijtenbeek, T.B., Gibson, A.P., Gingeras, T., Goldowitz, D., Gough, J., Guhl, S., Guler, R., Gustincich, S., Ha, T.J., Hamaguchi, M., Hara, M., Harbers, M., Harshbarger, J., Hasegawa, A., Hasegawa, Y., Hashimoto, T., Herlyn, M., Hitchens, K.J., Ho Sui, S.J., Hofmann, O.M., Hoof, I., Hori, F., Huminiecki, L., Iida, K., Ikawa, T., Jankovic, B.R., Jia, H., Joshi, A., Jurman, G., Kaczkowski, B., Kai, C., Kaida, K., Kaiho, A., Kajiyama, K., Kanamori-Katayama, M., Kasianov, A.S., Kasukawa, T., Katayama, S., Kato, S., Kawaguchi, S., Kawamoto, H., Kawamura, Y.I., Kawashima, T., Kempfle, J.S., Kenna, T.J., Kere, J., Khachigian, L.M., Kitamura, T., Klinken, S.P., Knox, A.J., Kojima, M., Kojima, S., Kondo, N., Koseki, H., Koyasu, S., Krampitz, S., Kubosaki, A., Kwon, A.T., Laros, J.F., Lee, W., Lennartsson, A., Li, K., Lilje, B., Lipovich, L., Mackay-Sim, A., Manabe, R., Mar, J.C., Marchand, B., Mathelier, A., Mejhert, N., Meynert, A., Mizuno, Y., de Lima Morais, D.A., Morikawa, H., Morimoto, M., Moro, K., Motakis, E., Motohashi, H., Mummery, C.L., Murata, M., Nagao-Sato, S., Nakachi, Y., Nakahara, F., Nakamura, T., Nakamura, Y., Nakazato, K., van Nimwegen, E., Ninomiya, N., Nishiyori, H., Noma, S., Noma, S., Noazaki, T., Ogishima, S., Ohkura, N., Ohimiya, H., Ohno, H., Ohshima, M., Okada-Hatakeyama, M., Okazaki, Y., Orlando, V., Ovchinnikov, D.A., Pain, A., Passier, R., Patrikakis, M., Persson, H., Piazza, S., Prendergast, J.G., Rackham, O.J., Ramilowski, J.A., Rashid, M., Ravasi, T., Rizzu, P., Roncador, M., Roy, S., Rye, M.B., Saijyo, E., Sajantila, A., Saka, A., Sakaguchi, S., Sakai, M., Sato, H., Savvi, S., Saxena, A., Schneider, C., Schultes, E.A., Schulze-Tanzil, G.G., Schwegmann, A., Sengstag, T., Sheng, G., Shimoji, H., Shimoni, Y., Shin, J.W., Simon, C., Sugiyama, D., Sugiyama, T., Suzuki, M., Suzuki, N., Swoboda, R.K., t Hoen, P.A., Tagami, M., Takahashi, N., Takai, J., Tanaka, H., Tatsukawa, H., Tatum, Z., Thompson, M., Toyodo, H., Toyoda, T., Valen, E., van de Wetering, M., van den Berg, L.M., Verado, R., Vijayan, D., Vorontsov, I.E., Wasserman, W.W., Watanabe, S., Wells, C.A., Winteringham, L.N., Wolvetang, E., Wood, E.J., Yamaguchi, Y., Yamamoto, M., Yoneda, M., Yonekura, Y., Yoshida, S., Zabierowski, S.E., Zhang, P.G., Zhao, X., Zucchelli, S., Summers, K.M., Suzuki, H., Daub, C.O., Kawai, J., Heutink, P., Hide, W., Freeman, T.C., Lenhard, B., Bajic, V.B., Taylor, M.S., Makeev, V.J., Sandelin, A., Hume, D.A., Carninci, P. and Hayashizaki, Y. (2014) A promoter-level mammalian expression atlas. *Nature*, **507**, 462-470.

7. Abugessaisa, I., Noguchi, S., Hasegawa, A., Harshbarger, J., Kondo, A., Lizio, M., Severin, J., Carninci, P., Kawaji, H. and Kasukawa, T. (2017) FANTOM5 CAGE profiles of human and mouse reprocessed for GRCh38 and GRCm38 genome assemblies. *Sci Data*, **4**, 170107.

8. Cheng, L.C., Zheng, D., Baljinnyam, E., Sun, F., Ogami, K., Yeung, P.L., Hoque, M., Lu, C.W., Manley, J.L. and Tian, B. (2020) Widespread transcript shortening through alternative polyadenylation in secretory cell differentiation. *Nat Commun*, **11**, 3182.

9. Dewannieux, M., Harper, F., Richaud, A., Letzelter, C., Ribet, D., Pierron, G. and Heidmann, T. (2006) Identification of an infectious progenitor for the multiple-copy HERV-K human endogenous retroelements. *Genome Res*, **16**, 1548-1556.

10. Jang, H.S., Shah, N.M., Du, A.Y., Dailey, Z.Z., Pehrsson, E.C., Godoy, P.M., Zhang, D., Li, D., Xing, X., Kim, S., O'Donnell, D., Gordon, J.I. and Wang, T. (2019) Transposable elements drive widespread expression of oncogenes in human cancers. *Nat Genet*, **51**, 611-617.

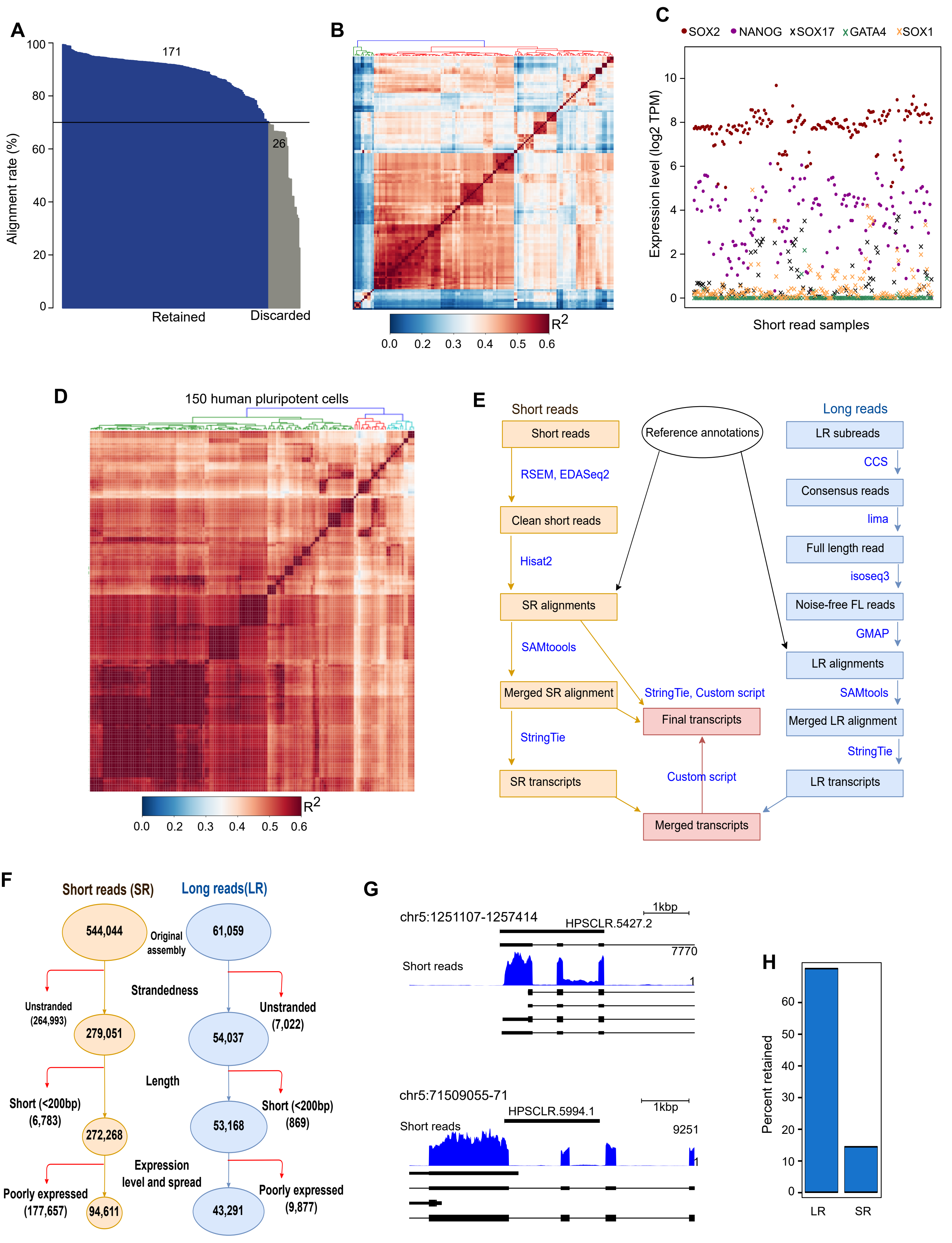
A
100
80
60
40
20
0
Alignment rate (%)
171
26
Retained
Discarded
B
R2
0.0 0.2 0.3 0.4 0.5 0.6
C
SOX2 NANOG SOX17 GATA4 SOX1
Expression level (log2 TPM)
Short read samples
D
150 human pluripotent cells
E
Short reads
Short reads
RSEM, EDASeq2
Clean short reads
Hisat2
SR alignments
SAMtoools
Merged SR alignment
StringTie
SR transcripts
Reference annotations
StringTie, Custom script
Final transcripts
Custom script
Merged transcripts
Long reads
LR subreads
CCS
Consensus reads
lima
Full length read
isoseq3
Noise-free FL reads
GMAP
LR alignments
SAMtools
Merged LR alignment
StringTie
LR transcripts
F
Short reads (SR)
Long reads(LR)
544,044
Original assembly
61,059
Strandedness
Unstranded (264,993)
279,051
Unstranded (7,022)
54,037
Length
Short (<200bp) (6,783)
272,268
53,168
Short (<200bp) (869)
Expression level and spread
Poorly expressed (177,657)
94,611
43,291
Poorly expressed (9,877)
G
chr5:1251107-1257414
1kbp
HPSCLR.5427.2
7770
Short reads
1
chr5:71509055-71
1kbp
HPSCLR.5994.1
Short reads
9251
1
H
Percent retained
LR
SR

Figure S1

Supplementary Figure S1 – Pipeline for the assembly of a hPSC-specific transcriptome

(A) Ranked alignment rate for all short read hPSC samples. As low read mapping is indicative of poor sample quality, an arbitrary threshold of 70% mapping was chosen for a qualified sample. The line indicates our cutoff of >70% of reads must map to the hg38 human genome assembly. 171 samples passed, 26 failed.

(B) Heatmap showing the sample-pairwise Pearson R^2 correlation score. Samples were clustered by their Euclidean distance. Only samples in the largest cluster that had an overall co-correlation >0.6 were retained.

(C) Plot showing RNA levels for all short read samples for two selected pluripotency genes (*SOX2*, *NANOG*) and three selected differentiation genes (*SOX17*, *SOX1* and *GATA4*). Samples were removed if they expressed high levels of differentiated marker genes (any gene log2(TPM) >2), or low levels of pluripotency genes (any pluripotency gene log2(TPM) <4). The x-axis short read samples are in random order.

(D) Heatmap showing the sample-pairwise Pearson R^2 correlation score for the final set of 150 selected hPSC samples.

(E) Schematic of the analysis pipeline used to assemble the short read transcripts and the long read transcripts, and to then merge the final transcripts. The key bioinformatics tools used at each step are indicated in blue.

(F) Flow chart showing the number of transcripts at each stage of the transcript filtering process. Step 1: Remove transcripts that could not be unambiguously identified to a specific strand. Step 2: Remove transcripts <200 nucleotides in length, as we did not take special steps for short transcripts. Step 3: Remove transcripts based on minimum RNA abundance levels.

(G) Genome view of two examples of long read transcripts (HPSCLR.5427.2 and HPSCLR.5994.1) that span introns or match an internal intron, and are not supported by short read data. Short read pileups are shown in blue. GENCODE transcripts are shown below the short read pileup tracks. Note how the long read transcript edges match perfectly with two exon boundaries, which is suggestive of incomplete RNA processing.

(H) Bar chart showing the percent of LR- and SR-based transcripts that passed the quality control filters.

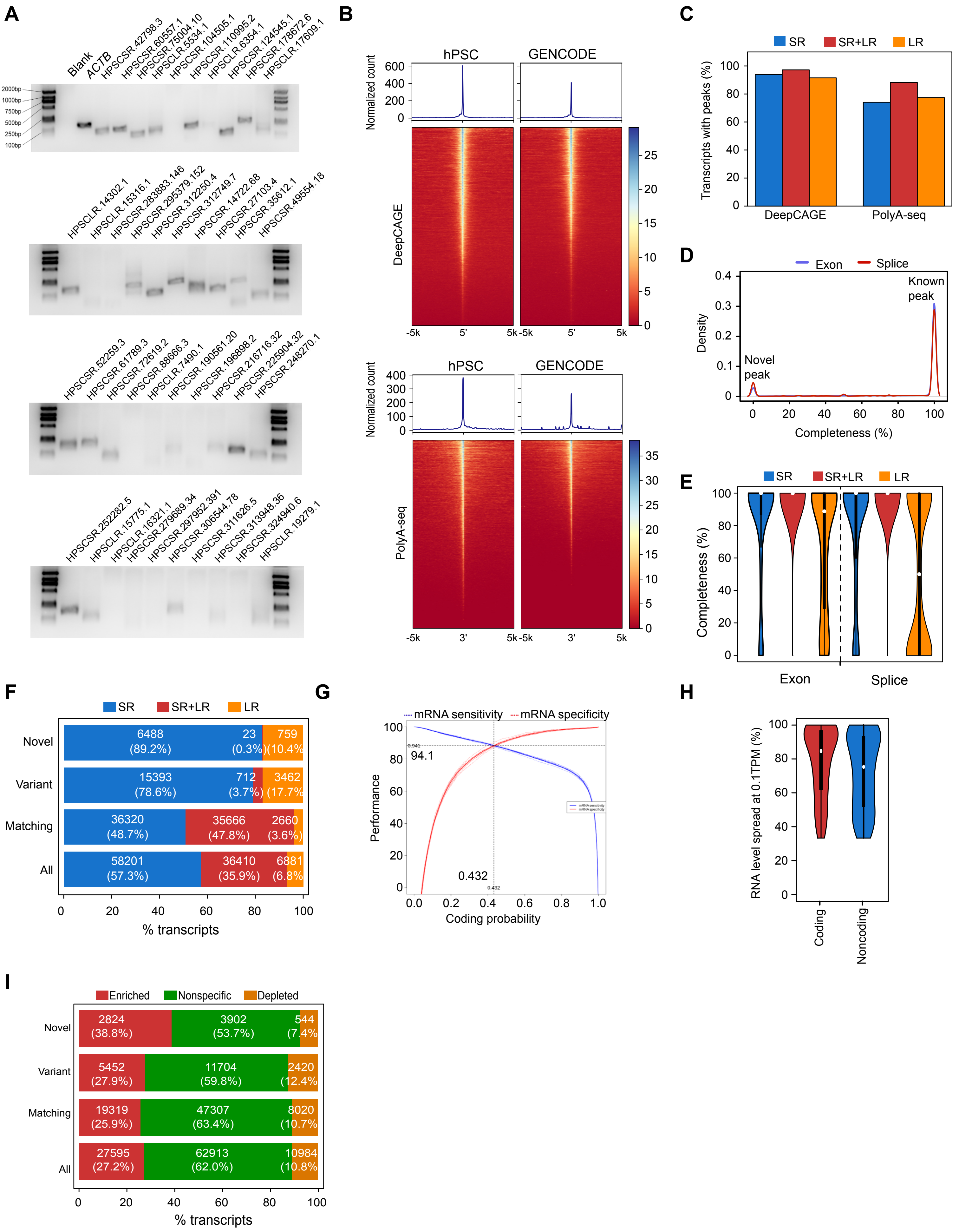
A
Blank
ACTB
HPSCSR.42798.3
HPSCSR.60557.1
HPSCSR.75004.10
HPSCLR.5534.1
HPSCSR.104505.1
HPSCSR.110995.2
HPSCLR.6354.1
HPSCSR.124545.1
HPSCSR.178672.6
HPSCLR.17609.1
2000bp
1000bp
750bp
500bp
250bp
100bp
HPSCLR.14302.1
HPSCLR.15316.1
HPSCSR.283883.146
HPSCSR.295379.152
HPSCSR.312250.4
HPSCSR.312749.7
HPSCLR.14722.68
HPSCLR.27103.4
HPSCSR.35612.1
HPSCSR.49554.18
HPSCSR.52259.3
HPSCSR.61789.3
HPSCSR.72619.2
HPSCSR.88666.3
HPSCLR.7490.1
HPSCSR.190561.20
HPSCSR.196898.2
HPSCSR.216716.32
HPSCSR.225904.32
HPSCSR.248270.1
HPSCSR.252282.5
HPSCLR.15775.1
HPSCLR.16321.1
HPSCSR.279689.34
HPSCSR.297952.391
HPSCSR.306544.78
HPSCSR.311626.5
HPSCSR.313948.36
HPSCSR.324940.6
HPSCLR.19279.1
B
hPSC
GENCODE
Normalized count
DeepCAGE
-5k
5'
5k
PolyA-seq
3'
C
SR
SR+LR
LR
Transcripts with peaks (%)
DeepCAGE
PolyA-seq
D
Exon
Splice
Density
Known peak
Novel peak
Completeness (%)
E
Completeness (%)
Exon
Splice
F
Novel
Variant
Matching
All
6488 (89.2%)
23 (0.3%)
759 (10.4%)
15393 (78.6%)
712 (3.7%)
3462 (17.7%)
36320 (48.7%)
35666 (47.8%)
2660 (3.6%)
58201 (57.3%)
36410 (35.9%)
6881 (6.8%)
% transcripts
G
mRNA sensitivity
mRNA specificity
Performance
94.1
0.432
Coding probability
H
RNA level spread at 0.1TPM (%)
Coding
Noncoding
I
Enriched
Nonspecific
Depleted
2824 (38.8%)
3902 (53.7%)
544 (7.4%)
5452 (27.9%)
11704 (59.8%)
2420 (12.4%)
19319 (25.9%)
47307 (63.4%)
8020 (10.7%)
27595 (27.2%)
62913 (62.0%)
10984 (10.8%)
% transcripts

Figure S2

Supplementary Figure S2 – Validation of the transcript assembly and non coding RNA determination.

(A) PCR gel electrophoresis result for 40 selected hPSC novel transcripts. RNA was extracted from H1 hESCs, reverse transcribed and subjected to PCR using primers specific to each indicated transcript. The primers spanned at least one intron splice site. PCR was run for 40 cycles to achieve saturation. Beta-actin (*ACTB*) is included as a control.

(B) Heatmaps and line charts showing the pileup of DeepCAGE and polyA-seq pileup data centered on the 5' (deepCAGE) or 3' (polyA-seq) of the transcript assembly reported here (hPSC) or on the GENCODE transcriptome. For heatmaps, each row is a transcript in the indicated transcript assembly (this assembly, hPSC, or from GENCODE). Rows are sorted by their mean intensity. DeepCAGE data was from (6,7), the DeepCAGE track was generated by merging hESC (H1 and H9 line) and iPSC (unidentified line) deepCAGE data. The polyA-seq data was from H1 hESCs, from the accession GSE138759 (8).

(C) Percentage of transcripts with a deepCAGE peak at the 5' end of the transcript, and the percentage with polyA-seq support, for SR, LR or SR+LR transcripts.

(D) Completeness for each transcript, defined as the percent of exons or splice junctions that matched compared to the closest GENCODE exon. A 100% match represents a perfect base pair match for the genomic locations of all the exons or splice sites in a transcript, except the 5' and 3' ends of the transcript, which were not considered as they both tend to be fuzzier. A 0% match represents a novel transcript that has no matching exon with GENCODE. The 'exon' match (in blue) is the score of all matching nucleotides in an exon that matches to GENCODE, whilst the 'splice' curve (in red) considers only the positions of the exons.

(E) Exon and splice completeness, as defined in panel D, for transcripts that are supported by SR, LR or SR+LR.

(F) Percentage of transcripts that have SR, LR or SR+LR support, divided by their relationship to a GENCODE transcript: matching (have all internal exons matching to a GENCODE transcript), are variant (have any 1 nucleotide overlap in an exon to any GENCODE transcript), or are novel (have no match to any GENCODE transcript).

(G) FEELnc coding probability plot for determination of coding potential threshold. FEELnc determines the threshold to call coding and noncoding RNAs based on where the two sensitivity lines cross. In this case, the threshold was determined to be 0.432.

(H) Violin plot showing the spread of RNA levels (in transcripts per million), for the percentage of coding and noncoding transcripts with at least a TPM of 0.1.

(I) Percentage of transcripts that are enriched, depleted or nonspecifically expressed in hPSCs, broken down by their transcript class compared to GENCODE.

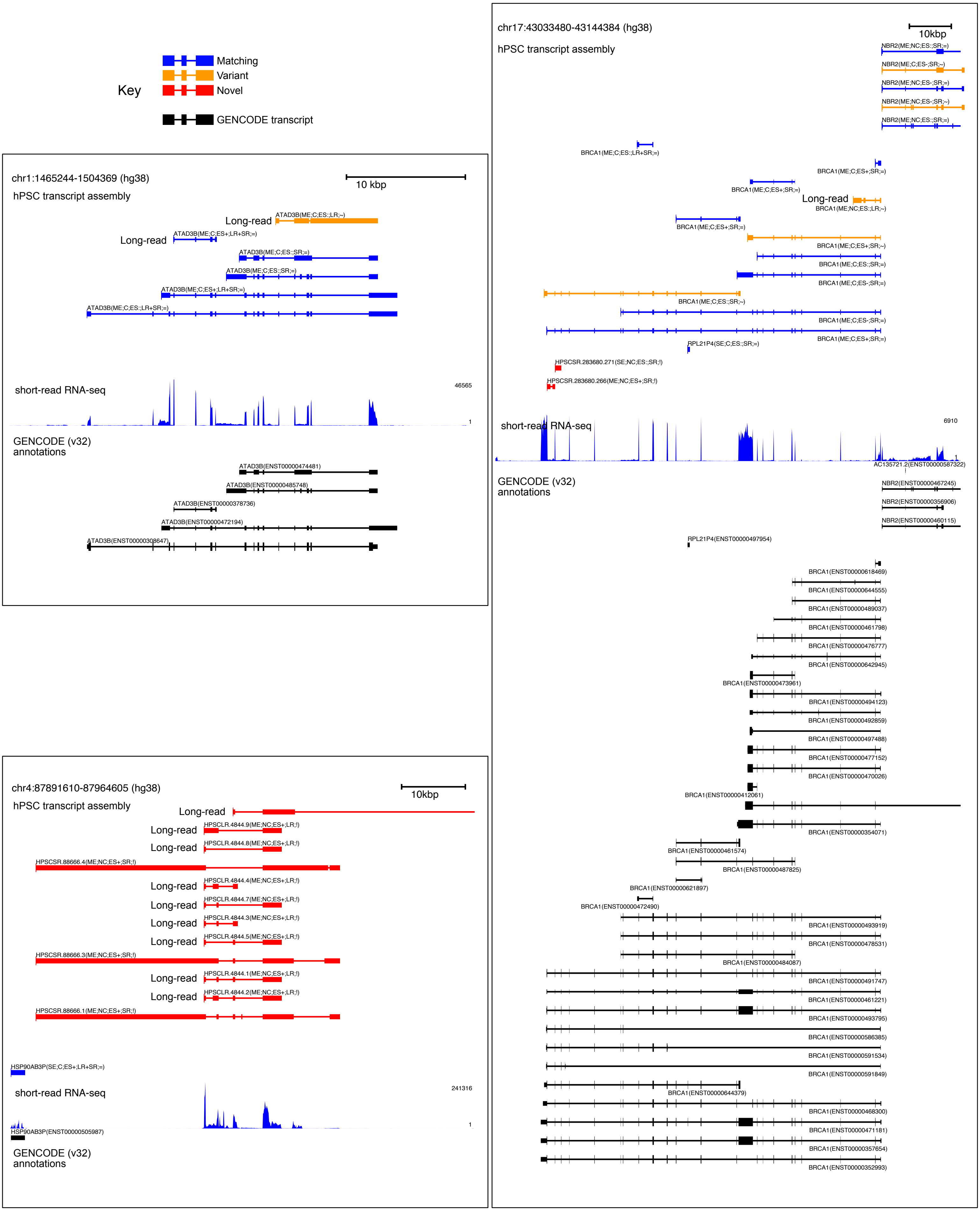

Key
Matching
Variant
Novel
GENCODE transcript
chr1:1465244-1504369 (hg38)
hPSC transcript assembly
10 kbp
Long-read
ATAD3B(ME;C;ES:;LR;~)
Long-read
ATAD3B(ME;C;ES+;LR+SR;=)
ATAD3B(ME;C;ES:;SR;=)
ATAD3B(ME;C;ES:;SR;=)
ATAD3B(ME;C;ES+;LR+SR;=)
ATAD3B(ME;C;ES:;LR+SR;=)
short-read RNA-seq
46565
1
GENCODE (v32) annotations
ATAD3B(ENST00000474481)
ATAD3B(ENST00000485748)
ATAD3B(ENST00000378736)
ATAD3B(ENST00000472194)
ATAD3B(ENST00000308647)
chr4:87891610-87964605 (hg38)
hPSC transcript assembly
10kbp
Long-read
HPSCLR.4844.9(ME;NC;ES+;LR;!)
HPSCLR.4844.8(ME;NC;ES+;LR;!)
HPSCSR.88666.4(ME;NC;ES+;SR;!)
HPSCLR.4844.4(ME;NC;ES+;LR;!)
HPSCLR.4844.7(ME;NC;ES+;LR;!)
HPSCLR.4844.3(ME;NC;ES+;LR;!)
HPSCLR.4844.5(ME;NC;ES+;LR;!)
HPSCSR.88666.3(ME;NC;ES+;SR;!)
HPSCLR.4844.1(ME;NC;ES+;LR;!)
HPSCLR.4844.2(ME;NC;ES+;LR;!)
HPSCSR.88666.1(ME;NC;ES+;SR;!)
HSP90AB3P(SE;C;ES+;LR+SR;=)
short-read RNA-seq
241316
1
HSP90AB3P(ENST00000505987)
GENCODE (v32) annotations
chr17:43033480-43144384 (hg38)
hPSC transcript assembly
10kbp
NBR2(ME;NC;ES:;SR;=)
NBR2(ME;C;ES-:;SR;~)
NBR2(ME;NC;ES-;SR;=)
NBR2(ME;NC;ES:;SR;~)
NBR2(ME;NC;ES:;SR;=)
BRCA1(ME;C;ES:;LR+SR;=)
BRCA1(ME;C;ES+;SR;=)
BRCA1(ME;C;ES+;SR;=)
Long-read
BRCA1(ME;NC;ES:;LR;~)
BRCA1(ME;C;ES+;SR;=)
BRCA1(ME;C;ES+;SR;~)
BRCA1(ME;C;ES:;SR;=)
BRCA1(ME;C;ES-;SR;=)
BRCA1(ME;C;ES:;SR;~)
BRCA1(ME;C;ES-;SR;=)
BRCA1(ME;C;ES+;SR;=)
RPL21P4(SE;C;ES:;SR;=)
HPSCSR.283680.271(SE;NC;ES:;SR;!)
HPSCSR.283680.266(ME;NC;ES+;SR;!)
short-read RNA-seq
6910
1
AC135721.2(ENST00000587322)
GENCODE (v32) annotations
NBR2(ENST00000467245)
NBR2(ENST00000356906)
NBR2(ENST00000460115)
RPL21P4(ENST00000497954)
BRCA1(ENST00000618469)
BRCA1(ENST00000644555)
BRCA1(ENST00000489037)
BRCA1(ENST00000461798)
BRCA1(ENST00000476777)
BRCA1(ENST00000642945)
BRCA1(ENST00000473961)
BRCA1(ENST00000494123)
BRCA1(ENST00000492859)
BRCA1(ENST00000497488)
BRCA1(ENST00000477152)
BRCA1(ENST00000470026)
BRCA1(ENST00000412061)
BRCA1(ENST00000354071)
BRCA1(ENST00000461574)
BRCA1(ENST00000487825)
BRCA1(ENST00000621897)
BRCA1(ENST00000472490)
BRCA1(ENST00000493919)
BRCA1(ENST00000478531)
BRCA1(ENST00000484087)
BRCA1(ENST00000491747)
BRCA1(ENST00000461221)
BRCA1(ENST00000493795)
BRCA1(ENST00000586385)
BRCA1(ENST00000591534)
BRCA1(ENST00000591849)
BRCA1(ENST00000644379)
BRCA1(ENST00000468300)
BRCA1(ENST00000471181)
BRCA1(ENST00000357654)
BRCA1(ENST00000352993)

Figure S3

Supplementary Figure S3 – Example genome views of selected matching, variant and novel transcripts.

All genome views show three parts.

(1) **hPSC transcript assembly**, described here. Matching transcripts (that have identical internal exon and splice sites compared to a GENCODE transcript) are marked in blue. Variant transcripts that have any 1 bp overlap versus a GENCODE transcript are marked in orange, and novel transcripts with no overlap to any GENCODE transcript are marked in red. Transcripts that are assembled from long read data are indicated.

(2) **short read RNA-seq**. pileup data from the short read RNA-seq,

(3) **GENCODE (v32) annotations**. Matching transcripts in the hPSC-transcript assembly are marked in blue, variants are marked in orange, and novel transcripts are marked in red. Transcripts assembled from the long read data are indicated as 'Long read'. Transcripts assembled from short read data are not labelled.

The figure shows three genomic loci, one showing the *ATAD3B* locus, which has all five GENCODE annotated transcripts and a variant transcript. The second locus shows the *BRCA1* and *NBR2* genomic locus. This locus is more complex, and contains 8 out of 34 GENCODE transcripts for *BRCA1*, and 2 variant transcripts not in GENCODE. The third locus shows 12 novel transcripts close to the *HSP90AB3P* gene.

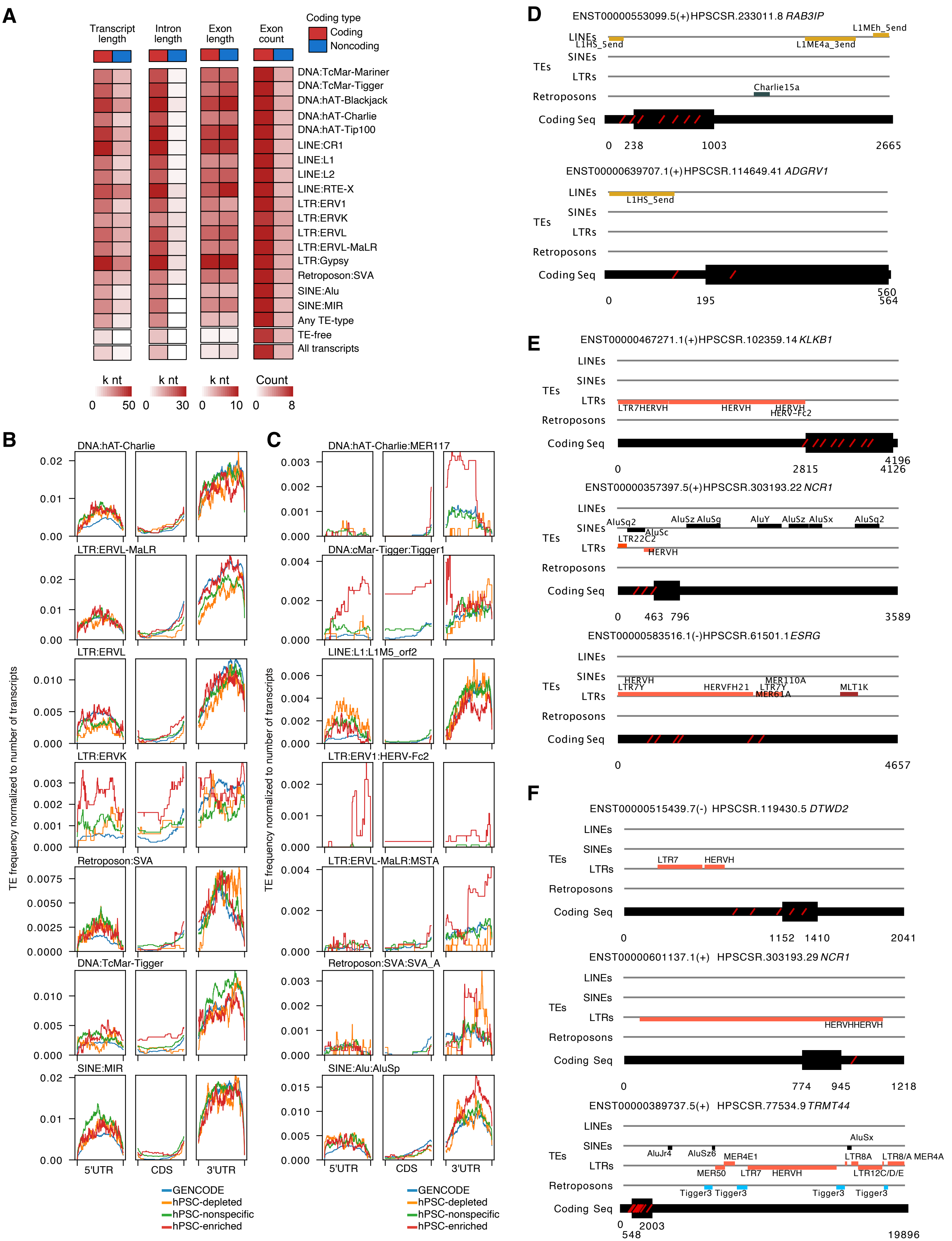
A
Transcript length
Intron length
Exon length
Exon count
Coding type
Coding
Noncoding
DNA:TcMar-Mariner
DNA:TcMar-Tigger
DNA:hAT-Blackjack
DNA:hAT-Charlie
DNA:hAT-Tip100
LINE:CR1
LINE:L1
LINE:L2
LINE:RTE-X
LTR:ERV1
LTR:ERVK
LTR:ERVL
LTR:ERVL-MaLR
LTR:Gypsy
Retroposon:SVA
SINE:Alu
SINE:MIR
Any TE-type
TE-free
All transcripts
k nt
0 50
k nt
0 30
k nt
0 10
Count
0 8
B
DNA:hAT-Charlie
LTR:ERVL-MaLR
LTR:ERVL
LTR:ERVK
Retroposon:SVA
DNA:TcMar-Tigger
SINE:MIR
TE frequency normalized to number of transcripts
5'UTR
CDS
3'UTR
GENCODE
hPSC-depleted
hPSC-nonspecific
hPSC-enriched
C
DNA:hAT-Charlie:MER117
DNA:cMar-Tigger:Tigger1
LINE:L1:L1M5_orf2
LTR:ERV1:HERV-Fc2
LTR:ERVL-MaLR:MSTA
Retroposon:SVA:SVA_A
SINE:Alu:AluSp
TE frequency normalized to number of transcripts
5'UTR
CDS
3'UTR
GENCODE
hPSC-depleted
hPSC-nonspecific
hPSC-enriched
D
ENST00000553099.5(+)HPSCSR.233011.8 RAB3IP
ENST00000639707.1(+)HPSCSR.114649.41 ADGRV1
E
ENST00000467271.1(+)HPSCSR.102359.14 KLKB1
ENST00000357397.5(+)HPSCSR.303193.22 NCR1
ENST00000583516.1(-)HPSCSR.61501.1 ESRG
F
ENST00000515439.7(-) HPSCSR.119430.5 DTWD2
ENST00000601137.1(+) HPSCSR.303193.29 NCR1
ENST00000389737.5(+) HPSCSR.77534.9 TRMT44
TEs
LINEs
SINEs
LTRs
Retroposons
Coding Seq

Figure S4

Supplementary Figure S4 – Examples of TE sequences inside transcripts.

(A) Heatmap showing several properties of coding and noncoding transcripts with the indicated TE inside their sequence, compared to all transcripts of TE-free transcripts. Transcript properties include: Total transcript (excluding introns) length, the average length of an intron, the average length of an exon, and the average number of exons. Note that a transcript containing multiple types of TE would be allocated to multiple categories.

(B) Line plots showing the TE frequency normalized to the number of transcripts, divided by whether they are hPSC-enriched, -nonspecific or -depleted, or for all GENCODE transcripts. Only protein-coding transcripts are shown, and they are divided into their 5' UTR, CDS or 3'UTR, and scaled to the same length. The blue line indicates all GENCODE transcripts, the red, green and yellow lines indicate hPSC categories. The left plots show all transcripts, and the right shows variant transcripts only. This plot shows the

(C) As in panel B, but for the indicated TE subtypes.

(D) Domain plots for selected transcripts containing an L1HS_5end. The location of TEs in the LINE, SINE LTR or DNA/SVAs (Retroposons) are indicated. The CDS is indicted by a think black box (unless it is non coding), and the numbers below the 'coding seq' indicate the start of the indicated regions in mRNA coordinates. Locations of splice sites are indicated by a slanted red line.

(E) As in panel D, but showing LTR7 and HERVH TE-containing transcripts.

(F) As in panel D, but showing LTR7 and HERVH in the middle of transcripts.

A

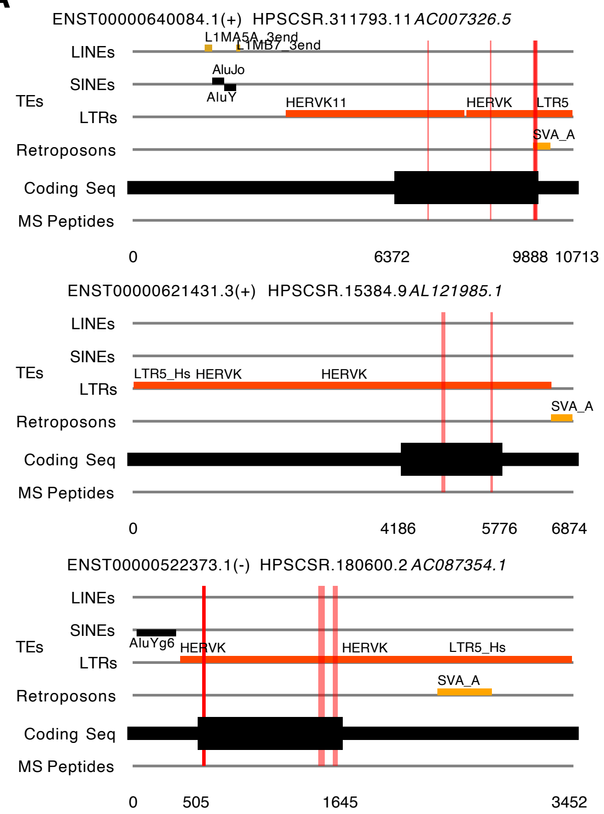

C

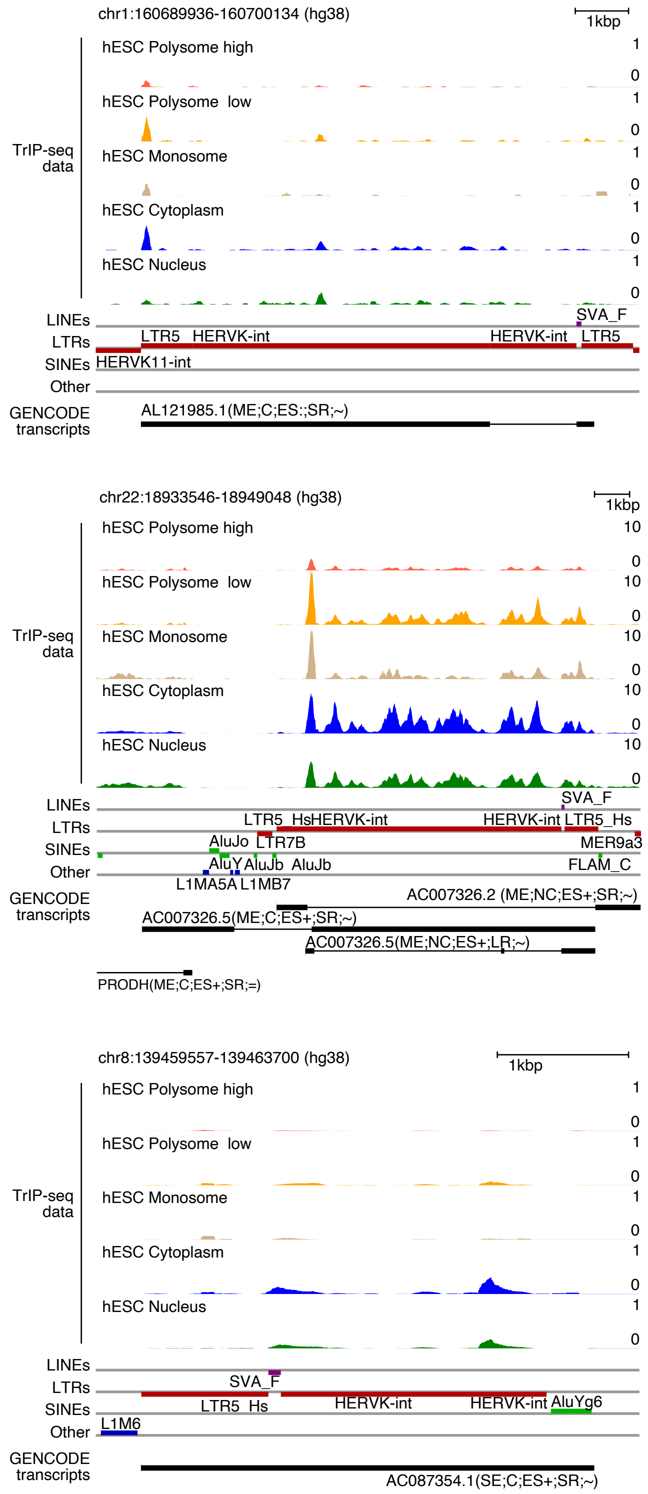

B

gag (17 unique peptides)

YASYLSFIK
MGQTKSKIKSKYASYLSFIKILLKRGGVKVSTKNLIKLFQIIEQFCPWFPEQGTLDLKDW

SCVTDCEEEAG
ATLEPFQTGEDIVSVSDAPK
IIPLTVWNDWAIIK
KRIGKELKQAGRKGNIIPLTVWNDWAIIKAALEPFQTEEDSVSVSDAPGSCIIDCNENTR

TESQQGTESSHCK LGEGGPESLGPSEPK
KKSQKETEGLHCEYVAEPVMAQSTQNVDYNQLQEVIYPETLKLEGKGPELVGPSESKPRG

TQPLVVYQYR
TSPLPAGQVPVTLQPQKQVKENKTQPPVAYQYWPPAELQYRPPPESQYGYPGMPPAPQGR

APYPQPPTRRLNPTAPPSRQGSELHEIIDKSRKEGDTEAWQFPVTLEPMPPGEGAQEGEP

LTPYDWEILAK
LIPYDWEILAK
TLLDSIAHGNR SSLSP
DMKEGVKQYGPNSPYMR SSLSS
PTVEARYKSFSIKMLKDMKEGVKQYGPNSPYMRTLLDSIAHGHRLIPYDWEILAKSSLSP

SQYLQFK
SQFLQFK
TWWIDGVQEQVR
SQFLQFKTWWIDGVQEQVRRNRAANPPVNIDADQLLGIGQNWSTISQQALMQNEAIEQVR

IQDPGTAFPINSI-R EPYPDFVAR
AICLRAWEKIQDPGSTCPSFNTVRQGSKEPYPDFVARLQDVAQKSIADEKARKVIVELMA

VPAGVDVITEYVK
YENANPECQSAIKPLKGKVPAGSDVISEYVKACDGIGGAMHKAMLMAQAITGVVLGGQVR

TFGGKCYNCGQIGHLKKNCPVLNKQNITIQATTTGREPPDLCPRCKKGKHWASQCRSKFD

RGQPQAPQQTGAFPVQLFVPQGFQGQQPLQK
KNGQPLSGNEQRGQPQAPQQTGAFPIQPFVPQGFQGQQPPLSQVFQGISQLPQYNNCPPP

QAAVQQ

pol (2 unique peptides)

NKSKKRRNRVSFLGAATVEPPKPIPLTWKTEKPVWVNQWPLPKQKLEALHLLANEQLEKG

HIEPSFSPWNSPVFVIQKKSGKWRMLTDLRAVNAVIQPMGPLQPGLPSPAMIPKDWPLII

IDLKDCFFTIPLAEQDCEKFAFTIPAINNKEPATRFQWKVLPQGMLNSPTICQTFVGRAL

QPVREKFSDCYIIHYIDDILCAAETKDKLIDCYTFLQAEVANAGLAIASDKIQTSTPFHY

GDS
LGMQIENRKIKPQKIEIRKDTLKTLNDFQKLLGDINWIRPTLGIPTYAMSNLFSILRGDS

DLNSKRMLTPEATKEIK
DLNSKRMLTPEATKEIKLVEEKIQSAQINRIDPLAPLQLLIFATAHSPTGIIIQNTDLVE

WSFLPHSTVKTFTLYLDQIATLIGQTRLRIIKLCGNDPDKIVVPLTKEQVRQAFINSGAW

QIGLANFVGIIDNHYPKTKIFQFLKLTTWILPKITRREPLENALTVFTDGSSNGKAAYTG

PKERVIKTPYQSAQRAELVAVITVLQDFDQPINIISDSAYVVQATRDVETALIKYSMDDQ

RNFPFYITHIR
LNQLFNLLQQTVRKRNFPFYITHIRAHTNLPGPLTKANEQADLLVSSALIKAQELHALTH

VNAAGLKNKFDVTWKQAKDIVQHCTQCQVLHLPTQEAGVNPRGLCPNALWQMDVTHVPSF

GRLSYVHVTVDTYSHFIWATCQTGESTSHVKKHLLSCFAVMGVPEKIKTDNGPGYCSKAF

QKFLSQWKISHTTGIPYNSQGQAIVERTNRTLKTQLVKQKEGGDSKECTTPQMQLNLALY

TLNFLNIYRNQTTTSAEQHLTGKKNSPHEGKLIWWKDNKNKTWEIGKVITWGRGFACVSP

GENQLPVWIPTRHLKFYNEPIRDAKKSTSAETETPQSSTVDSQDEQNGDVRRTDEVAIHQ

EGRAADLGTTKEADAVSYKISREHKGDTNPREYAACSLDDCINGGKSPYACRSSCS

env (4 unique peptides)

MNPSEMQRKAPPR MNKMVTSEEQMKLPSTK
MNPSEMQRKAPPRRRRHRNRAPLTHKMNKMVTSEEQMKLPSTKKAEPPTWAQLKKLTQLA

TKYLENTKVTQTPESMLLAALMIVSMVVSLPMPAGAAAANYTYWAYVPFPPLIRAVTWMD

NPIEVYVNDSVWVPGPIDDRCPAKPEEEGMMINISIGYRYPPICLGRAPGCLMPAVQNWL

VEVPTVSPISRFTYHMVSGMSLRPRVNYLQDFSYQRSLKFRPKGKPCPKEIPKESKNTEV

LVWEECVANSAVILQNNEFGTIIDWAPRGQFYHNCSGQTQSCPSAQVSPAVDSDLTESLD

LQSFYPWEWGEK LMMAQSPIRIWK
KHKHKKLQSFYPWEWGEKGISTPRPKIISPVSGPEHPELWRLTVASHHIRIWSGNQTLET

RDRKPFYTVDLNSSLTVPLQSCVKPPYMLVVGNIVIKPDSQTITCENCRLLTCIDSTFNW

QHRILLVRAREGVWIPVSMDRPWEASPSIHILTEVLKGVLNRSKRFIFTLIAVIMGLIAV

TATAAVAGVALHSSVQSVNFVNDWQKNSTRLWNSQSSIDQKLANQINDLRQTVIWMGDRL

MSLEHRFQLQCDWNTSDFCITPQIYNESEHHWDMVRRHLQGREDNLTLDISKLKEQIFEA

SKAHLNLVPGTEAIAGVADGLANLNPVTWVKTIGSTTIINLILILVCLFCLLLVCRCTQQ

LRRDSDHRERAMMTMAVLSKRKGGNVGKSKRDQIVTVSV

Figure S5

Supplementary Figure S5 – Mass spec peptides derived from HERVK transcripts

(A) Domain plots for three selected transcripts containing a HERVK or LTR7. The location of TEs in the LINE, SINE LTR or DNA/SVAs are indicated. The CDS is indicted by a think black box, and the numbers below the 'coding seq' indicate the start of the indicated regions in mRNA coordinates. Locations of splice sites are indicated by a slanted red line. The vertical red boxes indicate the positions for MS-detected peptides.

(B) Alignment of the mass spec detected peptides (marked in red), found in this study, against a hypothetical progenitor version of the HERVK viral proteins *gag*, *pol* and *env* (9).

(C) Genome views of the transcripts shown in panel A, with pileup RNA-seq data for the indicated nuclear, cytoplasmic or ribosome-bound fractions. The location of TEs in the genome is indicated in the lines below the pileup plots, and transcripts are marked below the TEs.

A

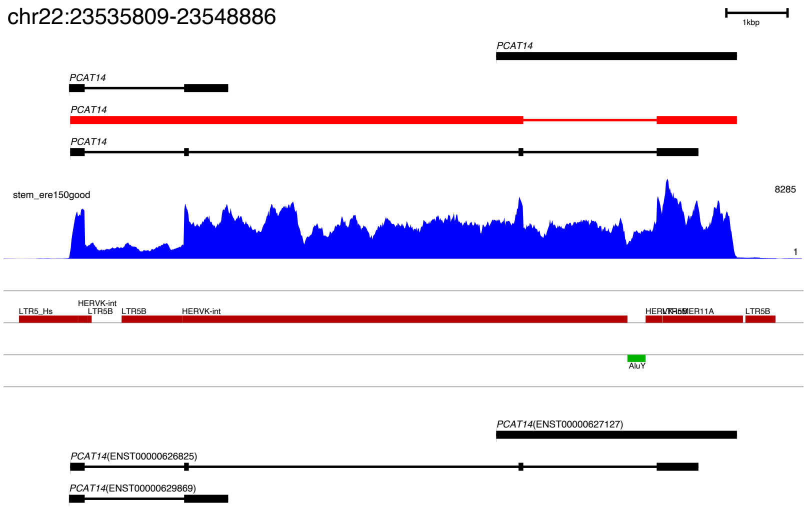

B

```
PCAT14      ----MGQTESKYASYLSFIKILLRRGGVRASTENLITLFQTIEQFCPWFPEQGTLDLKDW      56
GAG         MGQTKSKIKSKYASYLSFIKILLKRGGVKVSTKNLIKLFQIIEQFCPWFPEQGTLDLKDW      60
                .: :*************:****:.**:***.*** ******************

PCAT14      EKIGKELKQANREGKIIPLTVWNDWAIIKATLEPFQTGEDIVSVSDAPKSCVTDCEEEAG      116
GAG         KRIGKELKQAGRKGNIIPLTVWNDWAIIKAALEPFQTEEDSVSVSDAPGSCIIDCNENTR      120
            ::********.*:*:***************:****** ** ******* **: **:*::

PCAT14      TESQQGTESSHCKYVAESVMAQSTQNVDYSQLQEIIYPESSKLGEGGPESLGPSEPKPRS      176
GAG         KKSQKETEGLHCEYVAEPVMAQSTQNVDYNQLQEVIYPETLKLEGKGPELVGPSESKPRG      180
            .:**: **. **:**** ***********.****:****: **   *** :**** ***.

PCAT14      PSTPPPVVQMPVTLQPQTQVRQAQTPRENQVERDRVSIPAMPTQIQYPQYQPVENKTQPL      236
GAG         TS-PLPAGQVPVTLQPQKQV--------------------------------KENKTQPP      207
             * * *. *:*******.**                                ******

PCAT14      VVYQYRLPTELQYRPPSEVQYRPQAVCPVPNSTAPYQQPTAMASNSPATQDAALYPQPPT      296
GAG         VAYQYWPPAELQYRPPPESQYGYPGMPPAP-------------------QGRAPYPQPPT      248
            *.***  *:******* * **   .: *.*                   *. * ******

PCAT14      VRLNPTASRSGQGGALHAVIDEARKQGDLEAWRFLVILQLVQAGEETQVGAPARAETRCE      356
GAG         RRLNPTAPPSRQGSELHEIIDKSRKEGDTEAWQFPVTLEPMPPGEGAQEGEPPTVEARYK      308
             ******  * **. ** :**::**:** ***:* *:  :   ** :* * *  .*:* :

PCAT14      PFTMKMLKDIKEGVKQYGSNSPYIRTLLDSIAHGNRLTPYDWEILAKSSLSSSQYLQFKT      416
GAG         SFSIKMLKDMKEGVKQYGPNSPYMRTLLDSIAHGHRLIPYDWEILAKSSLSPSQFLQFKT      368
             *::*****:******** ****:**********:** ************* **:*****

PCAT14      WWIDGVQEQVRKNQATKPTVNIDADQLLGTGPNWSTINQQSVMQNEAIEQVRAICLRAWG      476
GAG         WWIDGVQEQVRRNRAANPPVNIDADQLLGIGQNWSTISQQALMQNEAIEQVRAICLRAWE      428
            ***********:*:*::* ********** * *****.**::*****************

PCAT14      KIQDPGTAFP-INSIRQGSKEPYPDFVARLQDAAQKSITDDNARKVIVELMAYENANPEC      535
GAG         KIQDPGSTCPSFNTVRQGSKEPYPDFVARLQDVAQKSIADEKARKVIVELMAYENANPEC      488
            ******:: * :*::*****************.*****:*::******************

PCAT14      QSAIKPLKGKVPAGVDVITEYVKACDGIGGAMHKAMLMAQAMRGLTLGGQVRTFGKKCYN      595
GAG         QSAIKPLKGKVPAGSDVISEYVKACDGIGGAMHKAMLMAQAITGVVLGGQVRTFGGKCYN      548
            ************** ***:*********************: *:.********* ****

PCAT14      CGQIGHLKRSCPGLNKQNIINQAINSKK--------------------------------      623
GAG         CGQIGHLKKNCPVLNKQNITIQATTTGREPPDLCPRCKKGKHWASQCRSKFDKNGQPLSG      608
            ********:.** ******  ** .: :

PCAT14      --------------------------------------------------------      623
GAG         NEQRGQPQAPQQTGAFPIQPFVPQGFQGQQPPLSQVFQGISQLPQYNNCPPPQAAVQQ      666
```

C

```
PCAT14_orf-57_POL      ------------------------------------------------------------ 0
PCAT14_orf-91_POL      ------------------------------------------------------------ 0
PCAT14_orf-52_POL      ------------------------------------------------------------ 0
POL                    NKSKKRRNRVSFLGAATVEPPKPIPLTWKTEKPVWVNQWPLPKQKLEALHLLANEQLEKG 60

PCAT14_orf-57_POL      ------------------------------------------------------------ 0
PCAT14_orf-91_POL      ------------------------------------------------------------ 0
PCAT14_orf-52_POL      ------------------------------------------------------------ 0
POL                    HIEPSFSPWNSPVFVIQKKSGKWRMLTDLRAVNAVIQPMGPLQPGLPSPAMIPKDWPLII 120

PCAT14_orf-57_POL      ------------------------------------------------------------ 0
PCAT14_orf-91_POL      ------------------------------------------------------------ 0
PCAT14_orf-52_POL      ------------------------------------------------------------ 0
POL                    IDLKDCFFTIPLAEQDCEKFAFTIPAINNKEPATRFQWKVLPQGMLNSPTICQTFVGRAL 180

PCAT14_orf-57_POL      ------------------------------------------------------------ 0
PCAT14_orf-91_POL      ------------------------------------------------------------ 0
PCAT14_orf-52_POL      ------------------------------------------------------------ 0
POL                    QPVREKFSDCYIIHYIDDILCAAETKDKLIDCYTFLQAEVANAGLAIASDKIQTSTPFHY 240

PCAT14_orf-57_POL      ------------------------------------------------------------ 0
PCAT14_orf-91_POL      ------------------------------------------------------------ 0
PCAT14_orf-52_POL      ------------------------------------------------MSNLFSFLRGDS 12
POL                    LGMQIENRKIKPQKIEIRKDTLKTLNDFQKLLGDINWIRPTLGIPTYAMSNLFSILRGDS 300

PCAT14_orf-57_POL      ------------------------------------------------------------ 0
PCAT14_orf-91_POL      ------------------------------------------------------------ 0
PCAT14_orf-52_POL      ELNSERTLTPEATKEIKLIEEKIRSAQVNRIDHLAPLQILIFATAHSLTGIIVQNTDLVE 72
POL                    DLNSKRMLTPEATKEIKLVEEKIQSAQINRIDPLAPLQLLIFATAHSPTGIIIQNTDLVE 360

PCAT14_orf-57_POL      ------------------------------------------------------------ 0
PCAT14_orf-91_POL      ------------------------------------------------------------ 0
PCAT14_orf-52_POL      WSFLPHSTIKTFTLYLDQMATLIGQGRL-------------------------------- 100
POL                    WSFLPHSTVKTFTLYLDQIATLIGQTRLRIIKLCGNDPDKIVVPLTKEQVRQAFINSGAW 420

PCAT14_orf-57_POL      ------------------------------------------------------------ 0
PCAT14_orf-91_POL      ------------------------------------------------------------ 0
PCAT14_orf-52_POL      ------------------------------------------------------------ 100
POL                    QIGLANFVGIIDNHYPKTKIFQFLKLTTWILPKITRREPLENALTVFTDGSSNGKAAYTG 480

PCAT14_orf-57_POL      ------------------------------------------------------------ 0
PCAT14_orf-91_POL      --------------------------------------------------------MDDQ 4
PCAT14_orf-52_POL      ------------------------------------------------------------ 100
POL                    PKERVIKTPYQSAQRAELVAVITVLQDFDQPINIISDSAYVVQATRDVETALIKYSMDDQ 540

PCAT14_orf-57_POL      ------------------------------------------------------------ 0
PCAT14_orf-91_POL      LNPLFNLLQQNVRKRNFPFYITHIRAHTNLPGPLTKANEQADLLVSSAFMEAQELHALTH 64
PCAT14_orf-52_POL      ------------------------------------------------------------ 100
POL                    LNQLFNLLQQTVRKRNFPFYITHIRAHTNLPGPLTKANEQADLLVSSALIKAQELHALTH 600

PCAT14_orf-57_POL      ------------------------------------------------------------ 0
PCAT14_orf-91_POL      VNAIGLKNKFDITWKQTKNIVQHCTQCQILHLATQEARVNPRGLCPNVLWQMDVMHVPSF 124
PCAT14_orf-52_POL      ------------------------------------------------------------ 100
POL                    VNAAGLKNKFDVTWKQAKDIVQHCTQCQVLHLPTQEAGVNPRGLCPNALWQMDVTHVPSF 660

PCAT14_orf-57_POL      ------------------------------------------------------------ 0
PCAT14_orf-91_POL      GKLSFVHVTVDTYSHFIWATCQTGESTSHVKRHLLSCFPVMGVPEKVKTDNGPGYCSKAV 184
PCAT14_orf-52_POL      ------------------------------------------------------------ 100
POL                    GRLSYVHVTVDTYSHFIWATCQTGESTSHVKKHLLSCFAVMGVPEKIKTDNGPGYCSKAF 720

PCAT14_orf-57_POL      ----------------------------------------------------MQLNLALY 8
PCAT14_orf-91_POL      QKFLNQWKITHTIGILYNSQGQAIIERTNRTLKAQLVKQKKGKDRSI-TLPRCNLI---- 239
PCAT14_orf-52_POL      ------------------------------------------------------------ 100
POL                    QKFLSQWKISHTTGIPYNSQGQAIVERTNRTLKTQLVKQKEGGDSKECTTPQMQLNLALY 780

PCAT14_orf-57_POL      TLNVLNIYRNQTTTSAEQHLTGKRNSPHEGKLIWWKDNKNKTWEMGKVITWGRGFACVSP 68
PCAT14_orf-91_POL      ------------------------------------------------------------ 239
PCAT14_orf-52_POL      ------------------------------------------------------------ 100
POL                    TLNFLNIYRNQTTTSAEQHLTGKKNSPHEGKLIWWKDNKNKTWEIGKVITWGRGFACVSP 840

PCAT14_orf-57_POL      GENQLPVWIPTRHLKFYNELTGDAKKSVEMET--PQSTRQST-----GVSSSSKETATSK 121
PCAT14_orf-91_POL      ------------------------------------------------------------ 239
PCAT14_orf-52_POL      ------------------------------------------------------------ 100
POL                    GENQLPVWIPTRHLKFYNEPIRDAKKSTSAETETPQSSTVDSQDEQNGDVRRTDEVAIHQ 900

PCAT14_orf-57_POL      NGP-----------------------------------------------------     124
PCAT14_orf-91_POL      --------------------------------------------------------     239
PCAT14_orf-52_POL      --------------------------------------------------------     100
POL                    EGRAADLGTTKEADAVSYKISREHKGDTNPREYAACSLDDCINGGKSPYACRSSCS     956
```

Figure S6

Supplementary Figure S6 – *PCAT14* is the origin of many HERVK-derived peptides

(A) Genome view of the *PCAT14* transcripts detected in hPSCS (top rows), along with the short read RNA-seq pileup data (middle row, blue), the location of TEs (red bars = LTRs, green=SINEs). The lower rows are the GENCODE transcripts. The predicted coding *PCAT14* transcript found in this study is indicated in red.

(B) CLUSTAL-omega alignment of *gag* HERVK viral proteins against putative ORFs from *PCAT14*. All ORFs in all three sense frames were extracted, translated and aligned against the sequence of a putative progenitor HERVK *gag* ORFs. ORFs that aligned to the *gag* viral gene is indicated.

(C) CLUSTAL-omega alignment of *pol* HERVK viral proteins against putative ORFs from *PCAT14*. All ORFs in all three sense frames were extracted, translated and aligned against the sequence of a putative progenitor HERVK *pol* ORF. ORFs that aligned to the *pol* viral gene is indicated.

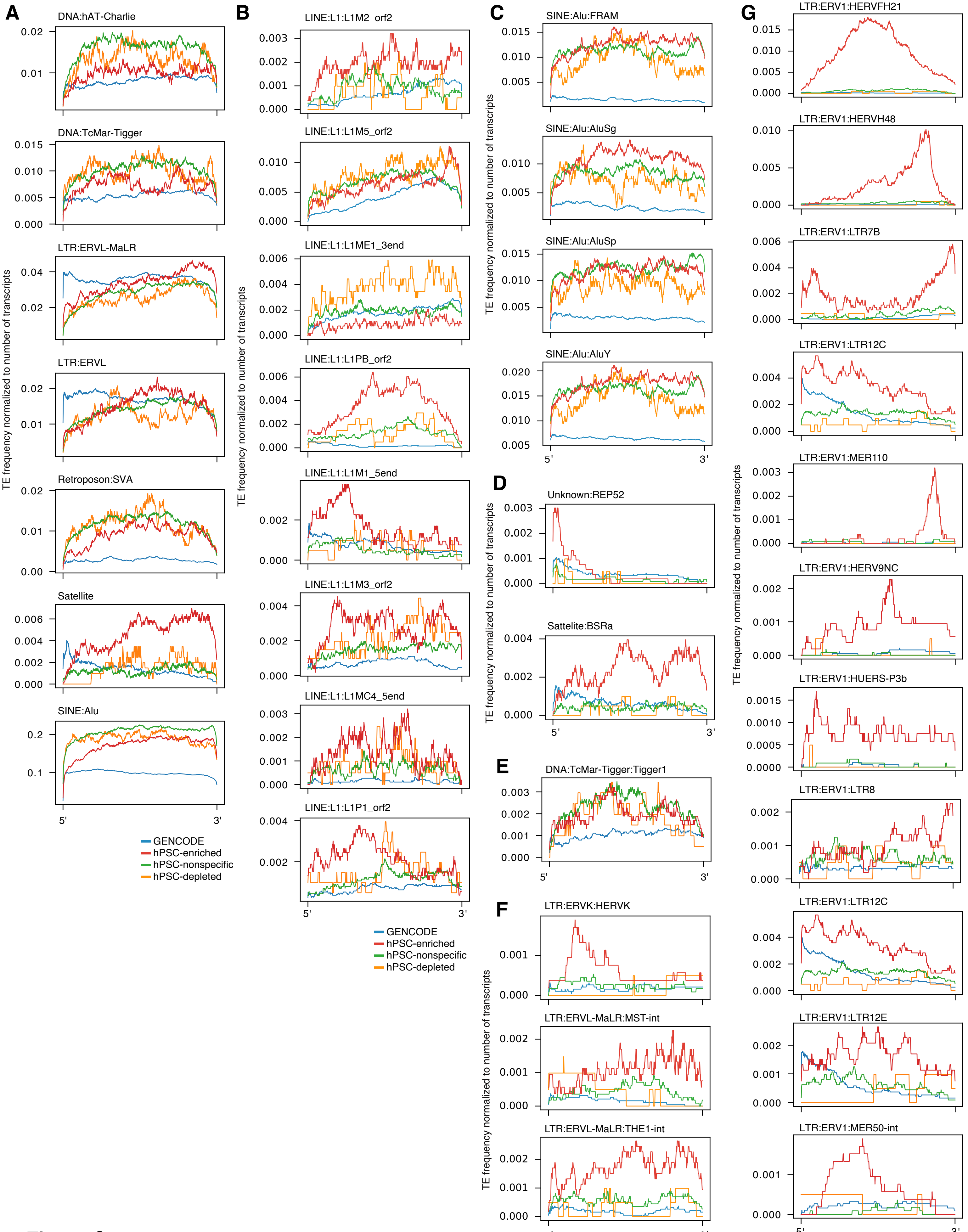
A
DNA:hAT-Charlie
DNA:TcMar-Tigger
LTR:ERVL-MaLR
LTR:ERVL
Retroposon:SVA
Satellite
SINE:Alu
TE frequency normalized to number of transcripts
5'
3'
GENCODE
hPSC-enriched
hPSC-nonspecific
hPSC-depleted
B
LINE:L1:L1M2_orf2
LINE:L1:L1M5_orf2
LINE:L1:L1ME1_3end
LINE:L1:L1PB_orf2
LINE:L1:L1M1_5end
LINE:L1:L1M3_orf2
LINE:L1:L1MC4_5end
LINE:L1:L1P1_orf2
TE frequency normalized to number of transcripts
5'
3'
GENCODE
hPSC-enriched
hPSC-nonspecific
hPSC-depleted
C
SINE:Alu:FRAM
SINE:Alu:AluSg
SINE:Alu:AluSp
SINE:Alu:AluY
TE frequency normalized to number of transcripts
5'
3'
D
Unknown:REP52
Sattelite:BSRa
TE frequency normalized to number of transcripts
5'
3'
E
DNA:TcMar-Tigger:Tigger1
5'
3'
F
LTR:ERVK:HERVK
LTR:ERVL-MaLR:MST-int
LTR:ERVL-MaLR:THE1-int
TE frequency normalized to number of transcripts
5'
3'
G
LTR:ERV1:HERVFH21
LTR:ERV1:HERVH48
LTR:ERV1:LTR7B
LTR:ERV1:LTR12C
LTR:ERV1:MER110
LTR:ERV1:HERV9NC
LTR:ERV1:HUERS-P3b
LTR:ERV1:LTR8
LTR:ERV1:LTR12C
LTR:ERV1:LTR12E
LTR:ERV1:MER50-int
TE frequency normalized to number of transcripts
5'
3'

Figure S7

Supplementary Figure S7 – Density of TE insertions across lncRNAs.

(A) Frequency of TEs subtypes across the normalized length of all lncRNAs, from the 5’ ends of the transcript to the 3’ ends. The transcript lengths are normalized to the same length. TE frequency is normalized to the total number of transcripts in each expression class. The expression classes are hPSC-enriched transcripts (more likely to be specific to hPSCs), -nonspecific (found in most cell types) and -depleted (expressed in hPSCs, but more likely to have higher RNA levels in other cell types).

(B) As in panel A, but for LINE families.

(C) As in panel A, but for SINE families.

(D) As in panel A, but for Unknown and Satellite families.

(E) As in panel A, but for the DNA TE Tigger1.

(F) As in panel A, but for ERVK and ERVL-MaLR TEs.

(G) As in panel A, but for ERV1 LTRs.

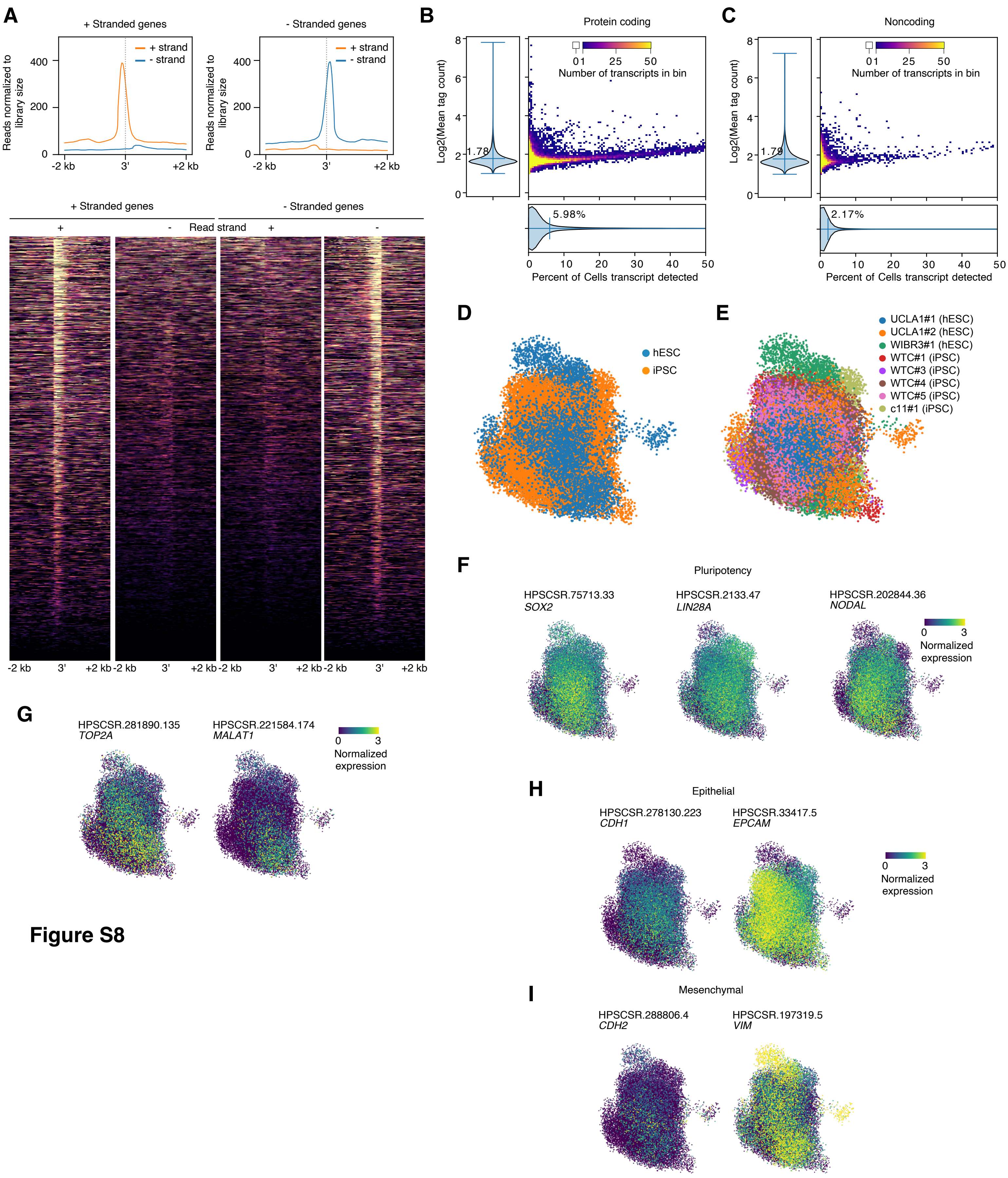
A
+ Stranded genes
- Stranded genes
Reads normalized to library size
+ strand
- strand
400
200
0
-2 kb
3'
+2 kb
Read strand
B
Protein coding
Log2(Mean tag count)
Number of transcripts in bin
1.78
5.98%
Percent of Cells transcript detected
C
Noncoding
1.79
2.17%
D
hESC
iPSC
E
UCLA1#1 (hESC)
UCLA1#2 (hESC)
WIBR3#1 (hESC)
WTC#1 (iPSC)
WTC#3 (iPSC)
WTC#4 (iPSC)
WTC#5 (iPSC)
c11#1 (iPSC)
F
Pluripotency
HPSCSR.75713.33
SOX2
HPSCSR.2133.47
LIN28A
HPSCSR.202844.36
NODAL
Normalized expression
G
HPSCSR.281890.135
TOP2A
HPSCSR.221584.174
MALAT1
H
Epithelial
HPSCSR.278130.223
CDH1
HPSCSR.33417.5
EPCAM
I
Mesenchymal
HPSCSR.288806.4
CDH2
HPSCSR.197319.5
VIM

Figure S8

Supplementary Fig. S8 – Analysis of the sc-RNA-seq data.

(A) Pileups and heatmaps of sc-RNA-seq reads at the 3' ends of the transcripts. The pileups indicate the signal for + stranded transcripts on both the + and – strand (left pileups), and the same for the – stranded transcripts (right-hand pileups). The pileups are centered on the 3' end of the transcript and extend 2 kb in either direction. The lower heatmaps show the same data as the pileups, but each row is a transcript.

(B) 2D heatmap of all detectable coding transcripts in the sc-RNA-seq data. The 2D heatmap shows the density of the transcripts in each 2D bin, and the axes show the mean tag count (y-axis) versus the percent of cells the transcript could be detected in (x-axis). Violin plots show the density of transcripts along each axis, and the mean of each axis is indicated.

(C) As in panel B, but showing noncoding transcripts.

(D) UMAP plots colored according to the origin of the cell: whether it was a hESC (blue) or an iPSC (orange) in the UMAP plot.

(E) UMAP plot colored by the sample that the cell originated from. Samples are named according to the cell line they were identified as. WIBR3 and c11 (S0730) lines are from this study, the two UCLA lines are from GSE140021 (4), and the WTC lines are from E-MTAB-6687 (3).

(F) UMAP plots colored by normalized expression. The expression of the hPSC-specific genes *SOX2*, *LIN28A*, and *NODAL* are shown.

(G) As in panel F, but for proliferation-related transcripts *TOP2A*, and *MALAT1*

(H) As in panel F, but for epithelial transcripts *CDH1*, and *EPCAM*.

(I) As in panel F, but for mesenchymal transcripts *CDH2*, and *VIM*.

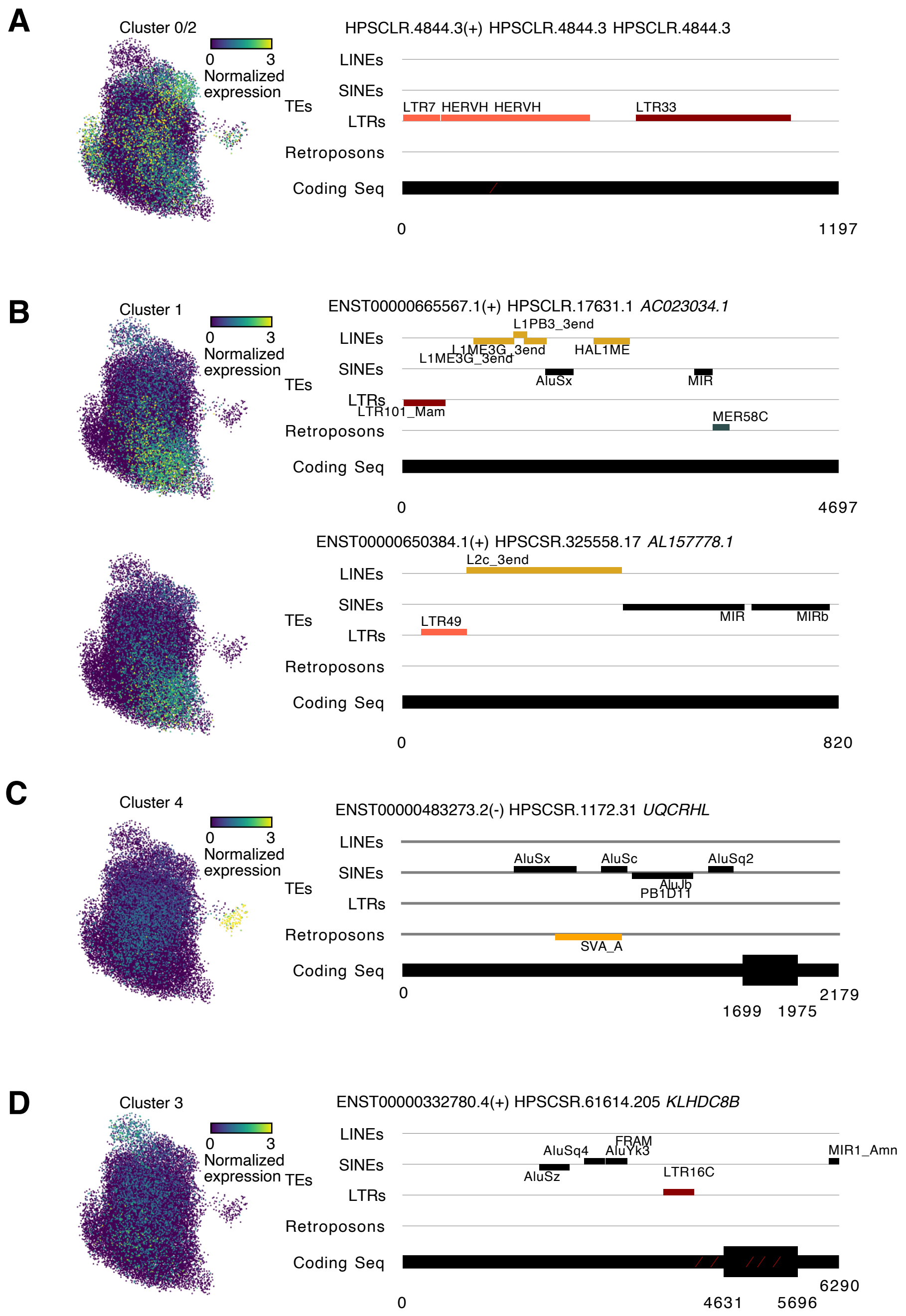
A
Cluster 0/2
0 3
Normalized expression
HPSCLR.4844.3(+) HPSCLR.4844.3 HPSCLR.4844.3
LINEs
SINEs
TEs
LTRs
Retroposons
Coding Seq
LTR7 HERVH HERVH
LTR33
0
1197
B
Cluster 1
ENST00000665567.1(+) HPSCLR.17631.1 AC023034.1
L1PB3_3end
L1ME3G_3end
L1ME3G_3end
HAL1ME
AluSx
MIR
LTR101_Mam
MER58C
4697
ENST00000650384.1(+) HPSCSR.325558.17 AL157778.1
L2c_3end
MIR
MIRb
LTR49
820
C
Cluster 4
ENST00000483273.2(-) HPSCSR.1172.31 UQCRHL
AluSx
AluSc
AluSq2
AluJb
PB1D11
SVA_A
2179
1699 1975
D
Cluster 3
ENST00000332780.4(+) HPSCSR.61614.205 KLHDC8B
FRAM
AluSq4
AluYk3
MIR1_Amn
AluSz
LTR16C
6290
4631 5696

Figure S9

Supplementary Fig. S9 – Example TE-containing transcripts expressed in subpopulations of cells in hPSC cultures.

(A) UMAP plots (left) colored by transcript expression for a novel noncoding cluster 0/2 gene (HPSCLR.4844.3). The domain plot is shown on the right-hand side, it indicates the location of the TE sequences in the transcript. The 'coding seq' part indicates the presence or absence of a CDS (this transcript is predicted to be non coding). The locations of LINEs, SINEs, LTRs and Retroposons are indicated if present and the full length of the transcript is marked at the bottom of the plot.

(B) As in panel A, but for cluster 1-specific transcripts. The two examples shown here are noncoding transcripts, containing multiple TE fragments, particularly LINEs, SINEs and LTRs. As this transcript matches an ENCODE transcript its gene symbol and Ensembl transcript ID is indicated.

(C) As in panel B, but for a cluster 4-specific transcript. The thick part of the 'coding seq' indicates the CDS, the thin part is the noncoding UTRs.

(D) As in panel B, but for a cluster 3-specific transcript. The thick part of the 'coding seq' indicates the CDS, the thin part is the noncoding UTRs.

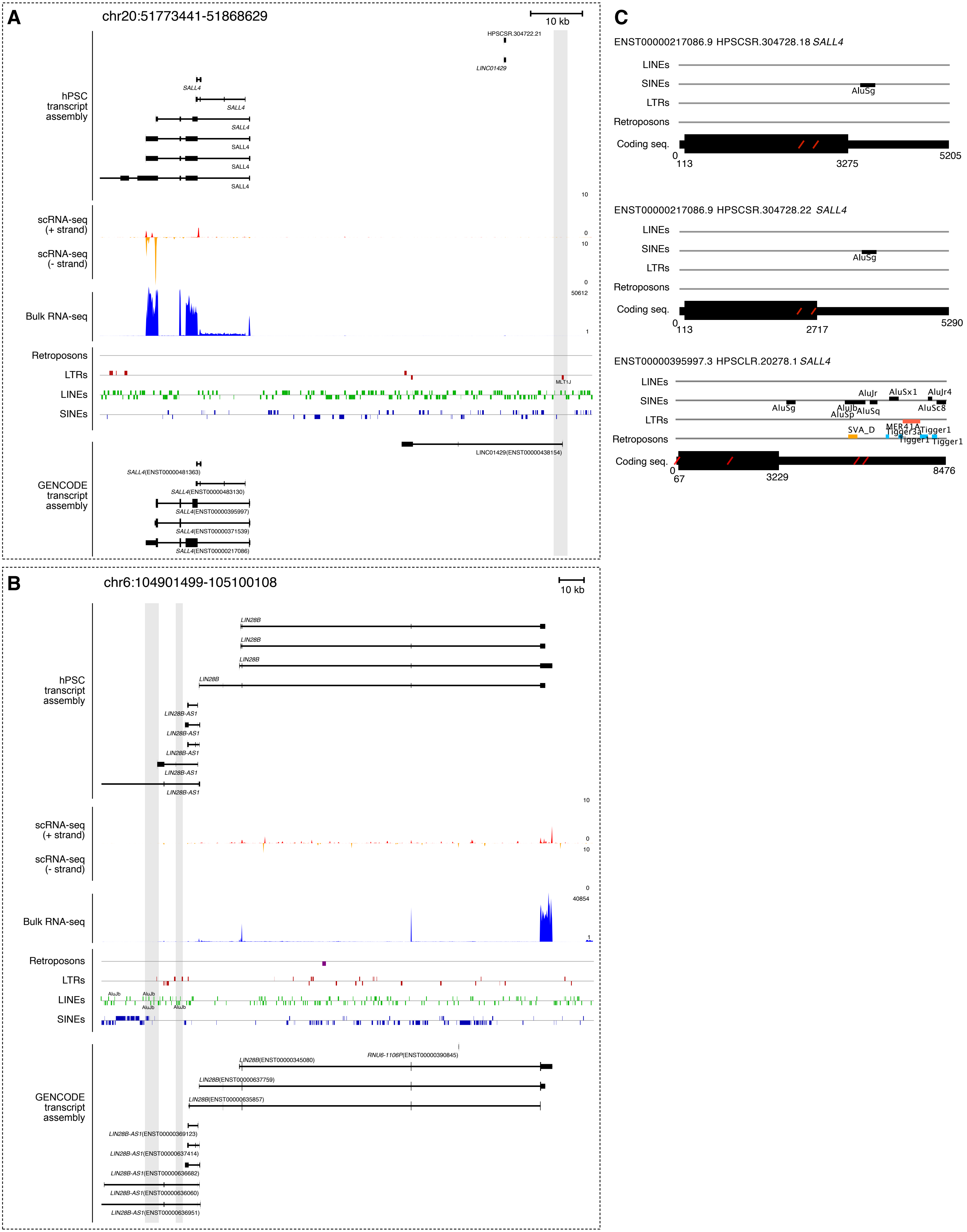
A
chr20:51773441-51868629
10 kb
HPSCSR.304722.21
LINC01429
SALL4
hPSC transcript assembly
scRNA-seq (+ strand)
scRNA-seq (- strand)
Bulk RNA-seq
50612
Retroposons
LTRs
LINEs
SINEs
MLT1J
LINC01429(ENST00000438154)
GENCODE transcript assembly
SALL4(ENST00000481363)
SALL4(ENST00000483130)
SALL4(ENST00000395997)
SALL4(ENST00000371539)
SALL4(ENST00000217086)
B
chr6:104901499-105100108
10 kb
LIN28B
LIN28B-AS1
40854
AluJb
RNU6-1106P(ENST00000390845)
LIN28B(ENST00000345080)
LIN28B(ENST00000637759)
LIN28B(ENST00000635857)
LIN28B-AS1(ENST00000369123)
LIN28B-AS1(ENST00000637414)
LIN28B-AS1(ENST00000636682)
LIN28B-AS1(ENST00000636060)
LIN28B-AS1(ENST00000636951)
C
ENST00000217086.9 HPSCSR.304728.18 SALL4
Coding seq.
AluSg
0
113
3275
5205
ENST00000217086.9 HPSCSR.304728.22 SALL4
0
113
2717
5290
ENST00000395997.3 HPSCLR.20278.1 SALL4
AluSg
AluJr
AluSx1
AluJr4
AluJb
AluSp
AluSq
AluSc8
SVA_D
MER41A
Tigger3a
Tigger1
0
67
3229
8476

Figure S10

Supplementary Fig. S10 – Two pluripotency genes detected containing a TE in the first exon in cancer cells, *SALL4* and *LIN28B*, were not assembled in hPSCs.

(a) Genome views containing the transcripts assembled in this study (top), read pileups for the sc-RNA-seq (red and orange pileups, for the positive strand and negative strand, respectively), the short read (bulk) RNA-seq pileup data (blue), and the locations of LINEs, SINEs, LTRs and Retroposons in the genome. The GENCODE transcripts are shown below. The grey box indicates the position of the MLT1J that acts as a promoter, TSS and part of the first exon of a modified *SALL4* transcript detected in cancerous cells. From Ref. (10).

(b) As in panel A, but showing the *LIN28B* locus. The location of the Alu's that function as a promoter, TSS and part of the first exon of a modified *LIN28B* as detected in Ref. (10) is indicated.

(c) All of the domain maps for the three TE-containing *SALL4* transcripts detected in this study in hPSCs. The location of TEs in the LINEs, SINEs, LTRs or DNA/SVAs are indicated. The CDS is indicted by a think black box, and the numbers below the 'coding seq' indicate the start of the indicated regions in mRNA coordinates. Locations of splice sites are indicated by a slanted red line.
